# Comparing progression molecular mechanisms between lung adenocarcinoma and lung squamous cell carcinoma based on genetic and epigenetic networks: big data mining and genome-wide systems identification

**DOI:** 10.18632/oncotarget.26940

**Published:** 2019-06-04

**Authors:** Shan-Ju Yeh, Chien-An Chang, Cheng-Wei Li, Lily Hui-Ching Wang, Bor-Sen Chen

**Affiliations:** ^1^ Laboratory of Automatic Control, Signaling Processing, and Systems Biology, Department of Electrical Engineering, National Tsing Hua University, Hsinchu 30013, Taiwan; ^2^ Department of Medical Science, Institute of Molecular and Cellular Biology, National Tsing Hua University, Hsinchu 30013, Taiwan; ^3^ Department of Electrical Engineering, Yuan Ze University, Chungli 32003, Taiwan

**Keywords:** lung adenocarcinoma, lung squamous cell carcinoma, NSCLC, genetic and epigenetic network, potential drug target

## Abstract

Non-small-cell lung cancer (NSCLC) is the predominant type of lung cancer in the world. Lung adenocarcinoma (LADC) and lung squamous cell carcinoma (LSCC) are subtypes of NSCLC. We usually regard them as different disease due to their unique molecular characteristics, distinct cells of origin and dissimilar clinical response. However, the differences of genetic and epigenetic progression mechanism between LADC and LSCC are complicated to analyze. Therefore, we applied systems biology approaches and big databases mining to construct genetic and epigenetic networks (GENs) with next-generation sequencing data of LADC and LSCC. In order to obtain the real GENs, system identification and system order detection are conducted on gene regulatory networks (GRNs) and protein-protein interaction networks (PPINs) for each stage of LADC and LSCC. The core GENs were extracted via principal network projection (PNP). Based on the ranking of projection values, we got the core pathways in respect of KEGG pathway. Compared with the core pathways, we found significant differences between microenvironments, dysregulations of miRNAs, epigenetic modifications on certain signaling transduction proteins and target genes in each stage of LADC and LSCC. Finally, we proposed six genetic and epigenetic multiple-molecule drugs to target essential biomarkers in each progression stage of LADC and LSCC, respectively.

## INTRODUCTION

Lung cancer is the most common malignancy resulting in the largest number of cancer-related deaths worldwide [1, 2]. Only 15.9% of lung cancer patients survive more than 5 years [3]. Lung cancers are classified by histological types which have something to do with important implications for the clinical management and prognosis of the disease. There are two main histological groups of lung cancer which are non-small-cell lung cancer (NSCLC) and small-cell lung cancer. About 85% of human lung cancers are non-small cell lung cancer [4]. NSCLCs are subdivided broadly into three major histological subtypes: lung adenocarcinoma (LADC), lung squamous cell carcinoma (LSCC), also called epidermoid carcinoma, and lung large-cell carcinoma (LLCC). Both LADC and LSCC are the predominant form of lung cancer accounting for the 40% and 33% majority of cancer deaths worldwide, respectively [5, 6].

Based on histopathological features and gene expression signatures, LADC and LSCC are widely thought to be characterized by a different cell of origin and a distinct pattern of molecular alterations. In general, for LADCs, which are thought to origin from the bronchiolar or alveolar epithelium (Clara cells or type II pneumocytes), mainly arise from the peripherally located airways and can be characterized by the production of mucin and/or the occurrence of glandular differentiation. For LSCCs, which typically often origin from the bronchial epithelium of larger and more central airways (basal cells), mostly arise from central lung and can be characterized by the morphological features of squamous differentiation, including squamous pearl formation, individual cell keratinisation, and intercellular bridges [5, 7–9].

One major risk factor of these two histologies is tobacco smoke and the other risk factors are asbestos, radon, and environmental/occupational exposure to polycyclic aromatic hydrocarbons and other pollutants contributing to the development of lung cancer, including LADC and LSCC [10, 11]. It has been reported that LADCs often occur in women and people who don’t have smoke history, while LSCCs are highly correlated with exposure to tobacco smoke [5, 8, 12]. Though some molecular attributes are shared in these two subtypes, they represent unique molecular characteristics, distinct cells of origin, and different clinical responses [10]. Hence, LADC and LSCC could be seen as distinct diseases. However, the molecular mechanisms underlying carcinogenesis and tumor development of LADC and LSCC are not fully elucidated. Therefore, it is important to understand the carcinogenic mechanism of LADC and LSCC, which could provide more efficacious therapeutic strategies.

Recent studies indicate that carcinogenesis and cancer development not only be accounted for genetic alterations alone, but also involve epigenetic changes. Both genetic alterations and epigenetic changes could lead to the dysregulation of key tumor suppressor genes, oncogenes, and DNA repair/housekeeping genes. Despite the well-known role of genetic alterations, including copy number alterations and mutations in oncogenesis, it has been reported that epigenetic changes are much more frequent than genetic alterations in lung cancer [10, 13]. The epigenetic changes include DNA methylation, histone modifications, and non-coding RNA, specific microRNA and lncRNA [10, 14]. Histone modifications and DNA methylation, which are important process for regulating gene transcription, play important roles in maintaining the normal development of cells. However, the anomalies in DNA methylation and histone modifications are associated with the carcinogenesis and development of tumor cells. It has been observed that DNA methylation was associated with tissue specificity and distinct DNA methylation patterns for LADC and LSCC have been identified [15–17].

MicroRNAs (miRNAs) are short non-coding RNAs with 19–25 nucleotides (nts) in length. They mediate post-transcriptional control of gene expression by incorporating with the RNA-induced silencing complex (RISC) and partially or perfectly bind to the 3′ untranslated regions (3′-UTR) of target mRNA leading to either degradation of their target mRNAs or translational repression [18]. Experimental and bioinformatic studies have shown that more than 30% of human genes are direct targets of miRNA, implying that miRNAs are involved in the regulation of various biological processes including cell cycle regulation, cell growth, apoptosis, cell differentiation and stress reactions [14]. Previous study proposed a framework utilizing functional annotation information to identify coregulation between transcriptional and post-transcriptional layers [19]. Calin *et al*. have demonstrated that the locations of miRNA genes are frequently found at fragile sites in the genome, suggesting that the aberrant expression of miRNAs is associated with tumorigenesis [20]. Indeed, accumulating evidences show that the dysregulations of miRNAs are observed in human cancers, including NSCLC (LADC and LSCC), and may serve as oncogenes or tumor suppressors [21, 22].

In addition to miRNAs, long non-coding RNAs (lncRNAs) also have been caught the attention in recent year due to its role in the regulation of cancer development. LncRNA, non-protein-coding transcript that is longer than 200 nucleotides, can serve as regulators of gene expression and chromatin structure [23]. Numerous evidences showed that the expression of lncRNAs could alter various types of human cancers, including NSCLC (LADC and LSCC). It implies that the differential expressions of lncRNAs are associated with cancer pathogenesis and function as new regulators in cancer development [24, 25]. Moreover, it has been found that the binding affinities of transcription factors (TFs), RNA polymerase, miRNAs, and lncRNAs could be affected by DNA methylation [26, 27]. Therefore, analyzing the regulation of miRNAs and lncRNAs in progression molecular mechanisms of LADC and LSCC is essential as well.

In recent year, the protein-protein interaction networks (PPINs) and gene regulatory network (GRNs) have been used to investigate the molecular mechanisms and found target genes based biomarkers [28, 29]. Focusing on global human gene regulatory network comprising both transcriptional and post-transcriptional regulatory relationships, and integrated the protein interactome, the topological properties for regulatory motifs has been investigated to reveal target crucial proteins [30]. Moreover, there is a study proposing computational method to predict lncRNA functions by identifying lncRNA-associated modules in protein-protein interaction network [31]. Gene expression profiles in different tissues could be used to discover disease-specific biomarker genes by evaluating Pearson correlation coefficient and Kolmogorov-Smirnov distance [32]. To date, many studies used gene and protein expression profiles with computational methods to identify new drug targets and biomarkers for drug discovery [33, 34]. Based on the examination of genes for tissue- and gene-specific correlations, a large-scale analysis of protein abundance and gene expression across a diverse set of human tissues was proposed [35]. However, seldom research would consider epigenetic modifications effects to panoramic view of an extensive network. Therefore, in this study, we put focus on the comparison of progression molecular mechanisms and how epigenetic regulations and modifications contribute to the development of LADC and LSCC, respectively.

In order to construct real GRNs and PPIs for real GENs in each stage of LADC and LSCC (normal stage, early stage, middle stage, and advanced stage), we have applied system modeling, system identification, system order detection scheme and big database mining approaches on their corresponding next-generation sequencing (NGS) data. Since the real GENs are still too complicated to analyze, the core networks should be extracted from the real GENs. Previous literature has shown that network connectivity was predictive of function because interactions often occurred among functionally related genes [36]. However, core network was constructed by identified network hubs and core elements not in hubs would be neglected [37]. Here, we can use the principal network projection (PNP) method on genome-wide data to avoid this problem to extract the principal structure of real GENs from the perspective of significant network linking energy. By comparing the core GENs between two connective stages (normal to early stage, early to middle stage, middle to advanced stage) of LADC and LSCC, we respectively obtained differential core signaling pathways of LADC and LSCC to get an insight into the differential genetic and epigenetic progression mechanisms. Based on the analysis of core signaling pathways in each stage of LADC and LSCC, we found different microenvironment changes, dysregulation of miRNAs/lncRNAs, DNA methylation and epigenetic modifications would involve in the mechanisms of LADC and LSCC progression. Finally, we selected some significant network biomarkers as drug targets and proposed six genetic and epigenetic multiple-molecule drugs for early, middle, and advanced stage of LADC and LSCC to prevent disease progression, respectively.

## RESULTS

**Figure 1:**
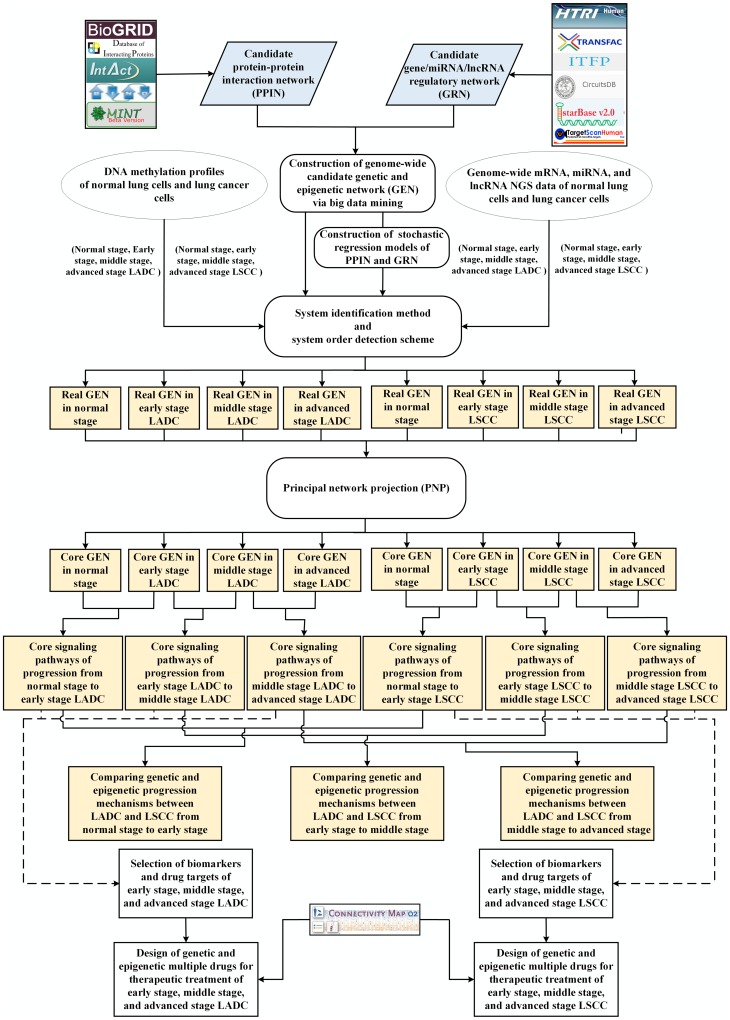
Flowchart of the construction for genome-wide GENS, core GENs, and core signaling pathways of each progression stage of LADC and LSCC and the discovery of potential genetic and epigenetic multiple drugs. The oval blocks represent the raw data of normal lung cells and lung cancer cells (i.e. early stage, middle stage, and advanced stage of LADC and LSCC), including genome-wide mRNA/miRNA/lncRNA NGS data and DNA methylation profiles. The blue grey blocks denote the candidate protein-protein interaction network (PPIN), which was constructed by databases (BIOGRID, IntAct, DIP, BIND, and MINT), and the candidate gene/miRNA/lncRNA regulatory network (GRN), which was constructed by databases (ITFP, HTRIdb, TRANSFAC, TargetScanHuman, CircuitDB, and StarBase2.0). The rounded rectangular blocks indicate the systems biology approach to be applied to construct genome-wide candidate GEN, real GENs of each stage (normal lung cells, early stage, middle stage, and advanced stage) of LADC and LSCC, and then to obtain core pathways of LADC and LSCC at each progression stage (normal cells to early stage, early stage to middle stage, and middle stage to advanced stage) by comparing the core GENs among the different stage. The light yellow blocks represent the identified information in our results, including real GENs at each stage and core signaling pathways of each progression stage. Besides, we selected biomarkers and drugtargets based on our results (dashed line with arrow) for designing genetic and epigenetic multiple drugs via CMap drug database mining for therapeutic treatment of early stage, middle stage, and advanced stage in LADC and LSCC, respectively.

In this study, a flowchart for constructing genome-wide GENs, core GENs, and core signaling pathways of each progression stage of LADC and LSCC is shown in Figure 1. To further investigate the different genetic and epigenetic mechanisms between LADC and LSCC from normal stage to early stage, early stage to middle stage, and middle stage to advanced stage, we respectively constructed the genome-wide real GENs of each stage of LADC and LSCC by big databases mining, system modeling, and systematic analysis. Utilized the network visualizing software Cytoscape [38], the genome-wide real GENs of each stage of LADC and LSCC are shown in Supplementary Figures 1, 2, respectively. Besides, the numbers of identified nodes and edges of each stage of LADC and LSCC are shown in Supplementary Tables 1, 2, respectively.

However, the genome-wide real GENs are still too complicated. Based on the PNP method, we considered the projection value (i.e., *D_L_*(*k*) and *D_R_*(*t*) at equation (38) in the Supplementary Materials) and extracted the core GENs shown in Supplementary Figures 3, 4 from the real GENs for each stage of LADC and LSCC, respectively. Here, we selected human proteins, which do not include TFs with the top 3000 projection values and human TFs with the top 300 projection values in all four stages of LADC and LSCC. Besides, miRNAs/lncRNAs, which connect to genes encoding those selected proteins and TFs, were also selected as core nodes of each stage. The core GENs of normal stage, early stage, middle stage, and advanced stage of LADC and LSCC are shown in Supplementary Figures 3, 4, respectively.

**Figure 2:**
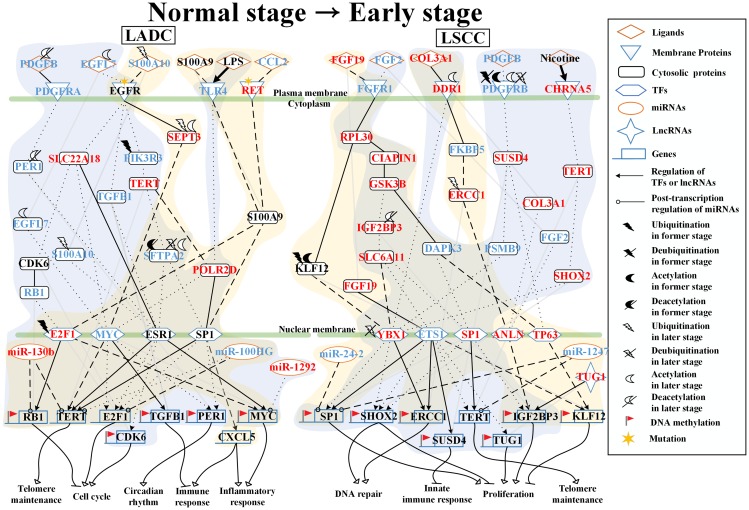
Core signaling pathways extracted from comparing genetic and epigenetic networks (GENs) between normal lung cells and early stage LADC and LSCC. The dot and dashed line represent the identified signaling pathways in former stage (normal stage) and later stage (early stage), respectively. Solid line indicates the common signaling pathways identified in both former stage and later stage. The yellow and blue regions are former stage (normal stage) and later stage (early stage), respectively. The lines without arrow denote the protein-protein interactions (PPIs). The lines with arrow represent the regulations of TFs and lncRNAs with activation and inhibition. The lines with circle are post-transcription regulations of miRNA with inhibition. Besides, the bold lines with arrow indicate the interaction or stimulation of proteins and xenobiotics. The Red font represent the node with significant differential expression change with a higher expression in later stage LADC and LSCC. While the blue font represent the node with a significant differential expression change with a lower expression in later stage LADC and LSCC. Besides, the gene with flag represents that this gene has basal level change between former and later stage LADC and LSCC, suggesting that the gene may be affected by DNA methylation.

**Figure 3:**
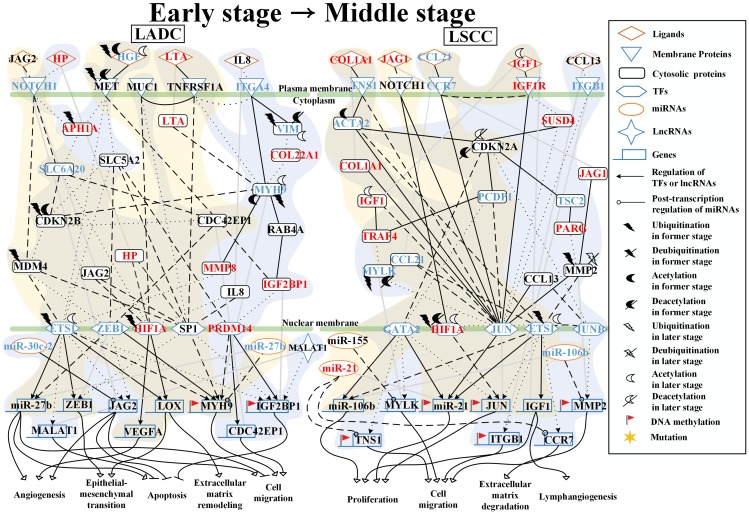
Core signaling pathways extracted from comparing genetic and epigenetic networks (GENs) between early stage and middle stage LADC and LSCC. The dot and dashed line represent the identified signaling pathways in former stage (early stage) and later stage (middle stage), respectively. Solid line indicates the common signaling pathways identified in both former stage and later stage. The yellow and blue regions are former stage (early stage) and later stage (middle stage), respectively. The lines without arrow denote the protein-protein interactions (PPIs). The lines with arrow represent the regulations of TFs and lncRNAs with activation and inhibition. The lines with circle are post-transcription regulations of miRNA with inhibition. The Red font represent the node with significant differential expression change with a higher expression in later stage LADC and LSCC. While the blue font represent the node with a significant differential expression change with a lower expression in later stage LADC and LSCC. Besides, the gene with flag represents that this gene has basal level change between former and later stage LADC and LSCC, suggesting that the gene may be affected by DNA methylation.

**Figure 4:**
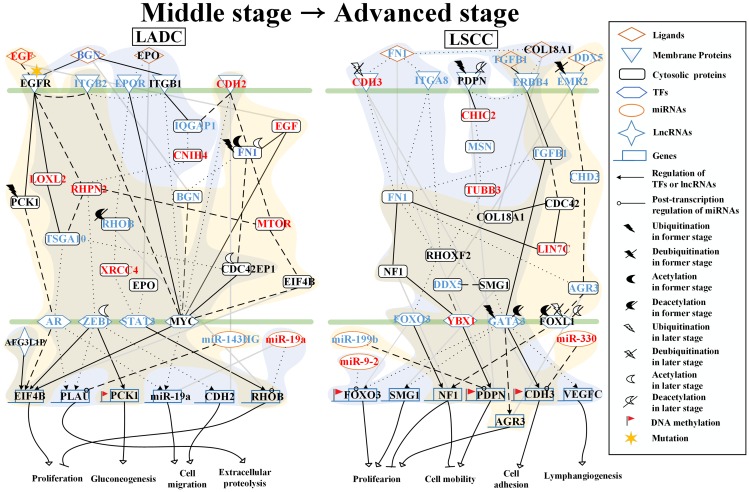
Core signaling pathways extracted from comparing genetic and epigenetic networks (GENs) between middle stage and advanced stage LADC and LSCC. The dot and dashed line represent the identified signaling pathways in former stage (middle stage) and later stage (advanced stage), respectively. Solid line indicates the common signaling pathways identified in both former stage and later stage. The yellow and blue regions are former stage (middle stage) and later stage (advanced stage), respectively. The lines without arrow denote the protein-protein interactions (PPIs). The lines with arrow represent the regulations of TFs and lncRNAs with activation and inhibition. The lines with circle are post-transcription regulations of miRNA with inhibition. The Red font represent the node with significant differential expression change with a higher expression in later stage LADC and LSCC. While the blue font represent the node with a significant differential expression change with a lower expression in later stage LADC and LSCC. Besides, the gene with flag represents that this gene has basal level change between former and later stage LADC and LSCC, suggesting that the gene may be affected by DNA methylation.

To further compared and investigated genetic and epigenetic progression mechanisms between LADC and LSCC from each progression stage, we constructed core signaling pathways in different stages of LADC and LSCC. Core signaling pathways start from core receptors passing through several core signal transduction proteins to the core TFs which would regulate corresponding core proteins, lncRNAs and miRNAs. The expression changes of these core elements are marked in the denotation of KEGG signal transduction pathways in each LADC and LSCC progression stage. Besides, we considered epigenetic modifications on proteins, such as ubiquitination, deubiquitination, acetylation, and deacetylation. These epigenetic modifications could be attributed to the changes of the basal level of *b_j_* between former stage and later stage in the stochastic protein interactive model of the PPIN in equation (1) (see Materials and Methods). With the basal level change between two connective stages over a PPI basal level threshold, the core proteins were postulated to be affected by some epigenetic modifications. In addition, in specific core signaling pathways, we only considered the epigenetic modification induced by different epigenetic proteins between two connective stages. If the expression of epigenetic protein has the lowest value within 4 stages of LADC and LSCC, the epigenetic protein will not be considered. Moreover, the genes with basal level changes which are higher than a threshold between two connective stages suggest that they are affected by DNA methylation. The core signaling pathways of each carcinogenic progression stage (normal stage to early stage, early stage to middle stage, and middle stage to advanced stage) are described in the followings and shown in Figures 2–4.

### Analysis of core pathways to investigate different genetic and epigenetic progression mechanisms of LADC and LSCC from normal stage to early stage

As shown in Figure 2, in normal stage of lung cells adjacent to the LADC, the receptors TLR4 and RET both interact with pro-inflammatory factor S100A9 and are activated by Lipopolysaccharide (LPS) and chemokine CCL2 to trigger protein S100A9 to modulate TFs, E2F1 and Sp1. The TF E2F1 affected by HECW2-induced ubiquitination negatively regulates inflammation-related gene *MYC* and cell cycle-related gene *RB1*. TF Sp1 negatively regulates telomere-related gene *TERT* and regulates gene *CXCL5*, involved in immune and inflammatory response. After receiving signaling from pro-inflammatory factor S100A10, the receptor EGFR then triggers the TF MYC mediated by cell cycle-related protein SEPT3, which belongs to the septin family of GTPases. The TF MYC then negatively regulates telomere-related gene *TERT* and immune-related genes *TGFB1*.

In early stage LADC, after receiving the signaling from PDGFB affected by HDAC4-induced deacetylation, the receptor PDGFRA triggers TF ESR1 mediated by protein PER1 affected by HDAC11-induced deacetylation. The TF ERα then negatively regulates cell cycle-related genes, *E2F1* and *CDK6*. In addition, through interacting with EGFL7 affected by ELP3-induced acetylation, receptor EGFR then triggers the TFs, E2F1, c-Myc, ERα, and Sp1, mediated by proteins, SLC22A18, CDK6, RB1, TGFB1, PIK3R3, TERT, and SFTPA2, which is affected by USP19-induced deubiquitination and KAT5-induced acetylation. The TF E2F1 negatively regulates cell cycle-related gene *RB1* and the TF MYC negatively regulates cell cycle-related gene *E2F1* and circadian rhythm-related gene *PER1*. The TF ERα negatively regulates telomere-related gene *TERT* and positively regulates *MYC*, and the TF Sp1 positively regulates the telomere-related gene *TERT* and negatively regulates cell cycle-related gene *E2F1*. Besides, the receptor TLR4 activated by LPS triggers TFs, c-Myc and Sp1. TF c-Myc positively regulates immune-related gene *TGFB1*. TF Sp1 positively regulates circadian-related gene *PER1*. Moreover, the receptor TLR4 modulates TF MYC mediated by POLR2D to regulate immune-related gene *TGFB1*.

Besides, in core signaling pathways between normal stage and early stage LADC, we also identified miRNAs, including miR-130b, miR-100HG, and miR-1292 (Figure 2). MiR-130b inhibits *RB1* and *TERT* in normal stage and has a higher expression in early stage (*p*-value < 5.410^–5^). MiR-100HG inhibits *E2F1* in normal stage and *TERT* in early stage of LADC and has a higher expression in normal stage (*p*-value < 1.1 × 10^–2^). MiR-1292 inhibits MYC in the early stage of LADC and has a higher expression in the early stage of LADC (*p*-value < 9.4 × 10^–6^). Moreover, there are five genes, *RB1*, *CDK6*, *TGFB1*, *PER1*, *MYC*, with basal level difference than a threshold between normal stage and early stage LADC, suggesting that this might have been caused by DNA methylation on the corresponding genes. Our results indicate that in the normal stage of lung cells adjacent to the LADC, inflammatory response and immune response are caused by the mediation of genes, *MYC* and *CXCL5*, and mediation of genes, *TGFB1* and *CXCL5*, respectively. Telomere maintenance and cell cycle are inhibited by the mediation of gene *TERT* and mediation of genes, *RB1* and *MYC*, respectively. However, in the early stage of LADC, telomere maintenance and cell cycle are caused by the mediation of gene *TERT* and mediation of genes, *RB1, E2F1, CDK6, and MYC*, respectively. Immune response and circadian rhythm are inhibited by the mediation of gene *TGFB1* and mediation of gene *PER1*, respectively.

In the normal stage of lung cells adjacent to the LSCC, the receptor FGFR1 is activated by fibroblast growth factor FGF19 to modulate TF YBX1 to positively regulate DNA repair-related gene *ERCC1* through proteins RPL30 and KLF12 affected by the UBE2J1-induced ubiquitination and KAT7-induced acetylation (Figure 2). Besides, receptor FGFR1 also modulates TF TP63 to positively regulate proliferation-related gene *KLF12*. After binding to collagen COL3A1, receptor DDR1 then triggers the TF ANLN to negatively regulate proliferation-related gene *IGF2BP3* by the mediation of proteins FKBP5 and ERCC1 affected by BTRC-induced ubiquitination in early stage LSCC.

In the early stage of LSCC, the receptor FGFR1 binds to FGF2 to modulate TFs ETS1 and Sp1 through the mediation of proteins RPL30, CIAPIN1, GSK3B, IGF2BP3, SLC6A11, and DAPK3. TF ETS1 then positively regulates proliferation-related gene *SP1* and negatively regulates DNA repair-related genes *ERCC1* and *SHOX2*, telomere-related gene *TERT*, proliferation-related gene *TUG1*, and gene *SUSD4* involved in the innate immune response. The receptor DDR1, which is affected by SAT1-induced acetylation, interacts with collagen COL3A1 to directly trigger TF YBX1. Besides, PDGFRB and CHRNA5 are respectively activated by ligand PDGFB and xenobiotic nicotine to modulate TF YB-1 through proteins SUSD4 and PSMB9 and through proteins TERT and SHOX2, respectively. Then the TF YBX1 negatively regulates proliferation-related gene Sp1 and positively regulates DNA repair-related genes *SHOX2* and *ERCC1*.

Besides, in core signaling pathways between normal stage and early stage of LSCC, we identified miRNAs, including miR-24-2 and miR-1247, and lncRNA, TUG1 (Figure 2). MiR-24-2 inhibits *SP1* in normal stage and has a higher expression in normal stage (*p*-value < 1.7 × 10^–27^). MiR-1247 inhibits *SP1* and *TERT* in normal stage and has a higher expression in normal stage (*p*-value < 6.7 × 10^–21^). TUG1 positively regulates gene *IGF2BP3* in the normal stage and has a higher expression in early stage of LSCC (*p*-value < 2.4 × 10^–5^). Moreover, there are seven genes, *SP1*, *SHOX2*, *ERCC1*, *SUSD4*, *TUG1*, *IGF2BP3*, and *KLF12*, with basal level difference which are than a threshold between normal stage and early stage LSCC, suggesting that this might have been caused by DNA methylation on the corresponding genes. Our results indicate that in the normal stage of lung cells which are adjacent to the LSCC, DNA repair is caused by the mediation of gene *ERCC1* and proliferation is inhibited by the mediation of genes, *KLF12* and *IGF2BP3*. However, in the early stage of LSCC, telomere maintenance and proliferation are caused by the mediation of gene *TERT* and mediation of genes, *SP1*, *SHOX2*, *TUG1*, and *IGF2BP3*, respectively. DNA repair and innate immune response are inhibited by the mediation of genes, *SHOX2* and *ERCC1*, and mediation of gene *SUSD4*, respectively.

### Analysis of core pathways to investigate different genetic and epigenetic progression mechanisms of LADC and LSCC from early stage to middle stage

As shown in Figure 3, in the early stage of LADC, ligand JAG2 binds to receptor NOTCH1, the activated NOTCH1 then triggers the downstream proteins, MDM4, which is affected by UBE2U-induced ubiquitination, SLC6A20, and CDC42EP1 to modulate TFs, ETS1 and ZEB1. The TF ETS1 affected by NHLRC1-induced ubiquitination negatively regulates gene *miR-27b*, involved in angiogenesis, epithelial-mesenchymal transition (EMT) and cell migration, and positively regulates EMT-related genes, *ZEB1* and *LOX*, which is also involved in extracellular matrix (ECM) remodeling, and migration-related gene *MYH9*. Besides, the receptor MET, which is affected by MIB2-induced ubiquitination and HDAC9-induced deacetylation, interacts with its ligand HGF, which is affected by NEURL4-induced ubiquitination and NAT14-induced acetylation, to modulate TF ZEB1. TF ZEB1 then positively regulates genes, *MYH9* and *miR27b*. Moreover, the ligand LTA binds to TNFRSFA, and TNFRSFA then directly triggers the TF Sp1 and indirectly triggers the TF HIF1A, which is affected by VHL-induced ubiquitination, through protein MUC1 to negatively regulate migration-related gene *MYH9* and to positively regulate angiogenesis-related gene *VEGFA* and ECM remodeling-related gene *LOX*.

In the middle stage of LADC, after interacting with HP, NOTCH1 interacts with Gamma-Secretase subunit APH1A to trigger the downstream protein SLC6A20 to modulate TF HIF1A. TF HIF1α then positively regulates angiogenesis-related gene *VEGFA*, ECM remodeling-related and EMT-related gene *LOX*. After receiving IL8 signaling, ITGA4 then modulates TF ETS1, which is affected by ATAT1-induced acetylation, and TF PRDM14 through the mediation of proteins VIM, which is affected by KAT7-induced acetylation, COL22A1, and MYH9, which is affected by NAT6-induced acetylation. TF ETS1 affected by ATAT1-induced acetylation then negatively regulates migration-related gene *miR-27b* and positively regulates EMT-related and ECM remodeling genes, *JAG2*, *ZEB1*, and *LOX*. TF PRDM14 positively regulates migration-related gene *CDC42EP1* and anti-apoptosis-related gene *IGF2BP1*. Besides, receptor MET is activated by binding to its ligand HGF to trigger TFs, ETS1, ZEB1, and HIF1A through the mediation by proteins, SLC5A2, CDKN2B, MDM4, APH1A, and SLC6A20. TF ETS1 negatively regulates anti-apoptosis-related genes *IGF2BP1* and *MALAT1*. TF ZEB1 negatively regulates migration-related genes *MYH9* and *JAG2*. TF HIF1A positively regulates angiogenesis-related genes *VEGFA* and *LOX*, which are also involved in EMT and ECM remodeling. In addition, after ligand LTA binding to receptor TNFRSFA, the activated TNFRSFA triggers the TF Sp1 to positively regulate anti-apoptosis-related gene *IGF2BP1* and EMT-related gene *ZEB1* through protein MUC1.

Besides, in core signaling pathways between early stage and middle stage of LADC, we identified miRNAs, including miR-30c2 and miR-27b, lncRNA, and MALAT1 (Figure 3). MiR-30c2 inhibits *JAG2* in both early stage and middle stage and has a higher expression in early stage compared to middle stage LADC (*p*-value < 4.7 × 10^–3^). MiR-27b inhibits *MYH9* in early stage and has a higher expression in early stage compared to middle stage LADC (*p*-value < 2.7 × 10^–2^). MALAT1 negatively regulates gene *IGF2BP1* in middle stage and has a higher expression in early stage LADC (*p*-value < 6.3 × 10^–3^). Moreover, there are two genes, *MYH9* and *IGF2BP1*, with basal level differences which are higher than a threshold between early and middle stage of LADC, suggesting that this might have been caused by DNA methylation on the corresponding genes. Our results indicate that in the early stage of LADC, apoptosis is caused by the mediation of gene *miR-27b*. Angiogenesis, cell migration, ECM remodeling, and EMT are inhibited by the mediation of genes, *miR-27b* and *VEGFA*, mediation of genes, *miR-27b*, *MYH9*, and *CDC42EP1*, mediation of gene *LOX*, and mediation of genes, *ZEB1* and *LOX*, respectively. However, in the middle stage of LADC, angiogenesis, cell migration, EMT, and ECM remodeling are caused by the mediation of genes, *miR-27b* and *VEGFA*, mediation of genes, *JAG2*, *miR-27b*, *MYH9*, and *CDC42EP1*, mediation of genes, *ZEB1* and *LOX*, and mediation of gene *LOX*, respectively. Apoptosis is inhibited by mediation of genes, *miR-27b*, *MALAT1*, *JAG2*, and *IGF2BP1*.

In the early stage of LSCC, the tensin TNS1 interacts with collagen COL1A1 to trigger the TFs, ETS1, GATA2, and JUN through the mediation of proteins, ACTA2, which is affected by CREBBP-induced acetylation and CDKN2A, which is affected by HDAC4-induced deacetylation (Figure 3). TF ETS1, which is affected by UBE2Q1-induced ubiquitination, negatively regulates proliferation-related genes *miR-106b* and *miR-21* and positively regulates proliferation-related gene *IGF1*. TF GATA2 positively regulates proliferation-related genes *miR-106b* and *miR-21*. TF JUN positively regulates *miR-21*. Besides, TF JUN is also modulated by receptors, NOTCH1 CCR7, and IGF1R, which are activated respectively by the binding of ligands, JAG1, CCL21, and IGF1, to positively regulate proliferation gene *miR-21*. The activated NOTCH1 can also directly trigger TF HIF1A, which is affected by UBE2Z-induced ubiquitination and HDAC5-induced deacetylation, to negatively regulate proliferation-related gene *JUN*.

In the middle stage of LSCC, TNS1 interacts with COL1A1 to trigger TF GATA2 by the mediation of proteins, ACTA2, CDKN2A affected by HDAC1-induced deacetylation, and PCDP1, to positively regulate cell migration-related genes, *TNS1* and *MYLK*, and proliferation-related gene *miR-21*, and to negatively regulate proliferation-related gene *miR-106b*. Through binding to ligand CCL21, receptor CCR7 triggers TF JUN to positively regulate proliferation-related gene *miR-21*. In addition, the activated IGF-1R binds to its ligand IGF-1, which is affected by EP300-induced acetylation to trigger the TFs, JUN, ETS1 affected by NAT9-induced acetylation, and JUNB, through the mediation of proteins, SUSD4, CDKN2A, PCDP1, TRAF4, MYLK, TSC2, PARG, and MMP2 in the signaling pathways. TF JunB positively regulates gene *MMP2* involved in extracellular matrix (ECM) degradation and both TFs, JUN and ETS1, which positively regulate proliferation-related genes, *miR-21* and *IGF1*, and to negatively regulate proliferation-related gene *JUN*. Moreover, after receiving the signaling from chemokine CCL13, the activated ITGB1 triggers TFs, JUN, and ETS1 to positively regulate cell migration-related genes *ITGB1* and *CCR7*.

Besides, in core signaling pathways between early stage and middle stage of LSCC, we identified miRNAs, including miR-21, miR-155, and miR-106b (Figure 3). MiR-21 inhibits *TNS1* and *CCR7* in early stage and has higher expression in the middle stage when compared to the early stage of LSCC. MiR-155 inhibits *MYLK* in the early stage of LSCC and has higher expression in the early stage when compared to the middle stage of LSCC. MiR-106b inhibits *MMP2* in the early stage of LSCC and has a higher expression in the early stage when compared to the middle stage of LSCC (*p*-value < 8.1 × 10^–3^). Moreover, there are five genes, *TNS1*, *miR-21*, *JUN*, *ITGB1*, *MMP2*, with basal level differences which are higher than a threshold between the early and middle stage of LSCC, suggesting that this might have been caused by DNA methylation on the corresponding genes. Our results indicate that in the early stage of LSCC, proliferation is caused by the mediation of genes, *miR-106*, *IGF1*, *miR-21*, and *JUN*. However, in the middle stage of LSCC, proliferation, cell migration, ECM degradation, and lymphangiogenesis all are caused by the mediation of genes, *miR-106b*, *IGF1*, *miR-21*, and *JUN*, mediation of genes, *TNS1*, *MYLK*, *ITGB1*, and *CCR7*, mediation of gene *MMP2*, and mediation of gene *IGF1*, respectively.

### Analysis of core pathways to investigate different genetic and epigenetic progression mechanisms of LADC and LSCC from middle stage to advanced stage

As shown in Figure 4, in the middle stage of LADC, receptor EGFR is activated by interacting with ligands EGF and BGN to trigger downstream protein PCK1, which is affected by UBE2D3-induced ubiquitination to modulate TF AR to positively regulate proliferation-related gene *EIF4B*. Besides, the activated EGFR also modulates TF MYC to positively regulate *EIF4B* through the mediation of proteins, RHPN2, MTOR, and EIF4B in the corresponding signaling pathways. The protein CDH2 can also modulate TF MYC through the mediation of proteins, FN1, MTOR, EIF4B, and CDC42EP1 in the corresponding signaling pathways to regulate proliferation-related gene *EIF4B* positively.

In the advanced stage of LADC, receptor EGFR is activated by ligand BGN to modulate TF AR to negatively regulate gene *PLAU*, which is involved in extracellular proteolysis, and migration-related gene *miR-19a* and to modulate TF MYC to positively regulate migration-related gene *miR-19a* through the mediation of proteins LOXL2, TSGA10, and CDC42EP1 in the corresponding signaling pathways. After binding with the ligand EPO, the receptor EPOR then modulates the TF STAT3 to positively regulate proliferation-related gene *RHOB* and also modulate TF MYC to positively regulate *miR-19a* through the mediation of proteins, RHOB affected by SIRT1-induced deacetylation in middle stage, and XRCC4 in the corresponding signaling pathways. Besides, ITGB1 is activated by interacting with ligand BGN to modulate TF ZEB1, which is affected by ESCO1-induced acetylation, to positively regulate gene *PLAU*, gluconeogenesis-related gene *PCK1*, and migration-related gene *CDH2* through the mediation of proteins, IQGAP1, CNIH4, and RHPN2 in the corresponding signaling pathways. In addition, the activated ITGB1 and N-cadherin (CDH2) can both directly trigger TF MYC to regulate migration-related gene *miR-19a* positively.

Besides, in core signaling pathways between the middle stage and advanced stage of LADC, we identified miRNAs, including miR-143HG and miR-19a, and lncRNA, AFG3L1P (Figure 4). MiR143HG inhibits *PLAU* in middle stage and has a higher expression comparing to the advanced stage of LADC. MiR-19a inhibits *RHOB* in the advanced stage of LADC and has a higher expression comparing to the middle stage of LADC (*p*-value < 3.4 × 10^–2^). AFG3L1P negatively regulates *EIF4B* in both middle and advanced stage of LADC and has a higher expression in middle stage comparing to the advanced stage of LADC. Moreover, there is a gene *PCK1*, with basal level difference which is higher than a threshold between middle and advanced stage of LADC, suggesting that this might have been caused by DNA methylation on the corresponding gene. Our results indicate that in the middle stage of LADC, proliferation is caused by the mediation of gene *EIF4B*. However, in the advanced stage of LADC, proliferation is caused by the mediation of genes, *EIF4B* and *RHOB*; gluconeogenesis is caused by the mediation of gene *PCK1*; cell migration is caused by the mediation of genes, *miR-19a* and *CDH2*; extracellular proteolysis is caused by the mediation of gene *PLAU*.

In the middle stage of LSCC, the receptor EMR2, which is affected by the USP20-induced deubiquitination, is activated by interacting with DDX5 to modulate TF FOXL1, which is affected by the HDAC9-induced deacetylation to positively regulate gene NF1 involved in proliferation and cell mobility through the mediation of proteins CHD3 and AGR3 in the corresponding signaling pathways (Figure 4). After binding to ligand TGFB1, the receptor ERBB4 then modulates TF GATA3, which is affected by the SIAH1-induced ubiquitination and HDAC5-induced deacetylation, to positively regulate proliferation-related gene *AGR3* and gene *CDH3* involved in cell adhesion. Besides, the receptor ERBB4 can also modulate TF YBX1 to negatively regulate gene *PDPN*, which is involved in cell mobility through the mediation of proteins, CDC42, LIN7C, FN1, and NF1 in the corresponding signaling pathways.

In the advanced stage of LSCC, receptor ERBB4 activated by the binding to COL18A1, which interacts with FN1. Both receptor CDH3 affected by OTUD1-induced deubiquitination and the receptor ITGA8 have interactions with FN1 to modulate TFs, GATA3, YBX-1, and FOXO3 through the mediation of proteins, FN1, NF1, RHOXF2, and SMG1 in the corresponding signaling pathways. TF GATA3 then positively regulates proliferation-related gene *SMG1* and lymphangiogenesis-related gene *VEGFC* and negatively regulates proliferation-related gene *FOXO3* and gene *CDH3* involved in cell adhesion. TF YBX1 negatively regulates gene *PDPN*, which is involved in cell mobility. TF FOXO3 positively regulates gene *NF1*, which participates in proliferation and cell mobility. Moreover, the membrane glycoprotein PDPN, which is affected by SIRT1-induced deacetylation, can interact with ERBB4 to modulate TFs, FOXO3 and GATA3 to regulate genes, *NF1*, *FOXO3*, *SMG1*, *CDH3*, and *VEGFC*, through the mediation of proteins, CHIC2, MSN, TUBB3, and RHOXF2 in the corresponding signaling pathway.

Besides, in core signaling pathways between the middle and advanced stage of LSCC, we identified miRNAs, including miR-199b, miR-330, and miR-9-2 (Figure 4). MiR-199b inhibits *PDPN* in the middle stage and has a higher expression comparing to the advanced stage of LSCC. MiR-330 inhibits *CDH3* in middle stage and has a higher expression in advanced stage comparing to the middle stage of LSCC (*p*-value < 3.4 × 10^–2^). MiR-9-2 inhibits *FOXO3* in advanced stage and has a higher expression comparing to the middle stage of LSCC. Moreover, there are three genes, *FOXO3*, *PDPN*, and *CDH3*, with basal level differences which are higher than a threshold between middle stage and advanced stage LSCC, suggesting that this might have been caused by DNA methylation on the corresponding genes. Our results indicate that in the middle stage of LSCC, proliferation is caused by the mediation of genes, *AGR3* and *NF1*. Cell adhesion and cell mobility are inhibited by the mediation of gene *CDH3* and mediation of genes, *NF1* and *PDPN*, respectively. However, in the advanced stage of LSCC, the proliferation is caused by the mediation of genes, *FOXO3*, *SMG1*, and *NF1*; the cell mobility is caused by the mediation of genes, *NF1* and *PDPN*; the cell adhesion is caused by the mediation of gene *CDH3*; the lymphangiogenesis is caused by the mediation of gene *VEGFC*.

### Comparing and summarizing the differential genetic and epigenetic progression mechanisms caused by the core signaling pathways within connective stages of LADC and LSCC

**Figure 5:**
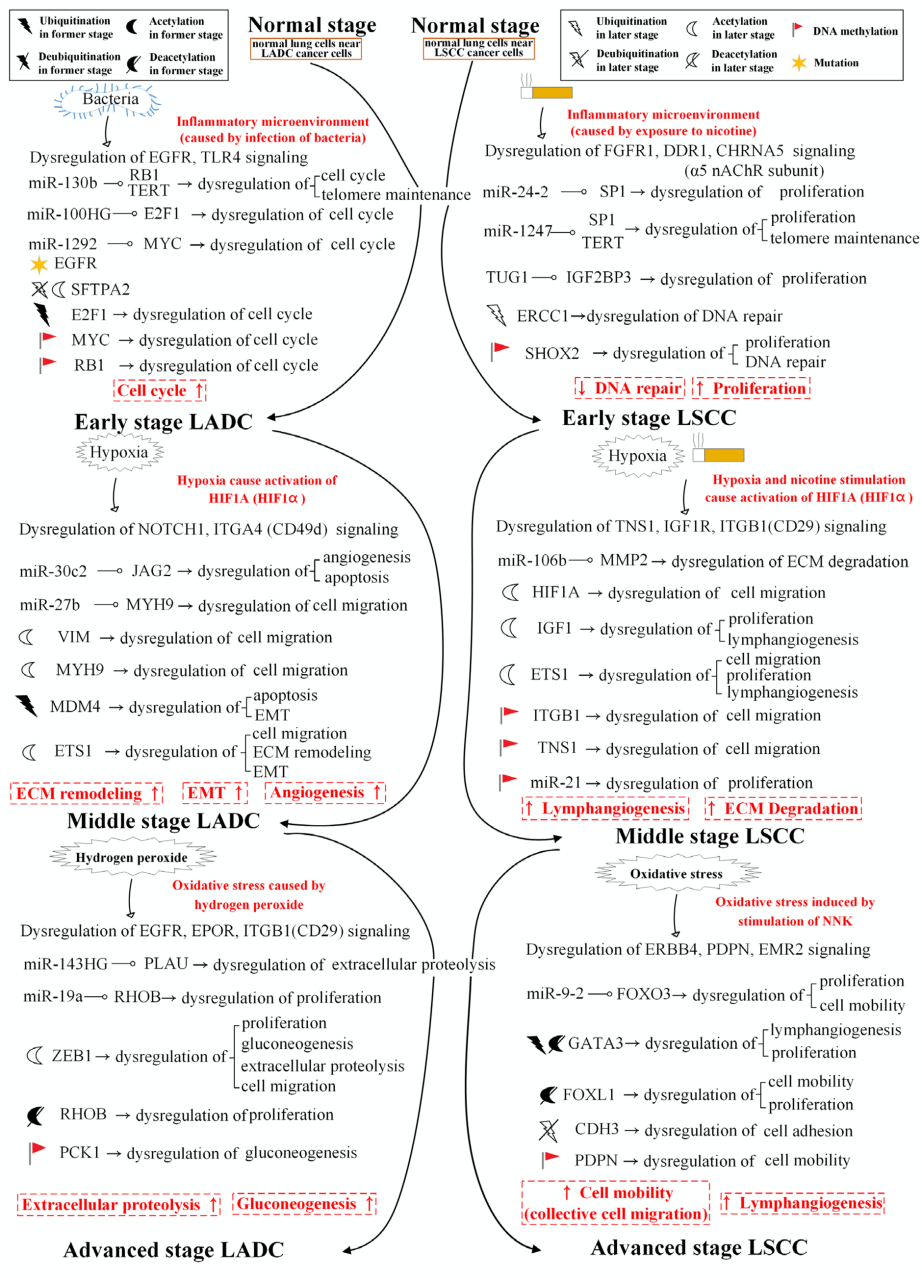
Summarizing the differential genetic and epigenetic progression mechanisms from normal stage to early stage, early stage to middle stage, and middle stage to advanced stage LADC and LSCC. The figure summarizes the differential genetic and epigenetic progression mechanisms caused by core signaling pathways within each connective stage of LADC and LSCC. The red font with dash-line rectangular blocks denote the differential function between LADC and LSCC.

In Figure 5, we summarize the differential genetic and epigenetic progression mechanisms caused by the core signaling pathways in connective stages which are from normal to early stage, early to middle stage, and middle to advanced stage of LADC and LSCC. The carcinogenic progression from normal to early stage of LADC results from abnormal cellular functions, such as telomere maintenance, cell cycle, circadian rhythm, immune response, and inflammatory response. These are caused by dysregulation of signaling pathways (PDGFRA, EGFR, TLR4, and RET signaling pathways), dysregulation of ubiquitination and acetylation, DNA methylation on genes (*RB1*, *CDK6*, *TGFB1*, *PER1*, and *MYC*), and abnormal miRNAs silencing (miR-130b, miR-100HG, miR-1292). While the carcinogenic progression from normal to early stage of LSCC results from abnormal cellular functions, such as telomere maintenance, DNA repair, proliferation, and innate immune response. These bring about by dysregulation of signaling pathways (FGFR1, DDR1, PDGFRB, and α5 nAChR subunit signaling pathways), dysregulation of ubiquitination and acetylation, DNA methylation on genes (*SP1*, *SHOX2*, *ERCC1*, *SUSD4*, *TUG1*, *IGF2BP3*, and *KLF12*), abnormal miRNAs silencing (miR-24-2, miR-1247) and lncRNA regulation (TUG1).

The carcinogenic progression from early to middle stage of LADC gives rise to abnormal cellular functions, such as angiogenesis, EMT, apoptosis, ECM remodeling, and cell migration. These are caused by dysregulation of signaling pathways (NOTCH1, MET, TNFRSF1A, and ITGA4 signaling pathways), dysregulation of ubiquitination and acetylation, DNA methylation on genes (*MYH9* and *IGF2BP1*), abnormal miRNAs silencing (miR-30c-2 and miR-27b) and lncRNA regulation (MALAT1). The carcinogenic progression from early to middle stage of LSCC results from abnormal cellular functions, such as proliferation, lymphangiogenesis, ECM degradation, and cell migration. These are caused by dysregulation of signaling pathways (TNS1, NOTCH1, CCR7, IGF1R, and ITGB1 signaling pathways), dysregulation of ubiquitination and acetylation, DNA methylation on genes (*TNS1*, *miR-21*, *JUN*, *ITGB1* and *MMP2*), and abnormal miRNAs silencing (miR-21, miR-21, and miR-106b).

The carcinogenic progression from middle to advanced stage of LADC contributes to abnormal cellular functions, such as proliferation, gluconeogenesis, cell migration, and extracellular proteolysis. These are caused by dysregulation of signaling pathways (EGFR, EPOR, ITGB1 and CDH2 signaling pathways), dysregulation of ubiquitination and acetylation, DNA methylation on gene (*PCK1*), abnormal miRNAs silencing (miR-143HG and miR-19a) and lncRNA regulation (AFG3L1P). In contrast, the carcinogenic progression from middle to advanced stage of LSCC contributes to abnormal cellular functions, such as proliferation, lymphangiogenesis, cell mobility, and cell adhesion. These are due to dysregulation of signaling pathways (ITGA8, PDPN, ERBB4, EMR2, and CDH3 signaling pathways), dysregulation of ubiquitination and acetylation, DNA methylation on genes (*FOXO3*, *PDPN*, and *CDH3*), and abnormal miRNAs silencing (miR-9-2, miR-199b, and miR-330).

**Figure 6:**
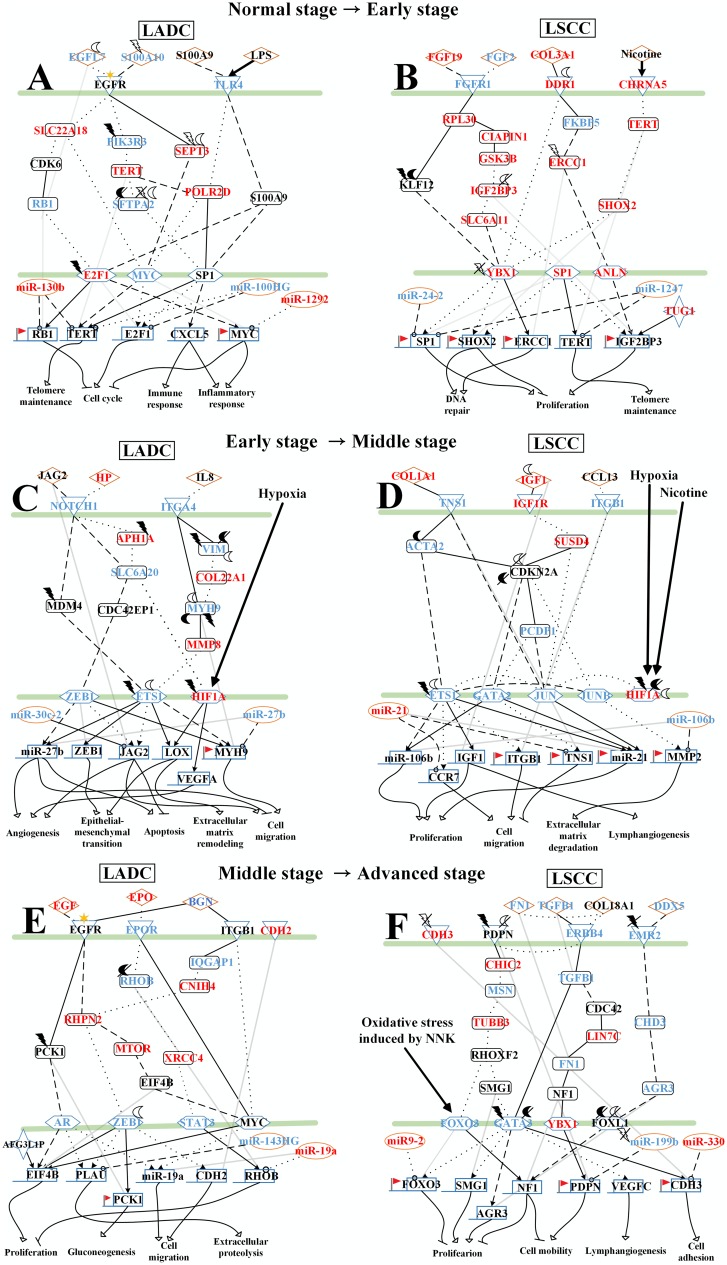
The specific core signaling pathways extracted from Figures 2–4 for investigating the differential progression molecular mechanisms between LADC and LSCC. (**A**) The genetic and epigenetic progression mechanisms from normal stage to early stage LADC could be potentially caused by inflammatory microenvironment induced by bacteria infection (LPS), dysfunctions of EGFR, and TLR4 signaling, regulation of miR-130b, miR-100HG, and miR-1292, epigenetic modifications of E2F1 and SFTPA2, DNA methylation of *MYC* and *RB1*, and aberrant cellular functions, such as cell cycle. (**B**) The genetic and epigenetic progression mechanisms from normal stage to early stage LSCC can be potentially caused by inflammatory microenvironment induced by exposure to xenobiotic toxicity nicotine, dysfunctions of FGFR1, DDR1, and CHRNA5 signaling, regulation of miR-24-2 and miR-1247, lncRNA TUG1, epigenetic modification of KLF12 and ERCC1, DNA methylation of *SHOX2*, and aberrant cellular functions, such as DNA repair. (**C**) The genetic and epigenetic progression mechanisms from early stage LADC to middle stage LADC can be potentially induced by hypoxic tumor microenvironment, dysfunctions of NOTCH1 and ITGA4 (CD49d) signaling, regulation of miR-30c-2 and miR-27b, epigenetic modifications of VIM, MYH9, MDM4, and ETS1, and dysregulation of cellular functions, such as angiogenesis, ECM remodeling, and EMT. (**D**) The genetic and epigenetic progression mechanisms from early stage LADC to middle stage LADC can be potentially induced by hypoxic tumor microenvironment exposed to nicotine, dysfunctions of IGF-1R, ITGB1 (CD29), and TNS1 signaling, regulation of miR-106b, epigenetic modifications of HIF1α, IGF-1, and ETS1, DNA methylation of *ITGB1*, *TNS1*, and *miR-21*, and dysregulation of cellular functions, such as lymphangiogenesis, cell migration, ECM degradation. (**E**) The genetic and epigenetic progression mechanisms from early stage LADC to middle stage LADC can be potentially induced by the alteration of tumor microenvironment caused by hydrogen peroxide secreted by cancer cells, dysfunctions of EGFR, EPOR, and ITGB1 (CD29) signaling, regulation of miR-143HG and miR-19a, epigenetic modifications of ZEB1 and RHOB, and DNA methylation of *PCK1*, and dysregulation of cellular functions, such as gluconeogenesis, extracellular proteolysis. (**F**) The genetic and epigenetic progression mechanisms from early stage LSCC to middle stage LSCC can be potentially induced by the alteration of tumor microenvironment caused by oxidative stress induced by the stimulation of nicotine derived nitrosaminoketone (NNK), dysfunctions of ERBB4, PDPN, and EMR2 signaling, regulation of miR-9-2, epigenetic modifications of GATA3, FOXL1, and CDH3, DNA methylation of *PDPN,* and dysregulation of cellular functions, such as proliferation, cell mobility, and cell adhesion.

To further investigate the impacts of miRNA regulations, DNA methylation, and epigenetic modifications on the genetic and epigenetic progression mechanisms between connective stages of LADC and LSCC, we extracted the specific core signaling pathways (Figure 6) out from these identified core signaling pathways (Figures 2–4) in each progression stage of LADC and LSCC. The more detailed comparison of genetic and epigenetic progression mechanisms will be discussed in the following sections.

## DISCUSSION

### Microenvironment change, dysregulation of miRNA/lncRNA regulation, DNA methylation, and epigenetic modification contribute to different genetic and epigenetic mechanisms from normal stage progresses to early stage of LADC and LSCC

As shown in Figure 6A and 6B, we identified that the inflammatory microenvironmental factor induced by bacteria infection (LPS) can potentially cause normal cells to progress to the early stage of LADC. Similarly, the inflammatory microenvironment induced by exposure to xenobiotic toxicity nicotine can potentially cause normal cells to progress to the early stage of LSCC. In general, LADC, which is thought to origin from the bronchiolar or alveolar epithelium (Clara cells or type II pneumocytes), mainly arises from the peripherally located airways, whereas LSCC, which typically often originates from the bronchial epithelium of larger and more central airways (basal cells), mostly arises from central lung [5, 7–9, 39]. Due to the different microenvironments, normal cells, which were nearby LADC and LSCC, receive different altered external signaling stimulations from their environment, and thus allow normal lung cells to have the potential to progress to LADC and LSCC through different genetic and epigenetic mechanisms, respectively. In LADC, inflammatory microenvironment induced by bacteria infection (LPS) can potentially cause the dysregulation of EGFR and TLR4 signaling pathways. Besides, the mutation of receptor EGFR, ubiquitination of E2F1, deubiquitination and acetylation of SFTPA2, dysregulation of DNA methylation in *RB1* and *MYC*, and dysregulation of miR-130b, miR-100HG, and miR-1292 could also result in abnormal cellular functions, such as cell cycle, telomere maintenance, and inflammatory response, causing normal cells to progress to early stage of LADC. In LSCC, microenvironment exposed to xenobiotic toxicity nicotine can potentially cause the dysregulation of FGFR1, DDR1, and CHRNA5 (α5 nAChR subunit) signaling pathways. Moreover, the ubiquitination of ERCC1, dysregulation of DNA methylation in *SHOX2* and *TUG1*, dysregulation of miR-24-2 and miR-1247, and dysregulation of lncRNA TUG1 result in abnormal cellular functions such as DNA repair, proliferation, and telomere maintenance, causing normal cells to progress to early stage of LSCC.

Interestingly, the epidemiologic and clinical studies have indicated the strong relation between inflammation, chronic infection, and cancer [40–42]. Besides, it is not surprising that microbiome, including bacteria, can be found in lung since the inhalation of air is one of sources of microbial immigration [43]. Though immune response is activated during infection, our results indicate TGFβ1 is secreted by cancer cells in early stage LADC (Figure 2) and plays a role in immunosuppression in lung cancer microenvironment [44–47], suggesting the cancer cells of LADC can prevent cytotoxic attack from lymphocytes, which are recruited to tumor microenvironment through stimulation of inflammation, by secretion of TGFβ1 in cancer microenvironment. While it has been reported that LSCC is highly correlated with exposure to tobacco smoke [5, 8, 12], which can support our finding. Our results also found SUSD4, an inhibitor of the complement system, is activated in early stage LSCC (Figure 2). It has been suggested that cancer cells can be killed by complement. To protect cancer cells themselves from being attacked by complement, they express the soluble or membrane-bound complement inhibitors [48, 49], suggesting that cancer cells of LSCC can activate SUSD4 against complement attack in early stage.

### Dysfunctions of miRNA regulation, DNA methylation, epigenetic modification and microenvironment alteration contribute to the progression from normal stage to early stage LADC

#### The inflammatory microenvironment caused by the infection of bacteria can lead to dysregulation of EGFR and TLR4 signaling pathways to progress from normal stage to early stage LADC

As shown in Figure 6A, our results reveal that the inflammatory environment caused by the infection of bacteria and the dysregulation of genetic and epigenetic regulations could lead to the dysregulation of EGFR and TLR4 signaling pathways, resulting in abnormal cell cycle and telomere maintenance to cause normal cells to progress to early stage LADC.

In the normal stage of lung cells near LADC cancer cells, we found that TLR4 is activated by LPS and pro-inflammatory factor S100A9 to trigger the inflammation signaling to modulate TFs, E2F1 and Sp1, to regulate cell cycle through the mediation of cell cycle-related gene *RB1*, to cause inflammatory response and immune response through the mediation of inflammation-related genes *MYC* and *CXCL5*, and to lead to telomere maintenance by the mediation of telomere-related gene *TERT*. During the inflammatory response, the pro-inflammatory factor S100A10 may be secreted by the nearby alveolar macrophage. S100A10 then binds with the receptor EGFR to trigger the TF MYC modulated by SEPT3 to cause telomere maintenance by the mediation of telomere-related gene *TERT*. Lipopolysaccharide (LPS), a component of Gram-negative bacteria cell wall, is a strong inflammatory stimulus. TLR4, Toll-like receptor 4, is not only expressed in immune cells, but also in epithelial cells, and is an important receptor for the recognition and initiation of inflammation signals. It has been reported that LPS can also activate immune cells to trigger the production of pro-inflammatory cytokines and other mediators through TLR4 [50, 51], causing the formation of inflammatory environment. Besides, our results also showed that the protein SFTPA2, which is involved in the clearance of pathogens in response to lipopolysaccharide (LPS) [52], is activated by several epigenetic modifications in the early stage of LADC suggesting that tumor cells may affected by the pathogens. Moreover, we also observed that the ligand protein Haptoglobin (HP), which can be increased in lung cancer during infection and inflammation [53, 54], is activated in middle stage LADC. Based on these information, we suggested that cells may be affected by the infection of becteria during the progression from normal cells to early stage LADC. However, due to the inflammatory response caused by the infection of bacteria, the microenvironment is altered, leading to formation of inflammatory microenvironment. The microenvironment is altered in early stage LADC, potentially causing the dysregulation of EGFR and TLR4 signaling.

In early stage LADC, LPS can bind to the receptor TLR4 to trigger TFs MYC and Sp1 through transductance protein POLR2D, to regulate cell cycle through the mediation of cell cycle-related gene *E2F1*. Besides, protein EGFL7 might be secreted via nearby cancer cells [55] to activate receptor EGFR to trigger TFs, E2F1, c-Myc, and Sp1, which are mediated by proteins SLC22A18, CDK6, RB1, PIK3R3, TERT, SFTPA2 in the corresponding signaling pathways, to regulate cell cycle through mediation of cell cycle-related genes *RB1* and *E2F1*. However, our results showed that not only the altered EGFR and TLR4 signaling pathway but also the dysfunctions of miRNA regulation, DNA methylation, and epigenetic modification cause normal cells to progress to the early stage of LADC. The epigenetic dysfunctions of miRNA regulation, DNA methylation and epigenetic modifications of LADC are described as follows.

#### Dysregulations of miR-130b, miR-100HG, and miR-1292, epigenetic modifications of EGFL7, SFTPA2, and E2F1, DNA methylation of MYC and RB1, and the mutation of EGFR contribute to the progression from normal stage to early stage LADC

EGFL7, also known as VE-statin, is a secreted protein. This protein was specifically expressed by cancer cells in various human tumors and by endothelial cells in normal tissues. In embryo, *EGFL7* is mainly expressed by endothelial cells; however it is downregulated in adult and has a small amount of expression in lung [47]. A vitro study showed that the expression of leukocyte adhesion molecules can be inhibited by EGFL7 through endothelial cells [56]. By preventing lymphocyte adhesion, the entry of immune cells into tumor microenvironment is limited. Our results reveal that EGFL7 is not downregulated in early stage due to the ELP3-induced acetylation, suggesting that EGFL7 plays a role in tumors immune evasion in the early stage of LADC. Besides, EGFL7 can bind to receptor EGFR to trigger oncogenic signaling to mediate abnormal cellular functions, such as cell cycle.

Moreover, the mutation of EGFR may also cause the dysregulation of EGFR signaling to induce abnormal cell cycle, which may potentially cause normal cell to progress to the early stage of LADC. Epidermal Growth Factor Receptor (EGFR), a tyrosine kinase, is involved in survival, proliferation, and tumorigenesis. It has been implicated in the growth of several human cancers, including lung cancer. Besides, the overexpression of EGFR has been found in several epithelial malignancies, including lung cancer (approximately 40–80 % of NSCLC) [57]. The catalogue of somatic mutations in cancer (COSMIC) database shows that mutations in EGFR have been associated with NSCLC, including LADC (https://www.sanger.ac.uk/genetics/CGP/cosmic). Surfactant Protein A2 (SFTPA2) is also known as SP-A. Previous studies have shown that SP-A also plays a role in the regulation of cellular responses, including inflammatory responses, by the regulation of producing pro-inflammatory cytokines in response to lipopolysaccharide to the clearance of a variety of pathogens. For example, SP-A can inhibit NO production and LPS-induced cytokine *in vivo* [52]. In early stage LADC, SFTPA2 is activated by USP19-induced deubiquitination and KAT5-induced acetylation. The activated SFTPA2 not only can help signaling transduction in EGFR signaling but also can allow cancer cells to clean the pathogens through expressing Surfactant Protein A2 (SFTPA2) to survive in early stage LADC.

Due to the effect of HECW2-induced ubiquitination, TF E2F1 is degraded in normal cells, resulting in the inhibition of E2F1 in normal cells. Besides, E2F1 is also inhibited by a higher expression of miR-100HG in normal stage. This allows cell cycle-related gene *RB1* and inflammatory-related gene *MYC* to be transcribed to induce inflammatory response and inhibit cell cycle progression. However, the E2F1 expression is increased in early stage without the modification of ubiquitination, inducing cell cycle by the mediation of cell cycle-related gene *RB1*. RB1 is an important regulator of entry into cell division and can prevent excessive cell growth through the inhibition of cell cycle progression. It is known that RB1 is a tumor suppressor and is frequently inactivated in many cancers, including lung cancer. Our results showed that though *RB1* is upregulated by the regulation of degraded E2F1, *RB1* is inhibited by miR-130b in normal stage. Besides, with a lower basal level in the early stage, *RB1* has a significant basal level difference between normal and early stage of LADC reflecting the repressive ability on this gene might be caused by DNA methylation. According to comparing DNA methylation profiles of normal stage and early stage LADC, the significant change of methylation level in *RB1* (*p*-value < 1.2 × 10^–23^) can also support this result. Hence the abnormal cell cycle function caused by the dysregulation of RB1 can lead to the accumulation of DNA damage to potentially drive the normal cells to progress to LADC.

V-Myc Avian Myelocytomatosis Viral Oncogene Homolog (c-Myc) is the coding protein of gene *MYC* and involved in cell cycle progression and apoptosis. In early stage LADC, *MYC* is inhibited by the activated miR-1292, which may be involved in cell cycle (*p*-value < 1.2 × 10^–4^). Moreover, with a lower basal level in early stage, *MYC* has a significant basal level difference between normal and early stage of LADC reflecting that the lower expression is partly caused by the repression of DNA methylation. According to comparing DNA methylation profiles of normal stage and early stage LADC, the significant change of methylation level in *MYC* (*p*-value < 1.2 × 10^–18^) can also support this result. These indicate that MYC is inhibited in early stage LADC, leading to the upregulation of E2F1 to induce cell cycle. *TERT*, which encodes Telomerase Reverse Transcriptase, is a ribonucleoprotein enzyme and plays a role in the regulation of telomerase activity, telomere maintenance and cellular senescence. It has been reported that the aberrant expression of *TERT* has been found in lung cancer [58]. Our results show that *TERT* is inhibited by miR-130b in normal stage. However, *TERT* is upregulated by the TF Sp1 and inhibited by miR-100HG without the inhibition of miR-130b in early stage LADC, indicating that the dysfunction of miR-130b can cause an abnormal telomere maintenance to allow cells to become immortal to drive normal lung cells to progress to the early stage of LADC.

### Dysfunctions of miRNA regulation, DNA methylation, epigenetic modification and microenvironment alteration contribute to the progression from normal stage to early stage LADC

#### The inflammatory microenvironment caused by exposure to nicotine can lead to the dysregulation of FGFR1, DDR1, and CHRNA5 (α5 nAChR subunit) signaling pathways from normal stage to early stage of LSCC

As shown in Figure 6B, our results reveal that the inflammatory microenvironment caused by exposure to nicotine and the dysregulation of genetic and epigenetic regulation could lead to the dysregulation of FGFR1, DDR1, and CHRNA5 signaling pathways, resulting in abnormal DNA repair, proliferation, and telomere maintenance to cause normal cells to progress to early stage LSCC.

In the normal stage of lung cells near LSCC cancer cells, receptor FGFR1 activated by FGF19, which might be secreted by nearby cancer cells, triggers the repair signaling to affect TF YBX1 through cascade proteins, RPL30 and KLF12 to regulate DNA repair by the mediation of DNA repair-related gene *ERCC1*. FGF19 participates in a variety of cellular processes, such as tissue repair, cell growth, morphogenesis, and, tumor growth. Recent study indicated that LSCC cell growth can be stimulated by FGF19 [59]. Moreover, the collagen COL3A1 interacts with receptor DDR1 on normal cells to trigger TF ANLN, which is modulated by proteins, KFBP5 and ERCC1, to regulate proliferation through the proliferation-related gene *IGF2BP3*. However, due to the inflammatory response caused by exposure to nicotine, the microenvironment is altered, leading to the formation of inflammatory microenvironment. The altered microenvironment could potentially cause the dysregulation of FGFR1, DDR1, and CHRNA5 (α5 nAChR subunit) signaling pathways.

In early stage LSCC, CHRNA5, the nicotinic acetylcholine receptor subunit (α5 nAChR subunit), activated by nicotine, triggers the TF YBX1 through cascade proteins, TERT and SHOX2 to regulate DNA repair and proliferation through the mediation of gene *SHOX2* as shown in Figure 6B. Nicotine, a xenobiotic toxicity, is addictive substance in cigarette smoke. It has been reported that nicotine will cause oxidative stress, leading to DNA damage, and also plays a role in immunosuppressive [60–62]. Besides, nicotine can facilitate the degradation and reorganization of stromal matrix and the secretion of extracellular matrix proteins, contributing to the formation of cancer-promoting environment [60]. Collagen, the most abundant ECM protein in the matrix, can also contribute to tumor progression in the tumor matrix [63]. It has been reported that COL3A1 can promote cell proliferation, migration, and monocyte recruitment in cancer [64], suggesting that the monocyte was recruited to the microenvironment of LSCC by COL3A1 while environment was stimulated by nicotine. The receptor DDR1 is activated by collagen COL3A1 to directly trigger the TF YBX1 to regulate DNA repair by the mediation of DNA repair-related gene, *ERCC1* and to regulate proliferation by the mediation of proliferation-related gene, *SP1*. In addition, receptor FGFR1 activated by the ligand FGF2 which may be secreted via nearby fibroblast is involved in cancer growth and malignant progression of NSCLC [65]. Triggered TF Sp1 through a cascade of proteins, RPL30, CIAPIN1, GSK3B, IGF2BP3, SLC6A11 in the corresponding signaling pathways regulates telomere maintenance by the mediation of telomere-related gene *TERT*, and both proliferation and DNA repair by the mediation of *SHOX2* and *IGF2BP3*. However, our results show that not only the genetic dysregulations of FGFR1, DDR1, and α5 nAChR subunit signaling pathways affected by the altered microenvironment but also epigenetic dysfunctions of miRNA regulation, DNA methylation, and epigenetic modification caused normal cells to progress to the early stage of LSCC. The epigenetic dysfunctions of miRNA regulation, DNA methylation, and epigenetic modification of LSCC are described as follows.

#### Dysregulations of miR-24-2, miR-1247, epigenetic modifications of KLF12 and ERCC1, and DNA methylation of SHOX2 contribute to the progression from normal stage to early stage LSCC

Kruppel Like Factor 12 (KLF12) has been reported that its reduced expression can promote the cell cycle G1/S transition and make the cell more susceptible to undergo apoptosis after cell detachment from matrix in tumor, suggesting that the expression of KLF12 in normal cells can allow normal cells to growth properly [66]. Based on our results in Figure 6B, we found that KLF12 is inhibited by the KAT7-induced acetylation and UBE2J1-induced ubiquitination in normal stage. The inhibition of KLF12 not only can induce cell cycle G1/S transition, but also can lead to the dysregulation of FGFR1 pathway by interrupting the signaling transduction in normal stage, which may cause abnormal DNA repair to enhance the risk of carcinogenesis. Besides, after binding to collagen COL3A1, the acetylated receptor DDR1 enters the cytoplasm to directly interact with TF YBX1 to mediate DNA repair and proliferation. In early stage LSCC, the USP6-induced deubiquitination of YBX1 results in an activated YBX1 to regulate *ERCC1* and *SHOX2* to induce DNA repair. However, in spite of the effect of the change of DNA methylation, protein ERCC1 is degraded by the BTRC-induced ubiquitination, leading to the inhibition of DNA repair. Hence, the abnormal DNA repair may induce the accumulation of DNA damage caused by exposure to carcinogen nicotine, which may cause normal cells to progress to early stage LSCC. Y-Box Binding Protein 1 (YBX1), a cold shock domain protein, has been implicated in the regulation of various cellular functions, such as transcription and DNA repair.

Sp1, a zinc finger transcription factor of the Krüppel-like factor family, is expressed ubiquitously in various mammalian cells and is involved in the regulation of cellular processes, including cell growth, cell differentiation, immune responses, and DNA damage response [67]. Our results show that *SP1* is inhibited by the activated miR-24-2 and miR-1247 in normal stage. However, in the early stage of LSCC, *SP1* affected by DNA methylation (Figure 2) is upregulated by the regulation of ETS1 without inhibitions from miR-24-2 and miR-1247. This indicates that proliferation can be activated by the mediation of *SP1*, which could potentially cause normal cells to progress to early stage LSCC. Besides, it has been reported that the expression level of Sp1 is accumulated strongly higher in early stage [68], which is consistent with our result.

Moreover, our results also found that *TERT* is inhibited by the activated miR-1247 in normal stage, however, in early stage LSCC, *TERT* is upregulated by Sp1 in the aberrance of miR-1247 silencing, indicating that the activation of telomere maintenance through the mediation of *TERT* can allow normal cells to become immortal to potentially progress to early stage LSCC. In addition, in spite of the effect of the DNA methylation, *IGF2BP3* is upregulated by the positive regulation of the activated lncRNA TUG1, leading to the activation of proliferation in early stage LSCC. Moreover, lncRNA TUG1 is involved in the regulation of cancer progression. It has been reported that the downregulation of TUG1 can be observed in NSCLC [69]. However, our results showed that TUG1 plays a role in promoting LSCC proliferation. Some studies proposed that a downregulated TUG1 may potentially inhibit proliferation and cell invasion [70, 71], which can support our results. For *SHOX2* with a lower basal level in early stage, it has a significant basal level difference between normal and early stage LSCC reflecting that the repressive ability on this gene might be caused by DNA methylation. According to comparing DNA methylation profiles of early stage LSCC and middle stage LSCC, the significant change of methylation level in *SHOX2* (*p*-value < 6.4 × 10^–17^) supports our result. Based on what we mentioned above, abnormal DNA repair and proliferation may lead to the accumulation of DNA damage driving normal cells to progress to early stage LSCC.

#### Microenvironment change, dysregulation of miRNA/lncRNA, DNA methylation, and epigenetic modification contribute to genetic and epigenetic progression mechanisms from early stage to middle stage of LADC and LSCC

As shown in Figure 5C and 5D, we identified that a hypoxic tumor microenvironment can potentially cause early stage LADC to progress to middle stage LADC, while the hypoxic tumor microenvironment exposed to xenobiotic toxicity nicotine can potentially cause early stage LSCC to progress to middle stage LSCC. In LADC, a hypoxic tumor microenvironment can potentially cause the dysregulation of NOTCH1 and ITGA4 signaling pathways. Besides, the acetylation of VIM and MYH9, acetylation of ETS1, and dysregulation of miR-30c-2 and miR-27b could also result in abnormal cellular functions, such as angiogenesis, EMT, apoptosis, ECM, and cell migration, causing early stage LADC to progress to middle stage LADC. In LSCC, a hypoxic tumor microenvironment exposed to xenobiotic toxicity nicotine can potentially cause the dysregulation of IGF-1R, ITGB1 and TNS1 signaling pathways. Moreover, the acetylation of IGF-1, ACTA2, ETS1 and HIF1α, the DNA methylation in *ITGB1*, *TNS1*, *miR-21*, and the dysregulation of miR-106b and miR-21 result in abnormal cellular functions, such as proliferation, cell migration, ECM degradation, and lymphangiogenesis, causing early stage LSCC to progress to middle stage LSCC.

Interestingly, based on our results in LADC, we found that cellular functions of angiogenesis and EMT are activated during the progression from early stage to middle stage LADC. It indicates that LADC cancer cells may undergo EMT to increase migrating ability to potentially drive cancer cells to invade surrounding tissue by secreting ECM remodeling-associated protein LOX. EMT is a process inducing epithelial cells to acquire a mesenchymal phenotype. Recent study demonstrated that EMT has been observed in LADC and LSCC [72]. EMT can allow malignant epithelial cell to detach from the primary tumor to promote the ability of cancer migration. However, our results showed that EMT occurs in LADC rather than LSCC, indicating that LSCC cancer cells may promote their invasive ability by other way. Indeed, in LSCC, the ECM degradation is activated during the progression from early stage to middle stage LSCC, indicating that cancer cells of LSCC can secret ECM degradation-associated protein MMP-2 to promote their invasive ability. Moreover, angiogenesis is activated partly by the secretion of angiogenesis factor VEGFA during the progression from early stage to middle stage, enabling LADC cancer cells to gain more sufficient oxygen to prevent hypoxia-induced apoptosis and potentially promote the dissemination of LADC cancer cells through hematogenous metastasis. While in LSCC, lymphangiogenesis is activated by the mediation of IGF-1 during the progression from early stage to middle stage LSCC. We found that the upregulated CCR7 binding to ligand CCL21 secreted by lymph node allows cancer cells to have chemotaxis toward lymph node, indicating that cancer cells of LSCC may invade to lymph node and metastasize through lymphatic metastasis.

### Dysfunctions of miRNA regulation, DNA methylation, epigenetic modification and microenvironment alteration contribute to the progression from early stage to middle stage LADC

#### The hypoxic tumor microenvironment can lead to the dysregulation of NOTCH1 and CD49d signaling pathways from early stage to middle stage LADC

As shown in Figure 6C, our results reveal that the hypoxic tumor microenvironment and the genetic and epigenetic dysregulation could lead to the alteration of NOTCH1 and ITGA4 signaling pathways, resulting in abnormal angiogenesis, EMT, apoptosis, ECM remodeling, and cell migration to cause early stage LADC to progress to middle stage LADC.

In early stage LADC, NOTCH1 is activated by the interaction with ligand Jagged 2 (JAG2) secreted by the nearby cancer cells and triggers TF ETS1 mediated by protein MDM4 and TF ZEB1 mediated by proteins, SLC6A20 and CDC42EP1 in the corresponding signaling pathways to regulate cell migration through the mediation of cell migration-related genes, *MYH9* and *miR-27b*, to regulate ECM remodeling through the mediation of gene *LOX*, to regulate EMT through the mediation of *LOX* and *ZEB1*, and to regulate angiogenesis and apoptosis through the mediation of gene *miR-27b*. Recent studies have reported that NOTCH1 is implicated in cancer development and progression in NSCLC and is involved in tumor initiation, growth, EMT, and metastasis [73–79]. In addition, it has been observed that NOTCH1 could suppress tumor proliferation under normoxia, while under hypoxia, NOTCH1 is involved in the promotion of tumor [80]. However, the rapid growth of cancer cells in early stage may extend beyond the oxygen diffusion limit, thus the tumor of LADC and surrounding cells in microenvironment are subjected to a low oxygen condition, causing a hypoxic tumor microenvironment. The alteration of microenvironment may cause the dysregulation of NOTCH1 and ITGA4 signaling pathways.

In middle stage LADC, HIF1A is expressed by the stimulation of hypoxia, and NOTCH1 activated by interacting with Haptoglobin, which may be secreted by the nearby cancer cells, also triggers TF HIF1α to regulate angiogenesis by the mediation of angiogenesis-related gene *VEGFA* and to regulate ECM remodeling and EMT by the mediation of gene *LOX* (Figure 6C). Haptoglobin (HP), an acute phase plasma glycoprotein, can bind to free hemoglobin to prevent oxidative stress. HP is generated from the liver. In blood plasma, the increased amount of HP has been found in infection, inflammation, and various malignant diseases, including lung cancer [53, 54]. Besides, it is revealed that the overexpression of HIF1α is associated with the upregulated HP under the hypoxia condition in human hepatoma cells [81], suggesting that HP may be secreted by nearby cancer cells under the hypoxia. In addition, the ITGA4 activated by chemokine IL-8, which is secreted by the surrounding macrophage stimulated by hypoxia, triggers TF ETS1 modulated by proteins, Vimentin (VIM), COL22A1, MYH9, and MMP-8 in the corresponding signaling pathways, to regulate cell migration through the mediation of cell migration-related genes, *miR-27b* and *JAG2*, to regulate ECM remodeling through the mediation of gene *LOX*, to regulate EMT through the mediation of genes, *LOX* and *ZEB1*, and to regulate apoptosis through the mediation of genes, *JAG2* and *miR-27b*. However, our results show that not only the dysregulation of NOTCH1 and ITGA4 signaling pathways, which are affected by the alteration of microenvironment, may be involved in the progression from early stage of LADC to progress to middle stage of LADC, but also the dysregulation of miRNA, DNA methylation, and epigenetic modification can cause early stage LADC to progress to middle stage LADC. The epigenetic dysfunctions of miRNA regulation, DNA methylation, epigenetic modification of LADC are described as follows.

#### Dysregulations of miR-30c-2 and miR-27b, epigenetic modifications of VIM, MYH9, MDM4, and ETS1 contribute to the progression from early stage to middle stage LADC

MDM4, also known as MDMX, is an important player in cancer progression. MDMX silencing can result in apoptosis and growth arrest in NSCLC cells [82]. Our results in Figure 6C show that the protein MDM4 is modified by the UBE2U-induced ubiquitination in early stage. MDMX can form heteroligomers with MDM2, an E3 ubiquitin ligase, to increase the MDM2 ubiquitin ligase activity to suppress the transcriptional activity of p53 by binding to p53 and mediate the proteasomal degradation of cell cycle-related proteins [83, 84]. Besides, DNA damage can induce the degradation of MDMX [85]. It has been also reported that EMT phenotype is associated with an increased expression of MDMX [86]. Hence we suggest that the ubiquitination of MDM4 may lead to the growth arrest of LADC, allowing cancer cells to maintain in early stage, not to progress to middle stage LADC. However, the ubiquitination of MDM4 may influence on the NOTCH1 signaling pathway to cause the dysregulation of downstream signalings and functions, such as apoptosis, angiogenesis, EMT, ECM remodeling and cell migration, which may potentially drive early stage LADC to progress to middle stage LADC.

Our results also show that ETS1 is degraded by the NHLRC1-induced ubiquitination in early stage to allow cancer cells to maintain in early stage by inhibiting invasive functions, such as cell migration and ECM remodeling. However, the ATAT1-induced acetylation of ETS1 could enhance its transcription ability in middle stage to activate cell migration, ECM remodeling and EMT through the mediation of *JAG2* and *miR-27b,* the mediation of *LOX,* the mediation of *ZEB1* and *LOX*, respectively to potentially cause early stage LADC to progress to middle stage LADC. Zinc finger E-box binding homeobox 1 (ZEB1) is a transcription factor involved in EMT. It has been shown that the expression of ZEB1 was correlated with the mesenchymal phenotype in NSCLC and significantly correlated with the expression of *VIM* [87, 88]. Besides, we observed that *JAG2* is inhibited by miR-30c-2 in both early and middle stage of LADC. However, the inhibited miR-30c-2 can cause the upregulation of *JAG2* in middle stage to induce angiogenesis and anti-apoptosis. It has been reported that in mice, JAG2 is associated with the promotion of metastasis in lung adenocarcinoma [77]. Besides, the increased expression of JAG2 is associated with hypoxia and the increased expression of JAG2 on tumor cells can promote the tube formation of endothelial cell [89], indicating the involvement of JAG2 in angiogenesis. These indicates that JAG2 is increased in middle stage to promote the angiogenesis and invasion ability of cancer cells.

Besides, our results in Figure 6C show that HIF1A is degraded by VHL-induced ubiquitination in the early stage of LADC. However, when microenvironment becomes hypoxic, HIF1A is activated by the stimulation of hypoxia, hence transcribing genes, *VEGF* and *LOX* induce angiogenesis, ECM remodeling, and EMT. Hypoxia Inducible Factor 1 Alpha Subunit (HIF1α) is an important mediator of the cellular response to hypoxia and is involved in the cellular regulations of cancer cells, including angiogenesis, erythropoiesis, invasion, and metastasis [90–92]. Under normoxia, HIF1α can be degraded by ubiquitin protein VHL; however, it can be stabilized under hypoxia. It has been reported that the expression of HIF1A is increased in NSCLC and the expression of HIF1α is significantly associated with VEGFA [93–96]. These are consistent with our results, which can support our findings. LOX is an enzyme and can catalyze the cross-linking of ECM components, such as collagen and elastin, by oxidizing lysine residues. It has been reported that HIF1α can regulate LOX to promote LOX transcription under hypoxia, and LOX can increase EMT, cell invasion, and motility, and enhance metastasis *in vivo* [97, 98]. The increased expression of LOX has been observed in tissue sample from patients with LADC, and the co-expression of LOX and HIF1α is associated with the decreased survival of NSCLC patients [98, 99].

Owning to the effect of modifications of VHL-induced ubiquitination and SIRT5 -induced deacetylation, VIM is decreased in early stage LADC, while VIM is increased by the modification of KAT7-induced acetylation. Vimentin (VIM), a major constituent of proteins belonging to the intermediate filament family, can provide resistance against stress and is involved in maintaining cellular integrity. VIM is a marker of the EMT [100, 101]. During EMT, VIM can induce changes in cell motility and shape to participate in cell migration [100], suggesting that the activated VIM caused by acetylation can promote cell migration in middle stage LADC.

Myosin-9 (MYH9) is an actin-binding motor protein. In our results, MYH9 is decreased in early stage LADC and caused by UBE2U-induced ubiquitination, ESCO1-induced acetylation, and the inhibition of miR-27b, while the NAT6-induced acetylation of MYH9 leads to the activation of MYH9 without the inhibition of miR-27b in middle stage LADC, though MYH9 is also affected by DNA methylation. It has been observed that the dysregulation of miR-27b has been observed in human cancers, for example, the downregulated miR-27b can be found in lung adenocarcinoma [102, 103]. MiR-27b could inhibit the invasion, migration, and proliferation in cancer cells [104]. Besides, it has been shown that miR-27b can increase the vascularization and growth of subcutaneous tumors significantly, for example, miR-27b can promote the angiogenesis and cancer growth in lung cancer [105]. Recently studies have shown that MYH9 participates in cancer cell migration, invasion, and metastasis, and MYH9 expression was significantly correlated with the adenocarcinoma histology [106–108], suggesting that the activation of MYH9 (*p*-value < 4.4 × 10^–2^) caused by acetylation can promote cell migration in middle stage LADC and may potentially increase LADC invasion and metastasis.

### Dysfunctions of miRNA regulation, DNA methylation, epigenetic modification and microenvironment alteration contribute to the progression from early stage to middle stage LSCC

#### The hypoxic tumor microenvironment exposed to nicotine can lead to the dysregulation of IGF-1R, TNS1, and ITGB1 (CD29) signaling pathways from early stage to middle stage LSCC

As shown in Figure 6D, our results reveal that the hypoxic tumor microenvironment exposed to xenobiotic toxicity nicotine and the dysfunction of genetic and epigenetic regulation could lead to dysregulation of IGF-1R and ITGB1 signaling pathway and the dysregulation of focal adhesion molecule TNS1 signaling, resulting in abnormal proliferation, ECM degradation, cell migration, and lymphangiogenesis to cause early stage LSCC to progress to middle stage LSCC.

In early stage, the focal adhesion molecule tensin 1 (TNS1) interacts with the extracellular matrix protein COL1A1, which is a collagen to trigger the TFs JUN, GATA2, and ETS1 via cascade proteins ACTA2, CDKN2A, and PCDP1 in the corresponding signaling pathway and to regulate both the proliferation through the mediation of proliferation-related genes *miR-21*, *miR-106b*, and *IGF1* and the lymphangiogenesis through the mediation of gene *IGF1*. In previous microarray analyses, the upregulated COL1A1 was found in various malignant tissues, including lung cancer [109]. Besides, the overexpression of COL1A1 is correlated with hypoxic conditions and the strong correlation has been observed between its expression and hypoxia markers [110]. CDKN2A expressed in the cytoplasm and nucleus can form a complex with other proteins such as actin and tubulin depending on post-translational modifications [111, 112], which can support our results; the deacetylated CDKN2A can interact with the acetylated ACTA2 to promote cell migration. However, similar to the NSCLC subtype LADC, the rapid cell proliferation could lead to cancer cells in early stage to extend beyond the oxygen diffusion limit. Therefore, the LSCC cancer cells and cells surrounding in microenvironment are subject to a low oxygen condition, causing a hypoxic tumor microenvironment. Besides, a hypoxic tumor microenvironment is also exposed to xenobiotic toxicity nicotine. The alteration of microenvironment could cause the dysregulation of TNS1, IGF-1R and CD29 signaling pathways.

In middle stage LSCC, HIF1A is activated. It has been shown that hypoxia-inducible factor 1α (HIF1α) can be activated by the stimulation of hypoxia and also by the stimulation of nicotine in lung cancer [113], suggesting HIF1α is expressed by the stimulation of both hypoxia and xenobiotic toxicity nicotine in middle stage LSCC. The activated HIF1α then binds with TFs, JUN and ETS1, to regulate proliferation and lymphangiogenesis by the mediation of proliferation-related gene *miR21* and *IGF1*, which is also involved in lymphangiogenesis. After IGF-1 binding to receptor IGF-1R secreting caused by the stimulation of hypoxia, it triggers TFs JUN, ETS1, JunB, and GATA2 through cascade proteins SUSD4, CDKN2A, and PCDP1 in the corresponding signaling pathways to regulate ECM degradation through the mediation of *MMP2*, proliferation through mediation of *miR-21*, *miR-106*, and *IGF1*, lymphangiogenesis through the mediation of *IGF1*, and cell migration through the mediation of *TNS1* (Figure 6D).

In addition, when CCL13 binding to ITGB1, CD29 then directly triggers the TF JUN. TF c-Jun then interacts with ETS1 to regulate cell migration by the mediation of *CCR7*, and *ITGB1*. It has been reported that the induction of senescence in cells can cause an increased expression of chemokines, such as CCL13 [114]. Besides, bronchial epithelial cell can induce apoptosis and senescence through ROS-mediated autophagy-impairment by exposure to nicotine. Hence we suggest that CCL13 may be secreted by nearby bronchial epithelial cells, which are induced by senescence under nicotine exposure. ETS1, Ets1 proto-oncoprotein, is a member of the Ets family of transcription factors and is involved in the regulation of several cancer-associated functions, such as ECM remodeling, cell migration, and proliferation [115, 116]. It has been reported that ETS1 can enhance transcription synergistically with AP-1 (c-Jun) by interacting with AP-1 (c-Jun) to form complex [117], which can support our results; TF JUN can interact with ETS1 to regulate cell migration by the mediation of *CCR7* and *ITGB1*. However, our results show that not only the dysregulation of TNS1, IGF-1R and ITGB1 signaling pathways, which are affected by the altered microenvironment, may be involved in the progression from early stage to middle stage LSCC, but also the dysfunctions of miRNA regulation, DNA methylation, and epigenetic modification can cause early stage LSCC to progress to middle stage LSCC. The epigenetic dysfunctions of miRNA regulation, DNA methylation, epigenetic modification of LSCC are described as follows.

#### Dysregulation of miR106b, epigenetic modifications of HIF1α, IGF-1, and ETS1, and DNA methylation of ITGB1, TNS1, and miR21 contribute to the progression from early stage to middle stage LSCC

In early stage LSCC, the UBE2Q1-induced ubiquitination of ETS1 leads to the downregulation of *IGF1*, resulting in the inhibition of lymphangiogenesis as shown in Figure 6D. However, the NAT9-induced acetylation of ETS1 in middle stage LSCC leads to the upregulation of cell migration-related genes, *CCR7* and *ITGB1*, and lymphangiogenesis-related gene *IGF1*, resulting in the activation of cell migration and lymphangiogenesis. The activation of cell migration and lymphangiogenesis may promote the invasion ability of cancer cells and potentially allow cancer cells to undergo lymphatic metastasis in the middle stage of LSCC.

IGF-1R, a transmembrane heterotetrameric protein, can promote cancer cell proliferation, survival and transformation toward malignancy. IGF-1, an insulin-like growth factor, can be delivered not only from distant sources, but also through paracrine/autocrine signalings in aggressive tumors [118]. Through receiving the acetylated IGF-1 signaling, IGF-1R is activated to regulate cancer-associated functions, such as the proliferation to promote cancer cell proliferation. It has been shown that IGF1R can be stimulated by its ligand insulin-like growth factor 1 (IGF-1) [119]. The aberrant IGF signaling has been observed in several cancers, including lung cancer [120]. Besides, the increased IGF-1R activity is implicated in cancer cell function such as cell migration, proliferation, and invasion [118, 120]. In our study, we found that HIF1α is inhibited by UBE2Z-induced ubiquitination and HDAC5-induced deacetylation. However, in middle stage LSCC, HIF1A is activated by the stimulation of hypoxia and nicotine [113]. In addition, HIF1α is also affected by NAT8-induced acetylation. The activated HIF1α then interacts with both JUN and ETS1, which are also affected by NAT9-induced acetylation, to upregulate *IGF1*. It has been shown that the accumulation of HIF1α under hypoxia can lead to an increased production of IGF-1 [121], which can support our findings. In addition, it has been reported that IGF-1 is associated with lymphangiogenesis [122], suggesting that lymphangiogenesis is occurred in middle stage LSCC as shown in Figure 6D.

CCR7 is a chemokine receptor. The expression of CCR7 has been observed and involved in the invasion and metastasis of NSCLC [123]. It has been reported that lymph nodes can produce CCL21 and the interaction between CCL21 and CCR7 can regulate the directional migration to promote the lymph node metastasis of breast cancer [124], suggesting that CCR7 is involved in cell migration. In our result, though *CCR7* is inhibited by miR-21 in early stage LSCC and CCL21 expression is decreased in middle stage LSCC, cancer cells through the upregulation of *CCR7* in middle stage LSCC to increase the binding probability to interact with chemoattractant CCL21 can promote themselves to migrate to lymph nodes (Figure 3). Taken together, the production of IGF-1 and the activation of expression of CCR7 result in lymphangiogenesis and may promote cell migration toward lymph node in middle stage LSCC, suggesting that cancer cells may metastasize through lymph node in LSCC.

ACTA2 belongs to the actin family of proteins and is involved in the regulation of cell movement and maintenance of cell shape. The down regulation of ACTA2 could remarkably impaire *in vitro* cell invasion and migration in LADC [125]. Our results indicate that ACTA2 is activated by the CREBBP-induced acetylation, which can potentially promote the invasive ability of cancer cells by the activation of cell migration in early stage LSCC. In addition, Tensin1 (TNS1), a focal-adhesion molecule, is involved in the negative regulation of cell migration, and the reduced expression of TNS1 has been observed in human breast carcinoma, head and neck squamous cell carcinoma [126]. Our results show that *TNS1* is inhibited by a lower expression of miR-21 in early stage LSCC. However, with a lower basal level in middle stage, *TNS1* has a significant basal level difference between early and middle stage LSCC reflecting that the repressive ability on this gene might be caused by DNA methylation, resulting in the inhibition of *TNS1* in middle stage LSCC, and suggesting that the downregulated *TNS1* can reduce the inhibition of miR-21 to induce cell migration. According to comparing DNA methylation profiles of early stage and middle stage LSCC, the significant change of methylation level in *TNS1* (*p*-value < 6.8 × 10^–3^) can also support this result.

MiR-21 plays a role in tumorigenesis, tumor cell proliferation, and invasion. With a higher basal level in middle stage, *miR-21* has significant basal level difference between early and middle stage LSCC. In other words, *miR-21* may be activated by DNA methylation resulting in the higher expression in middle stage LSCC, which indicates the activation of miR-21 can promote cancer cells invasion and proliferation in middle stage LSCC. According to comparing DNA methylation profiles of early stage and middle stage LSCC, the significant change of methylation level in *miR-21* (*p*-value < 2.6 × 10^–2^) can support this result. Matrix Metallopeptidase 2 (MMP-2) plays a key role in the promotion of cancer invasion and metastasis through the degradation of ECM to allow cancer cells to move around. Our results show that *MMP2* is inhibited by miR-106b in early stage LSCC. While in middle stage LSCC, *MMP2* is upregulated by the regulation of TF JunB in the aberrance of miR-106b and is also affected by DNA methylation, suggesting that the dysfunction of miR-106b can cause the abnormal ECM degradation to potentially promote the invasion and metastasis of cancer cells.

*ITGB1*, Integrin Subunit Beta 1, encodes protein CD29 and plays an important role in cancer cell motility, survival, and attachment. It has been shown that the inhibition of *ITGB1 in vitro* and *in vivo* can decrease lung cancer invasion and metastasis [127, 128]. In our results, with a higher basal level in middle stage, *ITGB1* has a significant basal level difference between early and middle stage LSCC reflecting that the active ability on this gene might be caused by DNA methylation, indicating that the upregulation of *ITGB1* caused by DNA methylation can induce cell migration in middle stage LSCC. According to comparing DNA methylation profiles of early stage and middle stage LSCC, the significant change of methylation level in *ITGB1* (*p*-value < 5.9 × 10^–3^) can also support this result.

### Microenvironment change, dysregulation of miRNA/lncRNA regulation, DNA methylation, and epigenetic modification contribute to genetic and epigenetic progression mechanisms from middle stage to progress to advanced stage of LADC and LSCC

As shown in Figure 5E and 5F, we identified that the altered tumor microenvironment caused by hydrogen peroxide secreted by cancer cells or cancer associated fibroblasts (CAFs) can potentially cause middle stage LADC to progress to advanced stage LADC, while the altered tumor microenvironment caused by oxidative stress induced by the stimulation of nicotine derived nitrosaminoketone (NNK) can potentially cause middle stage LSCC to progress to advanced stage LSCC. In LADC, the altered tumor microenvironment caused by hydrogen peroxide can potentially cause the dysregulation of EGFR, EpoR, and ITGB1 (CD29) signaling pathways (Figure 6E). Besides, the acetylation of ZEB1, deacetylation of RHOB, dysregulation of miR-143HG, and miR-19a, and DNA methylation of *PCK1* could also result in abnormal cellular functions, such as proliferation, gluconeogenesis, cell migration, and extracellular proteolysis, causing middle stage LADC to progress to advanced stage LADC. In LSCC, the altered tumor microenvironment caused by oxidative stress induced by the stimulation of NNK can potentially cause the dysregulation of ERBB4, PDPN, and EMR2 signaling pathways (Figure 6E). Moreover, the ubiquitination and deacetylation of GATA3, deacetylation of FOXL1, and deubiquitination of CDH3, dysregulation DNA methylation in *PDPN*, and dysregulation of miR-9-2 could also result in abnormal cellular functions, such as proliferation, cell mobility, cell adhesion, and lymphangiogenesis, causing middle stage LSCC to progress to advanced stage LSCC.

Interestingly, based on our results in LADC, we found that gluconeogenesis is activated during the progression from middle stage to advanced stage LADC. It has been reported that the tumor cells have a lower concentration of glucose about 3 times compared to the corresponding normal tissues [129]. For maintaining their cellular functions such as cell proliferation and migration, cancer cells activate gluconeogenesis to gain the energy. Besides, similar to the progression from early stage to middle stage, cell migration through EMT has also been observed by the activated CDH2, a mesenchymal cadherin involved in the EMT. Through secreting PLAU by cancer cells, the surrounding matrix could be reorganized via proteolysis, hence allowing LADC cancer cells to invade and metastasize to other locations. In LSCC, we found LSCC cancer cells may undergo a collective cell migration (collective cell migration associated proteins CDH3 and PDPN in Figure 6E) to invade or metastasize to the surrounding or other tissue. Since the migrating squamous cell carcinoma can usually be observed to form large complexes of cells during the progression of lung cancer [130], it is not surprising that the collective cell migration has been implicated in LSCC during the progression from middle stage to advanced stage LSCC in our results, From our results, we showed that LADC and LSCC can undergo different ways to move in different cancer microenvironment. The LADC cancer cells can mediate EMT-associated genes to undergo EMT, indicating that the LADC cancer cells may invade or metastasize to other sites through single cell migration, while the LSCC cancer cells may invade or metastasize to other sites through clustered cells migration (collective cell migration) through the mediation of collective migration associated proteins.

### Dysfunctions of miRNA regulation, DNA methylation, epigenetic modification and microenvironment alteration contribute to the progression from early stage to middle stage LADC

#### The hydrogen peroxide secreted by cancer cells leads to the alteration of tumor microenvironment, causing dysregulation of EGFR, EPOR and ITGB1 (CD29) signaling pathways to progress from middle stage to advanced stage of LADC

As shown in Figure 6E, our results reveal that the altered tumor microenvironment caused by hydrogen peroxide secreted by cancer cells and the dysregulation of genetic and epigenetic regulation could lead to the alteration of EGFR, EPOR, and CD29 signaling pathways, resulting abnormal proliferation, gluconeogenesis, cell migration, and extracellular proteolysis to cause early stage LADC to progress to middle stage LADC.

In middle stage LADC, receptor EGFR can be activated by interacting with ligands EGF, which may be secreted by nearby macrophage stimulated by hypoxia, and BGN, which may be secreted by tumor endothelial cells. The activated receptor EGFR then triggers the TF Androgen Receptor (AR) through protein PCK1 and TF MYC through cascade proteins RHPN2, MTOR, and EIF4B in the corresponding signaling pathways, to regulate proliferation through the mediation of proliferation-related gene *EIF4B*. However, the secretion of the hydrogen peroxide by nearby cancer cells and cancer-associated fibroblasts (CAFs) could cause the DNA damage, inflammation to the altered tumor microenvironment, and cancer metabolism [131–135]. It has been reported that hydrogen peroxide is a carcinogen and may function as “fertilizer” to result in the promotion of tumor growth, progression and metastasis [136]. After being affected by hydrogen peroxide, the altered microenvironment could cause the dysregulation of EGFR, EpoR, and ITGB1 (CD29) signaling pathways.

In advanced stage LADC in Figure 6E, receptor EPOR binds to its ligand Erythropoietin (EPO) to trigger TF STAT3 through cascade proteins RHOB and XRCC4 in the corresponding signaling pathways, to regulate proliferation through the mediation of *RHOB*. Erythropoietin (EPO) is a 30.4-kD glycoprotein. It has been reported that hydrogen peroxide (H_2_O_2_) participates in the stimulation of EPO production and *EPO* expression regulation in the hepatocellular cells [137]. EpoR is a member of the cytokine receptor family. The human and experimental studies have revealed that EPO and EpoR can co-express in a diversity of human malignancies and the EPO/EpoR signaling is involved in cancer cell proliferation, inhibition of apoptosis, and invasiveness [138].

In addition, ITGB1 is activated by the interaction with ligand BGN secreted from nearby tumor endothelial to directly trigger the TF MYC and to indirectly trigger TF ZEB1 through cascade proteins, IQGAP1, CNIH4, and RHPN2 in the corresponding signaling pathways, to regulate cell migration through the mediation of *miR-19a* and *CDH2*, to regulate gluconeogenesis through the mediation of *PCK1*, to regulate extracellular proteolysis through the mediation of *PLAU*, and to regulate proliferation through the mediation of *EIF4B* and *RHOB*. Biglycan (BGN) is a small leucinerich repeat proteoglycan family of proteoglycans. In Figure 6E, BGN can be secreted from tumor endothelial cells (TECs) and is involved in cell migration, matrix assembly, and adhesion to promote lung tumors metastasis by enhancing tumor cell intravasation [139–142]. MiR-19a is involved in the regulation of cancer functions, for example, miR-19a can enhance cell invasion and migration, cell growth, and viability in NSCLC [143]. However, our results show that not only the dysregulation of EGFR, EPOR, and ITGB1 signaling pathways, which are affected by the alteration of microenvironment, but also the dysfunctions of miRNA regulation, DNA methylation, and epigenetic modification can cause middle stage LADC to progress to advanced stage LADC. The epigenetic dysfunctions of miRNA regulation, DNA methylation, and epigenetic modification of LADC are described as follows.

### Dysregulations of miR-143HG and miR-19a, epigenetic modifications of ZEB1 and RHOB, and DNA methylation of PCK1 contribute to the progression from middle stage to advanced stage LADC

The plasminogen activator (PLAU), uPA, can mediate proteolysis and lead to cancer cell invasion and metastasis. Our results show that *PLAU* is inhibited by miR-134HG in middle stage LADC. However, in advanced stage LADC, *PLAU* is upregulated in the aberrant of miR-143HG silencing, suggesting that the dysfunction of miR-143HG can lead to the activation of extracellular proteolysis through the mediation of *PLAU*. Therefore, we revealed that the matrix surrounding cancer cells can be degraded by proteolysis regulated by PLAU. This allows cancer cells to invade surroundings easily to promote cancer cells metastasis (Figure 6E). In advanced stage LADC, the ESCO1-induced acetylation of ZEB1 leads to the activation of proliferation, gluconeogenesis, cell migration, and extracellular proteolysis through the upregulation of proliferation-related gene *EIF4B*, extracellular proteolysis-related gene *PLAU*, gluconeogenesis-related gene *PCK1*, and migration-related gene *CDH2*, indicating that these activated cellular functions caused by the acetylation of ZEB1 can promote LADC cancer cells invasion and metastasis.

CDH2, a member of the cadherin superfamily, which is known as N-cadherin, is a mesenchymal cadherin involved in the EMT and metastasis of cancer cells [144]. It has been shown that the expression of CDH2 may potentially cause mesenchymal phenotype by EMT and the promotion of the survival of lung cancer cells with drug-resistant [145, 146], suggesting that CDH2 is a potential therapy target for anti-cancer cells. PCK1, an isoform of PEPCK, is involved in the control of gluconeogenesis [147]. Our results show that *PCK1*, with a higher basal level in advanced stage LADC, has a significant basal level difference between middle stage and advanced stage LADC reflecting that the active ability on this gene might be caused by DNA methylation. In other words, it suggests that the upregulated *PCK1* caused by DNA methylation can increase gluconeogenesis in middle stage LADC. Gluconeogenesis plays a key role in development of cancer cells. Due to high consumption, cancer cells face the limitation of nutrients, such as glucose. Comparing to the corresponding normal tissues, the concentration of glucose is lower about 3 times in tumor cells [129]. However, cancer cells need energy to maintain their function such as proliferation and cell migration. Hence cancer cells need gluconeogenesis to adapt and survive in the change of microenvironment.

Besides, our results also show that RHOB is repressed in middle stage by deacetylation. While in advanced stage LADC, *RHOB* is also inhibited by a higher expression of miR-19a. The small GTP binding protein RHOB belongs Rho protein family and is involved in the regulation of diverse cellular processes including cell cycle progression, cytoskeletal organization, cytokinesis, and gene transcription [148, 149]. The downregulated *RHOB* has been observed in lung cancer, leading to the loss of its expression [150–152]. In lung cancer cell lines, the ectopic expression of RHOB can suppress anchorage-independent growth and cell proliferation [150]. It has been suggested that RHOB expression is controlled by epigenetic regulation. Wang *et al*. demonstrated that the repression of RHOB expression is caused by histone deacetylase 1 (HDAC1) in lung cancer cell lines [152]. Besides, recent study showed that the significant RHOB re-expression is induced by histone deacetylase (HDAC) inhibitors in lung cancer cell lines, indicating the expression of RHOB is mainly regulated by deacetylation and can support out results. Hence we suggest that RHOB is inhibited in both middle stage and advanced stage LADC to induce abnormal proliferation by the epigenetic regulation of deacetylation and dysfunction of miR-19a, respectively.

### Dysfunctions of miRNA regulation, DNA methylation, epigenetic modification and microenvironment alteration contribute to the progression from middle stage to advanced stage LSCC

#### The oxidative stress induced by the stimulation of nicotine derived nitrosaminoketone (NNK) can potentially lead to the alteration of microenvironment, causing dysregulation of ERBB4, PDPN, and EMR2 signaling pathways from middle stage to advanced stage LSCC

As shown in Figure 6F, our results reveal that the oxidative stress induced by the stimulation of nicotine derived nitrosaminoketone (NNK) and the dysregulation of genetic and epigenetic regulation could lead to the dysregulation of ERBB4, PDPN, and EMR2 signaling pathways, resulting in abnormal proliferation, gluconeogenesis, cell migration, and extracellular proteolysis to cause middle stage LSCC to progress to advanced stage LSCC.

In middle stage LSCC in Figure 6F, EMR2 is activated by interaction with ligand DDX5 under the hypoxia. The activated EMR2 then triggers TF FOXL1 through cascade proteins CHD3 and AGR3 in the signaling pathway, to regulate proliferation and cell mobility through the mediation of *NF1*. The DEAD-box-protein DDX5, an ATP-dependent RNA helicase, has been involved in the promotion of proliferation and tumorigenesis in NSCLC [153]. CHD3, a catalytic component of histone deacetylase complex, is involved in tumorigenesis and metastasis [154]. Besides, under the microenvironment stresses and DNA damage, anterior gradient protein 3 (AGR3) can promote the survival of tumor cells and may participate in the coordination of cell proliferation, survival, and metastasis [155, 156]. After binding to TGFB1, receptor ERBB4 is activated to trigger TFs YBX1 and GATA3 through cascade proteins TGFB1, CDC42, LIN7C, FN1, and NF1 in the corresponding signaling pathways. These TFs would regulate cell mobility through the mediation of *PDPN*, cell adhesion through mediation of *CDH3*, and proliferation through the mediation of *AGR3*. However, tumor microenvironment is altered due to oxidative stress induced by stimulation of NNK, a lung carcinogen. The altered microenvironment, causing the alteration of ERBB4, PDPN, and EMR2 signaling pathway (Figure 6F).

In advanced stage LSCC, the stress-responsive transcription factor FOXO3 (also known as FOXO3A) is activated. It has been reported that the Forkhead box type-O (FOXO) family of transcription factors can respond to these stresses, including DNA damage and oxidative stress [157–160]. Evidence suggests that FOXO3A is involved in the regulation of cellular response to these stresses, such as DNA repair, apoptosis, and growth arrest [161–163]. It has been demonstrated that FOXO3A can be activated in response to nicotinederived nitrosaminoketone (NNK), a carcinogen, and plays a role in anti-carcinogenic response by the suppression of NNK-induced DNA damage in LADC [161], suggesting that FOXO3A is activated in response to NNK in advanced stage LSCC. ERBB4 is activated by interacting with COL18A1, which also interacts with FN1, to trigger TF GATA3 by the modulation of protein TGFB1. Besides, the activated receptor ERBB4 can also affect glycoprotein PDPN to trigger TFs, GATA3 and FOXO3 through cascade proteins CHIC2, Moesin (MSN), TUBB3, RHOXF2, SMG1 in the signaling pathways, to regulate proliferation through the mediation of *FOXO3*, *SMG1*, and *NF1*, to regulate cell mobility through the mediation of *NF1*, to regulate cell adhesion through the mediation of *CDH3*, and to regulate lymphangiogenesis through the mediation of *VEGFC* (Figure 6F). COL18A1 encodes Type XVIII collagen, also known as Endostatin, a potent antiangiogenic protein that is able to inhibit angiogenesis and is associated with malignant pleural effusions in patients of lung cancer [164, 165]. Besides, COL18A1 is a regulator of the oxidative stress response and has been suggested that the potential links between ECM and oxidative stress response in cancer. However, our results show that not only the dysregulation of ERBB4, EMR2, and PDPN signaling pathways which are affected by the alteration of microenvironment but also the dysfunctions of miRNA regulation, DNA methylation, and epigenetic modification may be involved in the progression from middle stage to advanced stage LSCC. The epigenetic dysfunctions of miRNA regulation, DNA methylation, and epigenetic modification of LSCC are described as follows.

#### Dysregulation of miR-9-2, epigenetic modifications of GATA3, FOXL1, and CDH3, and DNA methylation of PDPN contribute to the progression from middle stage to advanced stage LSCC

In middle stage LSCC in Figure 6F, TF GATA3 is modified by the SIAH1-induced ubiquitination and HDAC5-induced deacetylation, leading to the degraded GATA3 to inhibit cell adhesion and proliferation through the mediation of *CDH3* and *AGR3*. However, in advanced stage LSCC, GATA3 is not affected by ubiquitination and deacetylation, hence causing the activation of GATA3 in advanced stage LSCC to activate lymphangiogenesis and proliferation through the mediation of *VEGFC*, *SMG1*, and *FOXO3*, indicating that these activated functions may increase the risk of lymphatic metastasis. Serine/threonine protein kinase 1 (SMG 1) belongs to the phosphatidylinositol 3 kinase related kinase family and the knockdown expression of SMG1 can result in the inhibition of tumor cell proliferation in cancer [166].

FOXL1 belongs to the forkhead/winged helix-box (FOX) family and plays an important role in the regulation of cell proliferation. Our results show that the HDAC9-induced deacetylation of FOXL1, which is also indirectly modified by the CHD3-induced deacetylation via AGR3, results in the inhibition of FOXL1. Hence, cell mobility and proliferation are activated by the downregulation of *NF1* via the inhibited FOXL1 in middle stage LSCC. It is noted that this promote middle stage cancer cells to advanced stage LSCC cancer cells potentially. Neurofibromatosis type 1 (NF1) plays negative regulatory role in the downstream of EGFR signaling and Ras cellular proliferation pathways [167–170]. Besides regulating cell proliferation, NF1 is also involved in the regulation of actin cytoskeletal reorganization to affect cell adhesion and motility. Moreover, NF1 siRNA can cause morphological change, which is the excessive formation of actin stress fiber [171].

Moreover, our results also show that the podoplanin (PDPN) is inhibited in middle stage caused by the inhibition of activated miR-199b and HERC4-induced ubiquitination. While in advanced stage LSCC, PDPN is activated by the modification of SIRT1-induced deacetylation and DNA methylation. Our results reveal that PDPN, with a higher basal level in advanced stage LSCC, has a significant basal level difference between middle stage and advanced stage LSCC reflecting that the activity ability of this gene might be caused by DNA methylation. *PDPN* encodes a type-I integral membrane glycoprotein, and its expression is upregulated in squamous cell carcinoma of lung [172]. Previous study indicates that PDPN participates in the regulation of cell motility and cytoskeletal organization by mediating ERM proteins [173]. Our results in Figure 6F indicated that membrane glycoprotein PDPN can trigger the ERM protein MSN through CHIC2 to facilitate cell motility and cytoskeletal organization, which can be supported by previous study. In addition, the expression of PDPN by tumor cells can induce tumor lymphangiogenesis and increase lymph nodes metastasis [174]. Moreover, it has been reported that PDPN can mediate collective cell migration (CCM) to promote tumor invasion without EMT [173].

In addition, CDH3 (P-cadherin) is inhibited by miR-330 silencing and the regulation of GATA3 in middle stage LSCC. While in advanced stage LSCC, CDH3 is activated by the modification of OTUD1-induced deubiquitination, though it is also affected by DNA methylation. CDH3, which belongs to the cadherin superfamily, is a classical cell-to-cell adhesion molecule. It has been observed that the overexpression of CDH3 (P-cadherin) is associated with the aggressiveness, invasion, and metastasis of cancer cells [175–179]. Besides, recent studies indicate that P-cadherin can promote collective cell migration (CCM) and the depletion of P-cadherin in epithelial cells could significantly induce the impair of CCM in 2D and 3D culture systems, in *in vitro* [180–182]. Our results demonstrate that both CDH3 and PDPN are activated in advanced stage LSCC, suggesting that LSCC cancer cells may induce collective migration (CCM) to invasion and metastasis.

Moreover, FOXO3 can respond to nicotine-derived nitrosaminoketone (NNK) and is activated to inhibit proliferation through the mediation of *NF1*. However, *FOXO3* affected by DNA methylation is inhibited by the activated miR-9-2 in advanced stage LSCC. The inhibited FOXO3 then leads to the downregulation of *NF1*, causing the activation of proliferation and cell mobility. Hence, it promotes growth, invasion and metastasis in LSCC cancer cells. EMR2, a member of class B seven-span transmembrane (TM7) subfamily, is modified by USP20-induced deubiquitination. It has been reported that the expression of EMR2 is associated with invasive carcinomas and may play a key role in the contribution of invasive phenotype [183, 184]. Hence the modification of deubiquitination may lead to the dysregulation of EMR2 signaling, causing the abnormal proliferation and cell mobility to induce the progression from middle stage to advanced stage LSCC.

As discussed in the above, we summarized the differential progression genetic and epigenetic mechanisms in LADC and SCC in Figure 5, indicating that the progression from normal stage to early stage LADC can be potentially caused by inflammatory microenvironment induced by bacteria infection (LPS), the dysfunctions of EGFR and TLR4 signaling, the regulation of miR-130b, miR-100HG, and miR-1292, the epigenetic modifications of E2F1 and SFTPA2, the DNA methylation of *MYC* and *RB1*, and the aberrant cellular functions, such as cell cycle. While the progression from normal stage to early stage of LSCC can be potentially caused by the inflammatory microenvironment induced by the exposure to xenobiotic toxicity nicotine, the dysfunctions of FGFR1, DDR1, and CHRNA5 signaling, the regulation of miR-24-2 miR-1247, and lncRNA TUG1, the epigenetic modifications of KLF12 and ERCC1, the DNA methylation of *SHOX2*, and the aberrant cellular functions, such as DNA repair. The progression from early stage to middle stage LADC can be potentially induced by the hypoxic tumor microenvironment, the dysfunctions of NOTCH1 and ITGA4 (CD49d) signaling, the regulation of miR-30c-2 and miR-27b, the epigenetic modifications of VIM, MYH9, MDM4, and ETS1, and the dysregulation of cellular functions, such as angiogenesis, ECM remodeling, and EMT. While the progression from early stage to middle stage LADC can be potentially induced by the hypoxic tumor microenvironment exposed to nicotine, the dysfunctions of IGF-1R, ITGB1 (CD29), and TNS1 signaling, the regulation of miR-106b, the epigenetic modifications of HIF1α, IGF-1, and ETS1, the DNA methylations of *ITGB1*, *TNS1*, and *miR-21*, and the dysregulation of cellular functions, such as lymphangiogenesis, cell migration, and ECM degradation. Besides, the progression from early stage to middle stage LADC can be potentially induced by the alteration of tumor microenvironment caused by hydrogen peroxide secreted by cancer cells, the dysfunctions of EGFR, EPOR, and ITGB1 (CD29) signaling, the regulation of miR-143HG and miR-19a, the epigenetic modifications of ZEB1 and RHOB, the DNA methylation of *PCK1*, and the dysregulation of cellular functions, such as gluconeogenesis and extracellular proteolysis. While the progression from early stage to middle stage LSCC can be potentially induced by the alteration of tumor microenvironment caused by oxidative stress induced by the stimulation of nicotine derived nitrosaminoketone (NNK), the dysfunctions of ERBB4, PDPN, and EMR2 signaling, the regulation of miR-9-2, the epigenetic modifications of GATA3, FOXL1, and CDH3, the DNA methylation of *PDPN,* and the dysregulation of cellular functions, such as proliferation, cell mobility, and cell adhesion.

### Identification of network biomarkers for the discovery of genetic and epigenetic multiple drugs for therapeutic treatment of early stage, middle stage, and advanced stage LADC and LSCC

According to the genetic and epigenetic progression mechanisms based on LADC and LSCC core signaling pathways between connective stages (normal stage, early stage and middle stage), we could identify network biomarkers based on the proteins with significant differential expression change between later stage and normal stage. We then found some significant network biomarkers as follows: the proteins, SLC22A18, TERT, SEPT3, RET, POLR2D, E2F1, which have significant differential expression change with higher expression in early stage compared to normal stage, and proteins, PDGFB, EGFL7, S100A10, PDGFRA, TLR4, TLR4, CCL2, PER1, RB1, TGFB1, PIK3R3, SFTPA2, MYC, which have significant expression change with lower expression in early stage compared to normal stage (Figure 2), are selected as biomarkers in core signaling pathways between normal stage and early stage LADC; the proteins, HP, LTA, APH1A, COL22A1, MMP8, IGF2BP1, HIF1A, PRDM14, which have significant expression change with higher expression in middle stage compared to normal stage, and proteins, NOTCH1, HGF, ITGA4, VIM, SLC6A20, MYH9, ETS1, ZEB1, which have significant expression change with lower expression in middle stage compared to normal stage (Figure 3), are selected as biomarkers in core signaling pathways between early stage and middle stage LADC; the proteins, EGF, CDH2, LOXL2, RHPN2, XRCC4, CNIH4, MTOR, which have significant expression change with higher expression in advanced stage compared to normal stage, and proteins, BGN, ITGB2, EPOR, IQGAP1, FN1, RHOB, TSGA10, AR, ZEB1, STAT3, which have significant expression change with lower expression in advanced stage compared to normal stage (Figure 4), are selected as biomarkers in core signaling pathways between middle stage and advanced stage LADC.

The proteins, FGF19, COL3A1, DDR1, CHRNA5, RPL30, CIAPIN1, GSK3B, IGF2BP3, SLC6A11, ERCC1, SUSD4, COL3A1, TERT, SHOX2, YBX1, SP1, ANLN, TP63, which have significant expression change with higher expression in early stage compared to normal stage, and proteins, FGF2, FGFR1, PDGFB, PDGFRB, FKBP5, DAPK3, PSMBB9, ETS1, which have significant expression change with lower expression in early stage compared to normal stage, are selected as biomarkers in core signaling pathways between normal stage and early stage LSCC (Figure 2); the proteins, COL1A1, JAG1, IGF1, IGF1R, COL1A1, TRAF4, SUSD4, PARG, HIF1A, which have significant expression change with higher expression in middle stage compared to normal stage, and proteins, TNS1, ACTA2, CCL21, CCR7, MYLK, PCDP1, ITGB1, TSC2, GATA2, JUN, ETS1, JUNB, which have significant differential expression change with lower expression in middle stage compared to normal stage, are selected as biomarkers in core signaling pathways between early stage and middle stage LSCC (Figure 3); the proteins, CDH3, CHIC2, TUBB3, LIN7C, YBX1, which have significant differential expression change with higher expression in advanced stage compared to normal stage, and proteins, FN1, ITGA8, TGFB1, ERBB4, DDX5, EMR2, MSN, CHD3, AGR3, FOXO3, GATA3, which have significant differential expression change with lower expression in advanced stage compared to normal stage, are selected as biomarkers in core signaling pathways between middle stage and advanced stage LSCC (Figure 4).

To treat early stage, middle stage, and advanced stage of LADC and LSCC from cancer cells back to normal cells, we aim to repress the genes, which have significant differential expression change between normal stage and later stage with higher expression in later stage compared to normal stage, and activate the genes, which have significant differential expression change between normal stage and later stage with lower expression in later stage compared to normal stage without affecting the expression of housekeeping genes for minimizing the side effect. Connectivity Map (CMap) database could provide the level of 14,825 genes under 6,100 different conditions containing 1,327 different compounds (i.e. drugs) and different concentrations of these compounds [185]. The correlation coefficients between the gene expression levels and the concentrations of compounds denote the relationship between compounds and genes. If the correlation coefficient is greater than zero, the gene is said to be up-regulated by applying the compound. If the correlation coefficient is less than zero, the gene is said to be down-regulated by applying the compound. It is noted that we divide the selected biomarkers into two groups. The first group is the pool which we want to upregulate (activate). The second group is the pool which we want to downregulate (repress). After applying correlation coefficient between drug concentration and mRNA activity in microarray data of CMap, we then rank 1,327 different compounds (i.e. drugs) based on the satisfaction of our restoration conditions for two groups without affecting gene expression of housekeeping genes by the computational method. Consequently, combined with literature survey to find drugs which have been approved by the Food and Drug Administration (FDA), used in cancer-related therapeutic treatment and reported of having potential anti-cancer properties from the top 20 ranked drugs, we design potential genetic and epigenetic multiple-molecule drug for the therapeutic treatment in early, middle, and advanced stage of LADC and LSCC, respectively.

From core signaling pathways of early stage LADC in Figure 2, we identified a genetic and epigenetic multiple-molecule drug including hydralazine, ketoconazole, and promethazine for potential genetic and epigenetic multiple-molecule drug targets, SLC22A18, TERT, RET, POLR2D, E2F1, PDGFB, EGFL7, S100A10, PDGFRA, TLR4, CCL2, PER1, RB1, TGFB1, SFTPA2, and MYC in core signaling pathways between normal stage and early stage LADC (Table 1). It has been reported that hydralazine, a DNA demethylating agent, was approved by FDA and used in the therapeutic treatment of cancer patient by the combination with a therapeutic dose of valproic acid to mediate the epigenetic modification in tumor cells [186]. Ketoconazole can block the dysregulated cellular metabolism to inhibit the progression of prostate cancer and may be a useful adjunct in treating lung cancer and breast cancer [187, 188]. Besides, promethazine may also be used in treating cancer by modulating energy metabolism in malignant cells [189]. Through the therapeutic treatment using the proposed genetic and epigenetic multiple-molecule drug, the proteins with significant higher expression in the early stage (SLC22A18, TERT, RET, POLR2D, and E2F1) can be repressed, and proteins with significant lower expression in the early stage (PDGFB, EGFL7, S100A10, PDGFRA, TLR4, CCL2, PER1, RB1, TGFB1, SFTPA2, and MYC) can be activated to facilitate the restoration of early stage LADC to normal lung cells.

**Table 1:** Design of genetic and epigenetic multiple drug for the therapeutic treatment of early stage LADC

Early stage LADC		
Drug target		
SLC22A18, TERT, RET, POLR2D, E2F1, PDGFB, EGFL7, S100A10, PDGFRA, TLR4, CCL2, PER1, RB1, TGFB1, SFTPA2, MYC		

Drug molecule		
Drug Name	Repressed drug target	Activated drug target
Hydralazine	E2F1, SLC22A18, TERT	CCL2, EGFL7, MYC, PDGFB, PER1, RB1, S100A10, SFTPA2, TGFB1
Ketoconazole	E2F1, POLR2D, RET, SLC22A18, TERT	CCL2, PDGFB, PDGFRA, S100A10, SFTPA2, TGFB1, TLR4
Promethazine	E2F1, POLR2D, RET, SLC22A18, TERT	CCL2, PDGFB, PDGFRA, RB1, S100A10, TGFB1, TLR4

**Chemical structures of multiple-molecule drug**

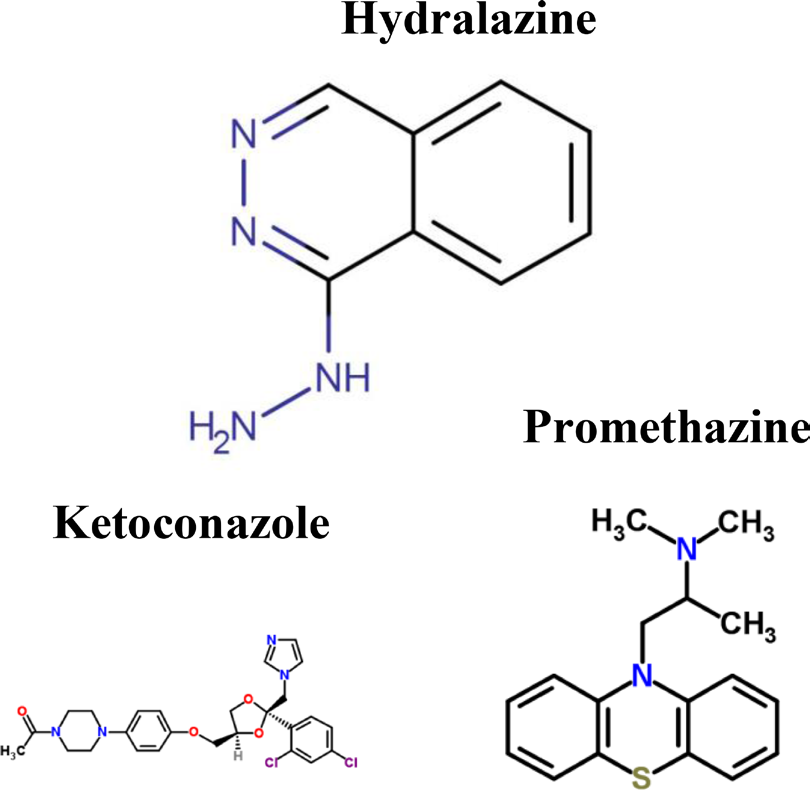

Through therapeutic treatment using the proposed genetic and epigenetic multiple-molecule drug, the proteins with significantly higher expression in the early stage (SLC22A18, TERT, RET, POLR2D, and E2F1) can be repressed, and proteins with significant lower expression in the early stage (PDGFB, EGFL7, S100A10, PDGFRA, TLR4, CCL2, PER1, RB1, TGFB1, SFTPA2, and MYC) can be activated to facilitate the restoration of early stage LADC to normal lung cells.

From core signaling pathways of middle stage LADC in Figure 3, we identified a genetic and epigenetic multiple-molecule drug including betulin, nordihydroguaiaretic acid, and proadifen for potential genetic and epigenetic multiple-molecule drug targets, HP, LTA, APH1A, MMP8, HIF1A, PRDM14, NOTCH1, HGF, ITGA4, VIM, MYH9, ETS1, and ZEB1 in core signaling pathways between early stage and middle stage LADC (Table 2). It has been demonstrated that betulin was effective against tumors, including malignant melanoma, and can cause apoptosis to inhibit the growth of tumor cells [190]. Nordihydroguaiaretic acid can induce apoptosis and inhibit the growth of human lung cancer cells [191]. In addition, nordihydroguaiaretic acid can also inhibit cell migration and tumor metastasis in prostate cancer [192]. Proadifen, an inhibitor of cytochrome P450 monooxygenases, has anti-proliferative properties through influencing metabolic activity, cell number, and cell cycle progression in colon cancer [193]. Through the therapeutic treatment using the proposed genetic and epigenetic multiple-molecule drug, the proteins with significant higher expression in the middle stage (HP, LTA, APH1A, MMP8, HIF1A, and PRDM14) can be repressed, and proteins with significant lower expression in the middle stage (NOTCH1, HGF, ITGA4, VIM, MYH9, ETS1, and ZEB1) can be activated to facilitate the restoration of middle stage LADC to normal lung cells.

**Table 2:** Design of genetic and epigenetic multiple drug for the therapeutic treatment of middle stage LADC

Middle stage LADC		
Drug target		
HP, LTA, APH1A, MMP8, HIF1A, PRDM14, NOTCH1, HGF, ITGA4, VIM, MYH9, ETS1, ZEB1		

Drug molecule		
Drug Name	Repressed drug target	Activated drug target
Betulin	APH1A, HIF1A, HP, LTA, MMP8, PRDM14	HGF, ITGA4, MYH9, NOTCH1, ZEB1
Nordihydroguaiaretic acid	APH1A, HP, MMP8, PRDM14	ETS1, HGF, ITGA4, MYH9, VIM, ZEB1
Proadifen	APH1A, HIF1A, HP, LTA, MMP8, PRDM14	HGF, ITGA4, MYH9, ZEB1

**Chemical structures of multiple-molecule drug**

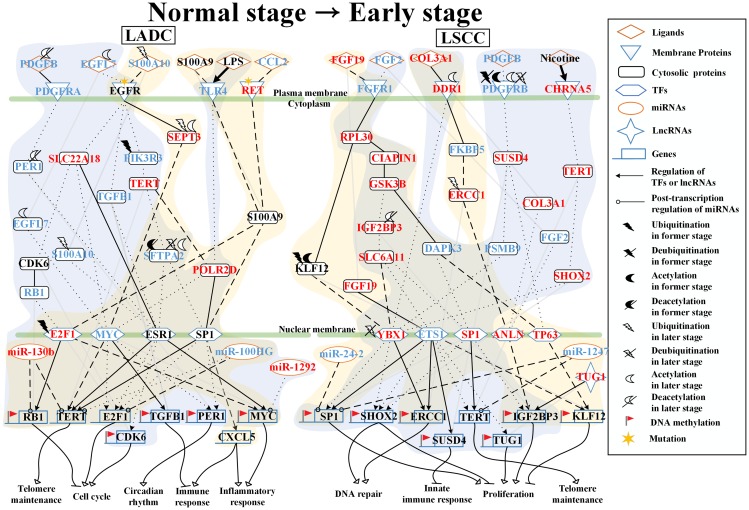

Through therapeutic treatment using the proposed genetic and epigenetic multiple-molecule drug, the proteins with significantly higher expression in the middle stage (HP, LTA, APH1A, MMP8, HIF1A, and PRDM14) can be repressed, and proteins with significant lower expression in the middle stage (NOTCH1, HGF, ITGA4, VIM, MYH9, ETS1 and ZEB1) can be activated to facilitate the restoration of middle stage LADC to normal lung cells.

From core signaling pathways of advanced stage LADC in Figure 4, we identified a genetic and epigenetic multiple-molecule drug including iloprost, methotrexate, and MK-886 for potential genetic and epigenetic multiple-molecule drug targets, EGF, CDH2, LOXL2, XRCC4, CNIH4, MTOR, BGN, ITGB2, EPOR, IQGAP1, FN1, RHOB, TSGA10, AR, and STAT3 in core signaling pathways between middle stage and advanced stage LADC (Table 3). It has been reported that the iloprost was studied in randomized phase I trial and can inhibit the transformed growth of NSCLC [194]. Methotrexate is a chemotherapy drugs called anti metabolites and can inhibit the growth of tumor cells, such as lung cancer [195, 196]. Besides, MK-886 can induce the apoptosis in gastric cancer cells and has the inhibitory ability on cell growth in a dose- and time-dependent manner [197]. Through the therapeutic treatment using the proposed genetic and epigenetic multiple-molecule drug, the proteins with significant higher expression in the advanced stage (EGF, CDH2, LOXL2, XRCC4, CNIH4, and MTOR) can be repressed, and proteins with significant lower expression in the advanced stage (BGN, ITGB2, EPOR, IQGAP1, FN1, RHOB, TSGA10, AR, and STAT3) can be activated to facilitate the restoration of advanced stage LADC to normal lung cells.

**Table 3:** Design of genetic and epigenetic multiple drug for the therapeutic treatment of advanced stage LADC

Advanced stage LADC		
Drug target		
EGF, CDH2, LOXL2, XRCC4, CNIH4, MTOR, BGN, ITGB2, EPOR, IQGAP1, FN1, RHOB, TSGA10, AR, STAT3		

Drug molecule		
Drug Name	Repressed drug target	Activated drug target
Iloprost	CDH2, CNIH4, EGF, LOXL2, MTOR, XRCC4	AR, BGN, FN1, RHOB, STAT3, TSGA10
Methotrexate	CDH2, CNIH4, LOXL2, MTOR, XRCC4	EPOR, IQGAP1, ITGB2, RHOB, STAT3, TSGA10
MK-886	CDH2, CNIH4, EGF, LOXL2, MTOR, XRCC4	AR, BGN, RHOB, STAT3, TSGA10

**Chemical structures of multiple-molecule drug**

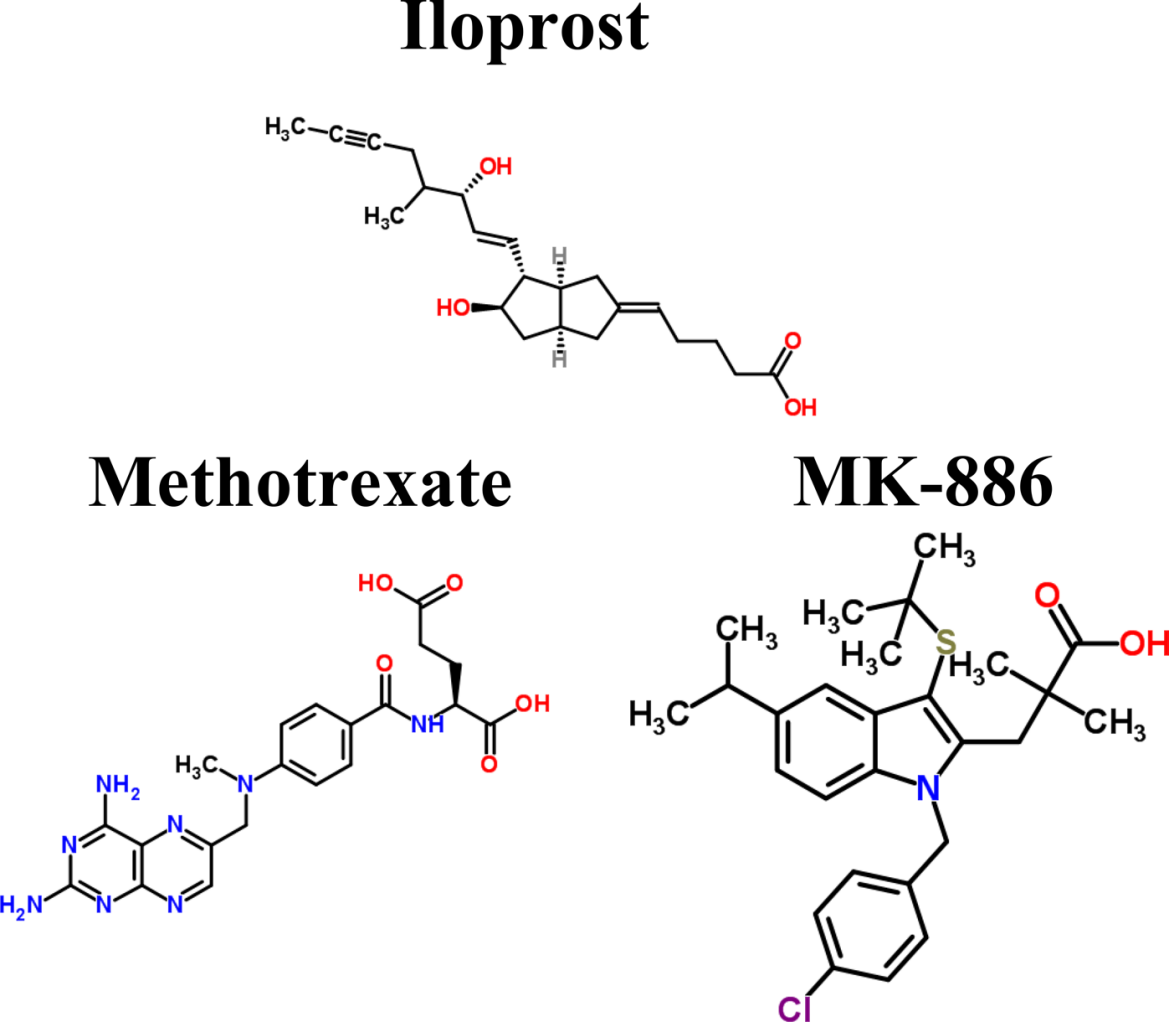

Through therapeutic treatment using the proposed genetic and epigenetic multiple-molecule drug, the proteins with significantly higher expression in the advanced stage (EGF, CDH2, LOXL2, XRCC4, CNIH4, and MTOR) can be repressed, and proteins with significant lower expression in the advanced stage (BGN, ITGB2, EPOR, IQGAP1, FN1, RHOB, TSGA10, AR, and STAT3) can be activated to facilitate the restoration of advanced stage LADC to normal lung cells.

From core signaling pathways of early stage LSCC in Figure 2, we identified a genetic and epigenetic multiple-molecule drug including pimozide, mepacrine, and repaglinide for potential genetic and epigenetic multiple-molecule drug targets, COL3A1, DDR1, CHRNA5, RPL30, CIAPIN1, GSK3B, IGF2BP3, SLC6A11, ERCC1, SUSD4, TERT, SHOX2, YBX1, SP1, TP63, FGF2, FGFR1, PDGFB, PDGFRB, FKBP5, DAPK3, and ETS1 in core signaling pathways between normal stage and early stage LSCC (Table 4). It has been shown that pimozide can inhibit prostate cancer cell proliferation through inducing G1 phase cell cycle arrest to decrease the ability of colony formation [198]. Mepacrine, an anti-proliferative agent, can lead to tumor growth inhibition in breast cancer [199]. Besides, repaglinide can inhibit the growth of tumor cells, shch as liver hepatocellular carcinoma and cervical cancer [200]. Through the therapeutic treatment using the proposed genetic and epigenetic multiple-molecule drug, the proteins with significant higher expression in the early stage (COL3A1, DDR1, CHRNA5, RPL30, CIAPIN1, GSK3B, IGF2BP3, SLC6A11, ERCC1, SUSD4, TERT, SHOX2, YBX1, SP1, and TP63) can be repressed, and proteins with significant lower expression in the early stage (FGF2, FGFR1, PDGFB, PDGFRB, FKBP5, DAPK3, and ETS1) can be activated to facilitate the restoration of early stage LSCC to normal lung cells.

**Table 4:** Design of genetic and epigenetic multiple drug for the therapeutic treatment of early stage LSCC

Early stage LSCC		
Drug target		
COL3A1, DDR1, CHRNA5, RPL30, CIAPIN1, GSK3B, IGF2BP3, SLC6A11, ERCC1, SUSD4, TERT, SHOX2, YBX1, SP1, TP63, FGF2, FGFR1, PDGFB, PDGFRB, FKBP5, DAPK3, ETS1		

Drug molecule		
Drug Name	Repressed drug target	Activated drug target
Pimozide	CHRNA5, CIAPIN1, COL3A1, DDR1, ERCC1, GSK3B, IGF2BP3, RPL30, SHOX2, SLC6A11, SP1, SUSD4, TERT, TP63, YBX1	PDGFB
Mepacrine	CHRNA5, CIAPIN1, DDR1, ERCC1, GSK3B, IGF2BP3, RPL30, SUSD4, TERT, YBX1	DAPK3, ETS1, FGF2, FGFR1, PDGFB, PDGFRB
Repaglinide	CHRNA5, CIAPIN1, COL3A1, DDR1, ERCC1, GSK3B, IGF2BP3, RPL30, SHOX2, SLC6A11, SP1, SUSD4, TERT, TP63, YBX1	FKBP5

**Chemical structures of multiple-molecule drug**

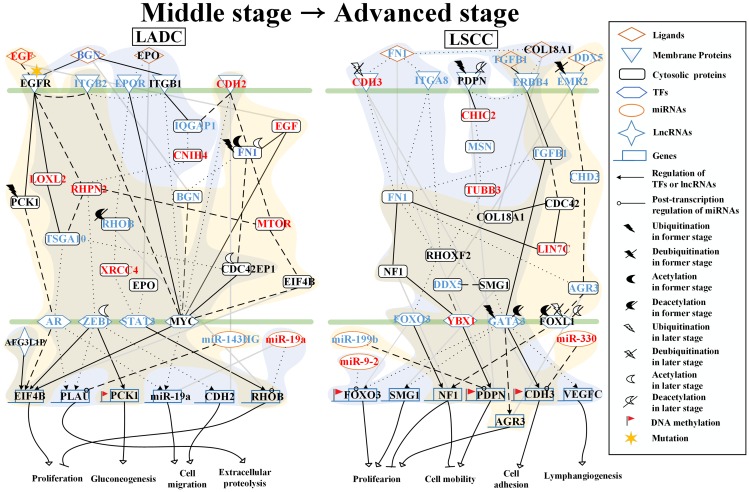

Through therapeutic treatment using the proposed genetic and epigenetic multiple-molecule drug, the proteins with significantly higher expression in the early stage (COL3A1, DDR1, CHRNA5, RPL30, CIAPIN1, GSK3B, IGF2BP3, SLC6A11, ERCC1, SUSD4, TERT, SHOX2, YBX1, SP1, and TP63) can be repressed, and proteins with significant lower expression in the early stage (FGF2, FGFR1, PDGFB, PDGFRB, FKBP5, DAPK3, and ETS1) can be activated to facilitate the restoration of early stage LSCC to normal lung cells.

From core signaling pathways of middle stage LSCC in Figure 3, we identified a genetic and epigenetic multiple-molecule drug including ribavirin, procainamide, and ketoconazole for potential genetic and epigenetic multiple-molecule drug targets, COL1A1, JAG1, IGF1, IGF1R, TRAF4, SUSD4, PARG, TNS1, ACTA2, CCL21, CCR7, MYLK, ITGB1, TSC2, GATA2, JUN, ETS1, and JUNB in core signaling pathways between early stage and middle stage LSCC (Table 5). Ribavirin can impede the oncogenic transformation of cells. It has been reported that ribavirin can cause clinical benefit in poor prognosis acute myeloid leukemia patients in a phase II clinical trial [201]. Procainamide, a drug approved by the FDA, is an inhibitor of DNA methylation and can lead to the reactivation of methylation-silenced genes and demethylation. Previous studies have been reported that procainamide may be potentially used in preventing the development of lung cancer by changing the epigenetic modification [202]. Moreover, ketoconazole can inhibit the progression of prostate cancer and may have potential use in treatment of lung cancer and breast cancer [187, 188]. Through the therapeutic treatment using the proposed genetic and epigenetic multiple-molecule drug, the proteins with significant higher expression in the middle stage (COL1A1, JAG1, IGF1, IGF1R, TRAF4, SUSD4, and PARG) can be repressed, and proteins with significant lower expression in the middle stage (TNS1, ACTA2, CCL21, CCR7, MYLK, ITGB1, TSC2, GATA2, JUN, ETS1, and JUNB) can be activated to facilitate the restoration of middle stage LSCC to normal lung cells.

**Table 5:** Design of genetic and epigenetic multiple drug for the therapeutic treatment of middle stage LSCC

Middle stage LSCC		
Drug target		
COL1A1, JAG1, IGF1, IGF1R, TRAF4, SUSD4, PARG, TNS1, ACTA2, CCL21, CCR7, MYLK, ITGB1, TSC2, GATA2, JUN, ETS1, JUNB

Drug molecule		
Drug Name	Repressed drug target	Activated drug target
Ribavirin	COL1A1, IGF1, JAG1, PARG, SUSD4	ACTA2, CCL21, CCR7, ETS1, GATA2, ITGB1, JUN, JUNB, MYLK, TNS1, TSC2
Procainamide	IGF1, IGF1R, SUSD4, TRAF4	ACTA2, CCL21, CCR7, ETS1, GATA2, ITGB1, JUN, JUNB, MYLK, TNS1, TSC2
Ketoconazole	IGF1, IGF1R, SUSD4, TRAF4	ACTA2, CCL21, CCR7, ETS1, GATA2, ITGB1, JUN, JUNB, MYLK, TNS1, TSC2

**Chemical structures of multiple-molecule drug**

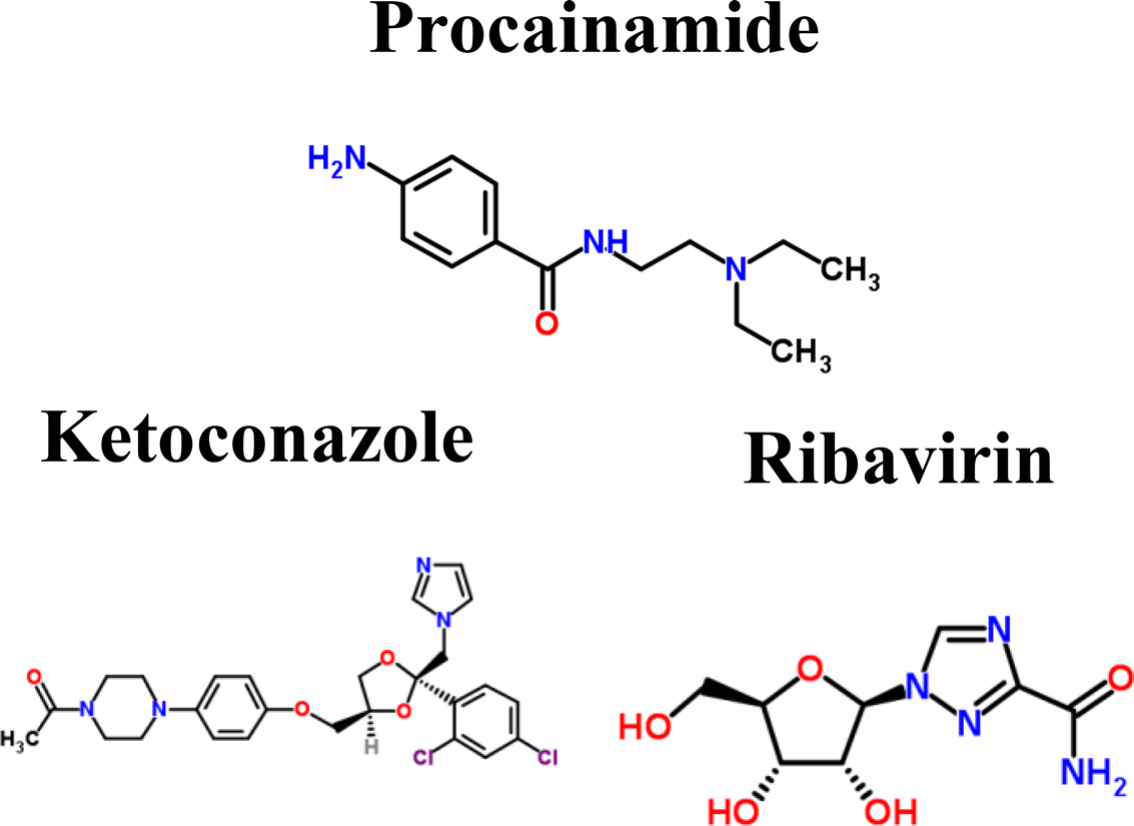

Through therapeutic treatment using the proposed genetic and epigenetic multiple-molecule drug, the proteins with significantly higher expression in the middle stage (COL1A1, JAG1, IGF1, IGF1R, TRAF4, SUSD4, and PARG) can be repressed, and proteins with significant lower expression in the middle stage (TNS1, ACTA2, CCL21, CCR7, MYLK, ITGB1, TSC2, GATA2, JUN, ETS1, and JUNB) can be activated to facilitate the restoration of middle stage LSCC to normal lung cells.

From core signaling pathways of advanced stage LSCC in Figure 4, we identified a genetic and epigenetic multiple-molecule drug including clomipramine, rolipram, and procainamide for potential genetic and epigenetic multiple-molecule drug targets, CDH3, CHIC2, TUBB3, LIN7C, YBX1, FN1, ITGA8, TGFB1, ERBB4, DDX5, EMR2, MSN, CHD3, FOXO3, and GATA3 in core signaling pathways between middle stage and advanced stage LSCC (Table 6). Clomipramine, an antineoplastic agent, can lead to cancer cell apoptosis. It has been shown that chlorimipramine has positive effects against human leukaemia cells, and human renal cancer cells [203, 204]. Besides, it has been also reported that rolipram can result in a decreased proliferation and an increased apoptosis in malignant glioma cells [205]. Procainamide, an inhibitor of DNA methylation, was approved by the FDA and was potentially used in preventing the development of lung cancer [202]. Through the therapeutic treatment using the proposed genetic and epigenetic multiple-molecule drug, the proteins with significant higher expression in the advanced stage (CDH3, CHIC2, TUBB3, LIN7C, and YBX1) can be repressed, and proteins with significant lower expression in the advanced stage (FN1, ITGA8, TGFB1, ERBB4, DDX5, EMR2, MSN, CHD3, FOXO3, and GATA3) can be activated to facilitate the restoration of advanced stage LSCC to normal lung cells.

**Table 6:** Design of genetic and epigenetic multiple drugs for the therapeutic treatment of advanced stage LSCC

Advanced stage LSCC		
Drug target		
CDH3, CHIC2, TUBB3, LIN7C, YBX1, FN1, ITGA8, TGFB1, ERBB4, DDX5, EMR2, MSN, CHD3, FOXO3, GATA3		

Drug molecule		
Drug Name	Repressed drug target	Activated drug target
Clomipramine	CDH3, CHIC2, LIN7C, YBX1	DDX5, EMR2, ERBB4, FN1, FOXO3, GATA3, ITGA8, TGFB1
Rolipram	CDH3, TUBB3	DDX5, EMR2, ERBB4, FN1, GATA3, ITGA8, MSN, TGFB1, CHD3
Procainamide	TUBB3, YBX1	DDX5, EMR2, ERBB4, FN1, GATA3, ITGA8, MSN, TGFB1, CHD3

**Chemical structures of multiple-molecule drug**

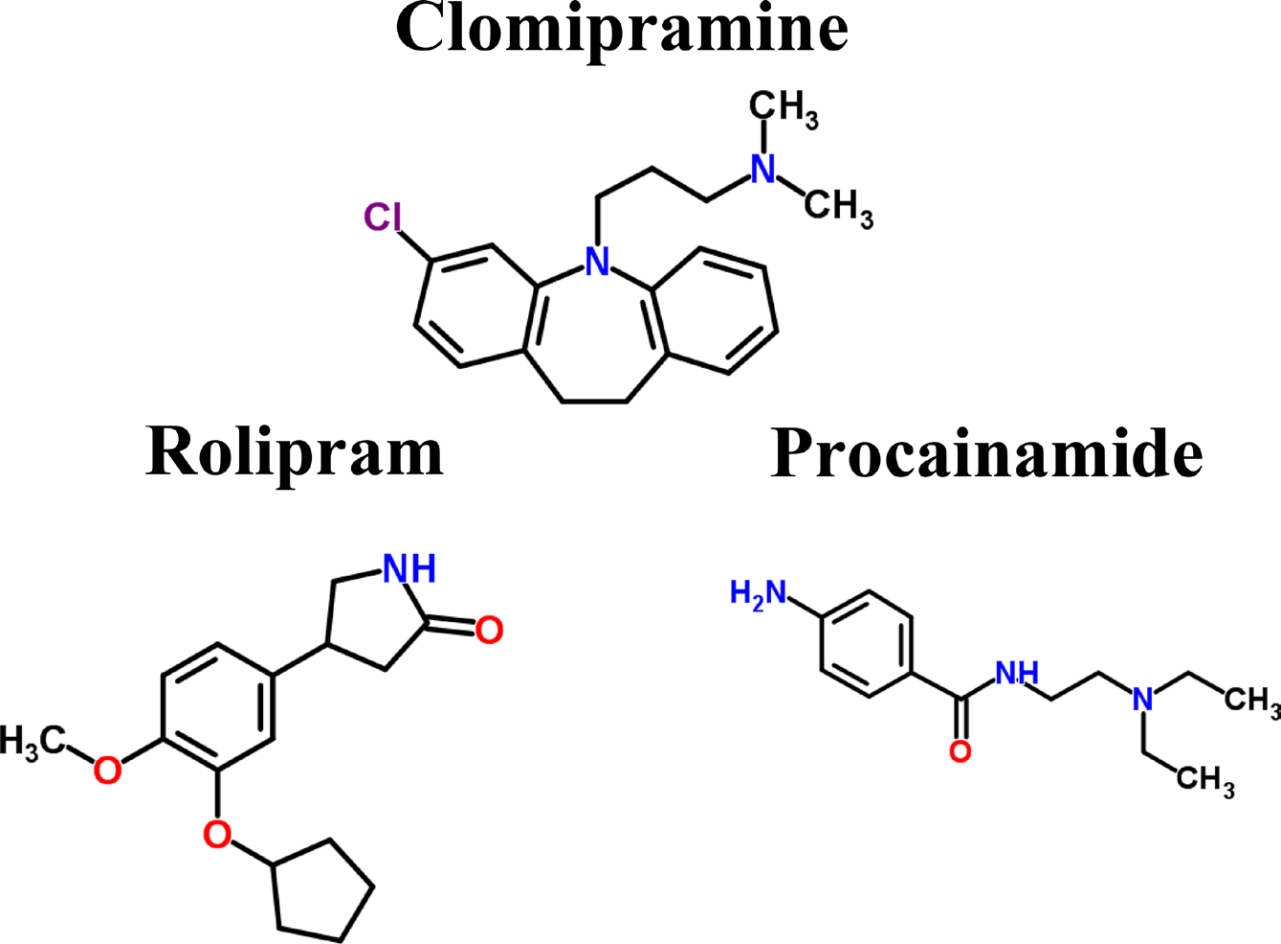

Through therapeutic treatment using the proposed genetic and epigenetic multiple-molecule drug, the proteins with significantly higher expression in the advanced stage (CDH3, CHIC2, TUBB3, LIN7C, and YBX1) can be repressed, and proteins with significant lower expression in the advanced stage (FN1, ITGA8, TGFB1, ERBB4, DDX5, EMR2, MSN, CHD3, FOXO3, and GATA3) can be activated to facilitate the restoration of advanced stage LSCC to normal lung cells.

Recently, the EGFR-tyrosine kinase inhibitor (TKI) gefitinib (Iressa) drug, which was approved by the FDA, has been used in the treatment of NSCLC [206, 207]. Previous studies reported that using gefitinib in treatment of NSCLC patients have a significant improvement in the survival of Asian patients [208, 209]. However, our sample data generally are non-Asian patients. Hence we do not consider the drug gefitinib in our designed genetic and epigenetic multiple-molecular drugs. Moreover, The drug pemetrexed, approved by the FDA, is an antifolate inhibiting multiple enzymes involved in both pyrimidine and purine synthesis. It has been reported that pemetrexed was also used in the treatment of NSCLC patients [210, 211]. However, we did not consider pemetrexed to be our designed genetic and epigenetic multiple-molecular drugs since it does not include in the 1327 compounds (i.e. drugs) of CMap database.

In conclusion, as shown in Tables 1–6, the six designed genetic and epigenetic multiple-molecular drugs for different stages of LADC and LSCC are given in the following. (1) Hydralazine, ketoconazole, and promethazine are used to repress SLC22A18, TERT, RET, POLR2D, and E2F1 and activate PDGFB, EGFL7, S100A10, and PDGFRA; (2) betulin, nordihydroguaiaretic acid, and proadifen are utilized to repress HP, LTA, APH1A, MMP8, HIF1A, and PRDM14 and activate NOTCH1, HGF, ITGA4, and VIM; (3) iloprost, methotrexate, and MK-886 are designed to repress EGF, CDH2, LOXL2, XRCC4, CNIH4, and MTOR and activate BGN, ITGB2, EPOR, IQGAP1, FN1, RHOB, TSGA10, AR, and STAT3 for the therapeutic treatment of early, middle, and advanced stage of LADC, respectively. Furthermore, (1) pimozide, mepacrine, and repaglinide are used to repress COL3A1, DDR1, CHRNA5, RPL30, CIAPIN1, GSK3B, IGF2BP3, SLC6A11, ERCC1, SUSD4, TERT, SHOX2, YBX1, SP1, and TP63 and activate FGF2, FGFR1, PDGFB, PDGFRB, FKBP5, DAPK3, and ETS1; (2) ribavirin, procainamide, and ketoconazole are utilized to repress COL1A1, JAG1, IGF1, IGF1R, TRAF4, SUSD4, and PARG and activate TNS1, ACTA2, CCL21, CCR7, MYLK, ITGB1, TSC2, GATA2, JUN, ETS1, and JUNB; (3) clomipramine, rolipram, and procainamide are designed to repress CDH3, CHIC2, TUBB3, LIN7C, and YBX1 and activate FN1, ITGA8, TGFB1, ERBB4, DDX5, EMR2, MSN, CHD3, FOXO3, and GATA3 for the therapeutic treatment of early, middle, and advanced stage of LSCC, respectively.

## MATERIALS AND METHODS

### Overview for the construction of genome-wide GENs, core GENs, and core pathways of each progression stage in LADC and LSCC

To investigate and compare the progression molecular mechanisms between LADC and LSCC from each progression stage (normal stage to early stage, early stage to middle stage, middle stage to advanced stage), we identified genome-wide real GENs and extracted core pathways among the different stages (normal lung cells, early stage, middle stage, and advanced stage) of LADC and LSCC, respectively. The steps for constructing genome-wide real GENs, core GENs, and core pathways between LADC and LSCC from each progression stage are shown in Figure 1. The procedures of constructing core GENs can be divided to four steps: (1) Big databases mining and data preprocessing of gene/miRNA/lncRNA expression data and DNA methylation data; (2) Constructing candidate GENs by using candidate PPI networks and candidate gene/miRNA/lncRNA regulatory networks (GRNs); (3) Applying system identification method and system order detection scheme with NGS data to obtain real GENs of each stage of LADC and LSCC, respectively; and (4) Using the principal network projection (PNP) method to extract core GENs containing core elements such as core proteins, genes, miRNAs, and lncRNAs from real GENs. By comparing the projection value of each core element in the core GENs, we extracted differential core signaling pathways for each progression stage of LADC and LSCC. Using one-way analysis of variance on former and later stage gene expression profile, we also could find out the elements having significant difference with *p*-value < 0.05 in the core signaling pathways from each progression stage. In the meanwhile, these core signaling pathways are not only with epigenetic modifications but also in KEGG pathway representation giving us insight into the genetic and epigenetic mechanisms of carcinogenic development in LADC and LSCC. Furthermore, we selected potential biomarkers as drugtargets based on our results for designing genetic and epigenetic multiple drugs via mining Cmap drug database for the therapeutic treatment of early stage, middle stage, and advanced stage in LADC and LSCC, respectively.

### Big data mining and data preprocessing of NGS data and methylation data for constructing GENs

In this study, we downloaded the genome-wide mRNA, miRNA, and lncRNA NGS data and the DNA methylation profiles from Cancer Brower website (https://genome-cancer.ucsc.edu/). Two datasets were considered. The one was lung cancer exon expression dataset by RNAseq including genome-wide mRNA, miRNA, and lncRNA expression NGS data. The other one was lung cancer DNA methylation profiles. Lung cancer exon expression dataset was combined from TCGA lung squamous cell carcinoma and lung adenocarcinoma datasets. This exon expression profile was measured experimentally using the Illumina HiSeq 2000 RNA Sequencing platform by the University of North Carolina TCGA genome characterization center from 1124 patients (samples). Lung cancer DNA methylation profiles were combined from TCGA lung squamous cell carcinoma and lung adenocarcinoma datasets. This DNA methylation profile was measured experimentally using the Illumina Infinium HumanMethylation450 platform and the beta values were derived at the Johns Hopkins University and University of Southern California TCGA genome characterization center from 907 patients (samples). Because the GENs are constructed by system biology approach based on gene expression level, we averaged each exon expression value, which was derived from the same gene to get the gene expression profile. Based on histopathological types, sample data are subdivided into two major histological subtypes: LADC samples and LSCC samples. According to the seventh edition of the “TNM classification of malignant tumours” published by the UICC, the carcinogenesis of non-small cell lung cancer (NSCLC) can be classified as stage IA, stage IB, stage IIA, stage IIB, stage IIIA, stage IIIB, and stage IV. In this study, we grouped stage IA and stage IB as stage I, stage IIA and stage IIB as stage II, and IIIA and IIIB as stage III, and IV. We further define stage I and stage II as early stage and middle stage, respectively. Due to the fewer samples in stage III and IV, we then grouped stage III and stage IV together as the advanced stage. Hence, the previously mentioned datasets of LADC and LSCC can be divided into four sub-datasets under four conditions, including normal stage (normal lung cells), early stage, middle stage, and advanced stage, respectively. In LADC, there are 276, 122, and 110 tumor samples in early stage, middle stage, and advanced stage, respectively. There are 58 samples in normal cells adjacent to LADC considered to be normal stage of LADC. In LSCC, there are 241, 154, and 93 tumor samples in early stage, middle stage, and advanced stage, respectively. There are 51 samples in normal cells adjacent to LSCC considered to be normal stage of LSCC.

### Constructing genome-wide candidate GENs

Biological processes in different biological conditions between each sample have different significant gene expressions and PPIs. If we only consider the significant genes or proteins in samples, many personal diversity information will be ignored. In order to investigate the biological processes under different lung conditions completely (i.e. normal stage, early stage, middle stage, and advanced stage), we directly consider whole genome information through big databases mining and systems biology approaches. Here, we constructed two candidate genome-wide GENs for LADC and LSCC, respectively. By combining the candidate PPI of LADC with candidate GRN of LADC including regulations between TF/miRNA/lncRNA and their target genes/miRNAs/lncRNAs, we obtained candidate genome-wide GENs of LADC. The same concept could be used on constructing the candidate genome-wide GEN of LSCC.

The candidate protein-protein interactions (PPIs) were extracted from the databases, including DIP [212], BIND [213], physical interaction part of Biological General Repository for Interaction Datasets (BIOGRID) [214], IntAct [215], and MINT [216]. Besides, the candidate GRNs having transcriptional regulation between transcription factors (TFs) and their target genes extracted from the databases, including Integrated Transcription Factor Platform (ITFP) [217], the Human Transcriptional Regulation Interactions database (HTRIdb) [218], and the candidate TRANScription FACtor database (TRANSFAC) [219]. Moreover, we also obtained the candidate post-transcriptional regulations between miRNAs and target genes from the database TargetScanHuman [220], including all possible targets of miRNAs and regulations between lncRNAs and their target genes from the databases, including CircuitDB [221] and StarBase2.0 [222]. The databases totally contain 4,825,453 candidate interaction pairs between proteins and proteins, 143,707 candidate regulations between TFs and genes, 2,078 candidate regulations between TFs and miRNAs, 302 candidate regulations between TFs and lncRNAs, and 229,620 candidate post-transcriptional regulations between miRNAs and genes, 50 candidate regulations between miRNAs and miRNAs, 700 candidate regulations between miRNAs and lncRNAs, and 374 candidate regulations between lncRNAs and genes (Supplementary Table 3).

### Constructing stochastic regression models of candidate PPIN and candidate GRN for candidate GENs in LADC and LSCC

In previous section, based on big data mining, we have constructed the genome-wide candidate GENs. However, many false positives are included in candidate GENs constructed from numerous databases and experimental datasets, which may contain some plausible information. Therefore, we have to prune these false positives in candidate GENs based on gene/miRNA/lncRNA expression data and DNA methylation profiles of each lung condition. In this section, we firstly constructed the stochastic regression interactive/regulatory models of human cells to characterize the interactions and regulations in candidate GENs, including PPIs, gene regulations, miRNA regulations, and lncRNA regulations, and epigenetic regulations by DNA methylation.

To identify the real GENs of each lung condition, we applied system identification and system order detection to the interactive/regulatory models of candidate GENs by using gene/miRNA/lncRNA expression data and DNA methylation profiles of each lung condition. The significant interactions and regulations out of system order will be considered as false positives in candidate GENs to be pruned to obtain real GENs for LADC and LSCC.

The stochastic regression protein interactive model of the candidate PPIN in candidate GEN, the protein interactions of the *j*th protein in lung cells for sample *n* as given by the following:

$$y_{j}[n]=\sum_{\begin{array}{l} g=1\\ j\neq g \end{array}}^{J_{j}}\alpha_{jg}y_{g}[n]y_{j}[n]+b_{j}+v_{j}[n],$$ (1)

for *j*=1, ...,*J* and *n*=1, ...,*N*

where *y_j_*[*n*] and *y_g_*[*n*] denote the expression level of the *j*-th protein and the *g*-th protein for the *n*-th sample, respectively; α_*jg*_ is the interaction ability between the *j*-th protein and the *g*-th protein, which is interactive protein of the *j*-th protein; *J_j_* represents the number of proteins interacting with the *j*-th protein and *J* is number of protein with candidate PPIN; *N* denotes the number of data samples. *b_j_* represents the basal level of protein *j*. *v_j_* [*n*] is the stochastic noise of the *j*-th protein for the sample *n* due to model uncertainty and data noise. The meaning of protein interactions model equation (1) can be explained that the expression level of the *j*-th protein is related to the interactions with *J_j_* other proteins in the candidate PPIN.

The gene regulatory model of candidate GRN, describing the transcriptional regulation of the *i*-th gene of lung cells for sample *n*, is given by the following:

$$\begin{array}{l} x_{i}[n]=\sum_{\begin{array}{l} j=1\\ i\neq j \end{array}}^{J_{i}}\beta_{ij}y_{j}[n]M_{i}[n]+\sum_{q=1}^{Q_{i}}\tau_{iq}l_{q}[n]M_{i}[n]\\ -\sum_{p=1}^{P_{i}}\delta_{ip}x_{i}[n]r_{p}[n]M_{i}[n]+k_{i}M_{i}[n]+\varepsilon_{i}[n]\; \end{array}$$ (2)

for *i*=1, ...,*I* and *n*=1, ...,*N*

where *x_i_*[*n*], *y_j_*[*n*], *l_q_*[n] and *r_p_* [*n*] denote the expression level of the *i*-th target gene, the *j*-th TF, *q*-th lncRNA and *p*-th miRNA for the *n*-th sample, respectively; *β_**ij**_* and *τ_iq_* are the transcription regulatory ability of the *j*-th TF and the *q*-th lncRNA on their corresponding binding target gene *i*, respectively. δ_*ip*_ indicates the post-transcriptional regulatory ability of the *p*-th miRNA to inhibit the *i*-th target gene (-*δ_ip_ ≤ 0*). *J_i_*, *Q_i_* and *P_i_* represent the number of TFs, lncRNAs and miRNAs binding to the *i*-th target gene, respectively; *I* and *N* denotes the number of genes with candidate GRN and the number of data samples, respectively. *k_i_* represents the basal level of target gene *i*. ε_*i*_ [*n*] is the stochastic noise of the *i*-th target gene for the sample *n* due to model uncertainty and data noise. *M_i_* [*n*] denotes the methylation regulation of the *i*-th gene through its effect on the binding affinities of TFs, miRNAs, lncRNAs, and RNA polymerase on the target gene [27, 223, 224]. The terms *β_**ij**_ y_j_*[*n*]*M_i_*[*n*], *δ_ip_x_i_*[*n*]*r_p_*[*n*]*M_i_*[*n*], *τ_iq_l_q_*[n] *M_i_*[*n*], *k_i_M_i_*[*n*] denote the effect of methylation on the binding affinities of TFs, miRNAs, lncRNAs, and RNA polymerase to the *i*-th target gene, respectively. The methylation regulation of the *i*-th target gene *M_i_* [*n*] by DNA methylation profile *m_i_*[*n*] can be defined as follows [216]:

$$M_{i}[n]=\frac{1}{1+{\left(\frac{m_{i}[n]}{0.5}\right)}^{2}}$$ (3)

where *m_i_*[*n*] indicates the DNA methylation profile of the *i*-th gene for the sample *n*. In equation (3), we can find that the range of effect of DNA methylation on target gene *i* *M_i_* [*n*] is 1 to 0.2 while DNA methylation profile *m_i_*[*n*] range from 0 to1. From the biological viewpoint, the meaning of equation (3) is that the higher DNA methylation level, the weaker binding affinity between TFs, miRNAs, lncRNAs, and RNA polymerase and their target gene. In contrast, the lower DNA methylation level, the stronger binding affinity between TFs, miRNAs, lncRNAs, and RNA polymerase and their target genes. Besides, the methylation regulation *M_i_* [*n*] in equation (3) still has regulation value (*M_i_* [*n*] = 0.2) while DNA methylation profile is 1, representing the bindings of TFs, miRNAs, lncRNAs and RNA polymerase to the *i*-th target gene are still exist. The expression level of the *i*-th gene results from transcriptional regulations of TFs and lncRNAs, the post-transcriptional regulations of miRNAs, and expression level of basal level of *i*-th gene with stochastic noise. Besides, the transcriptional regulations of TFs and lncRNAs, the post-transcriptional regulations of miRNAs and expression level of basal level (binding of RNA polymerase) can be affected by DNA methylation.

The miRNA regulatory model of candidate GRN, describing the transcriptional expression of the *p*-th miRNA of lung cells for sample *n*, is given by the following:

$$\begin{array}{l} r_{p}[n]=\sum_{\begin{array}{l} j=1\\ p\neq j \end{array}}^{J_{p}}\lambda_{pj}y_{j}[n]M_{p}[n]-\\ \sum_{z=1}^{P_{p}}\psi_{pz}r_{p}[n]r_{z}[n]M_{p}[n]+e_{p}M_{p}[n]+\omega_{p}[n], \end{array}$$ (4)

for *p* = 1, ...,*P* and *n* = 1, ...,*N*

where *r_p_*[*n*], *y_j_*[*n*], and *r_z_*[n] denote the expression level of the *p*-th miRNA, the *j*-th TF and the *z*-th miRNA for the *n*-th sample, respectively; *λ_pj_* and *ψ_pz_* are the transcription regulatory ability of the *j*-th TF on miRNA *p* and the post-transcriptional regulatory ability of the *p*-th miRNA to inhibit the *p*-th miRNA (-*ψ_pz_ ≤ 0*). *J_p_* represents the number of TF binding to the *p*-th target miRNA; *P_p_* denotes the number of miRNAs binding to the *p*-th target miRNA and the number of miRNAs with candidate GRN. *P* denotes the number of miRNA in the candidate GRN. *N* indicates the number of data samples. *e_p_* represents the basal level of target miRNA *p*. ω_*p*_[*n*] is the stochastic noise of the *p*-th target miRNA for the sample *n* due to model uncertainty and data noise. *M_p_* [*n*] denotes methylation regulation of the *p*-th miRNA as shown in equation (3) and *M_p_* [*n*] has the effect on the binding affinities of TFs, miRNAs, and RNA polymerase on the target miRNA. The terms λ_*pjyj*_[*n*]*M_p_*[*n*], *ψ_pz_r_p_*[*n*]*r_z_*[*n*]*M_p_*[*n*], *e_p_M_i_*[*n*] can express the effect of methylation on the binding affinities of TFs, miRNAs, and RNA polymerase to *p*-th target miRNA, respectively.

Moreover, the lncRNA regulatory model of candidate GRN, describing the transcriptional expression level of the *q*-th lncRNA of lung cells for sample *n*, is given by the following:

$$\begin{array}{l} l_{q}[n]=\sum_{\begin{array}{l} j=1\\ q\neq j \end{array}}^{J_{q}}\gamma_{qj}y_{j}[n]M_{q}[n]-\\ \sum_{p=1}^{P_{q}}\zeta_{qp}l_{q}[n]r_{p}[n]M_{q}[n]+f_{q}M_{q}[n]+\eta_{q}[n], \end{array}$$ (5)

for *q*=1, ...,*Q* and *n*=1, ...,*N*

where *l_q_*[*n*], *y_j_*[*n*], and *r_p_* [*n*] denote the expression level of the *q*-th target lncRNA, the *j*-th TF and *p*-th miRNA for the *n*-th sample, respectively; γ_*qj*_ and *ζ_qp_* are the transcription regulatory ability of the *j*-th TF on binding lncRNA *q* and the post-transcriptional regulatory ability of the *p*-th miRNA to inhibit the *q*-th lncRNA (-*ζ_qp_ ≤ 0*). *J_q_* and *P_q_* represent the number of TFs and miRNAs binding to the *q*-th lncRNA, respectively; *Q* and *N* denotes the number of lncRNAs in candidate GRN and the number of data samples. *f_q_* represents the basal level of lncRNA *q*. *η_g_*[*n*] is the stochastic noise of the *q*-th target lncRNA for the sample *n* due to model uncertainty and data noise. *M_q_* [*n*] denotes methylation regulation of the *q*-th lncRNA as shown in equation (3) and *M_q_* [*n*] has the effect on the binding affinities of TFs, miRNAs, and RNA polymerase on the target lncRNA. The terms *γ_qj_y_j_*[*n*]*M_q_*[*n*], *ζ_qp_l_q_*[*n*]*r_p_*[*n*]*M_q_*[*n*], *f_q_M_q_*[*n*] can express the effect of methylation on the binding affinities of TFs, miRNAs, and RNA polymerase to *q*-th target lncRNA, respectively.

### Reversed engineering and principal network projection methods to extract differential core pathways in LADC and LSCC

After constructing the protein interactive regression model (1) of candidate PPIN and gene/miRNA/lncRNA regulatory models (2), (4), and (5) of candidate GRN, we applied system identification method to do parameter estimation getting the protein interactive parameters α_*jg*_, *b_j_* in protein interactive model and gene/miRNA/lncRNA regulatory parameters *β_**ij**_*, *δ_ip_*, *τ_iq_, k_i_*, *λ_pj_*, *ψ_pz_*, *e_p_*, _*γqj*_, *ζ_qp_*, *f_q_* in gene/miRNA/lncRNA regulatory models by gene/miRNA/lncRNA expression data and DNA methylation profiles of each stage of LADC and LSCC. Moreover, it is noted that there are many false-positives existing in candidate PPINs and candidate GRNs due to experimental errors. Hence, we used system order detection method to prune false-positives for obtaining real GENs of each stage of LADC and LSCC which are shown in Supplementary Figures 1, 2. The more details of system identification and system order detection methods are in Supplementary Material 1.1. However, it is still too complicated to do analysis on identified GENs. By utilizing principal network projection method in each stage of identified GENs of LADC and LSCC, the core elements could be extracted and kept. In other words, the higher the projection value is, the more important to element contributing to the core GEN. Therefore, comparing the projection value of each element, we constructed core pathways in each stage of LADC and LSCC in respect of KEGG pathway with epigenetic modifications. Based on systems biology approaches mentioned above, we could do further genetic and epigenetic genome-wide analysis on progression molecular mechanisms between LADC and LSCC in the perspective of systematic viewpoint. Detailed information about principal network projection method could be found in Supplementary Material 1.2.

## CONCLUSIONS

In this study, based on mRNA/miRNA/lncRNA and DNA methylation profiles in NGS data, we respectively constructed the GENs for normal stage, early stage, middle stage, and advanced stage of LADC and LSCC to compare the differential genetic and epigenetic progression mechanisms between LADC and LSCC via big data mining, system identification, and system order detection methods. However, the real GENs are still too complex. We used PNP method to obtain core GENs for each stage of LADC and LSCC, respectively. By comparing core GENs among the different stages of LADC and LSCC and with the denotation of KEGG pathways, we further had core pathways in LADC and LSCC between normal lung cells to early stage, early stage to middle stage and middle stage to advanced stage to explore the differential molecular mechanisms between LADC and LSCC. Finally, we investigated how the microenvironment changes, epigenetic modifications, miRNA regulations, and lncRNA regulations to affect the differential progression molecular mechanisms between LADC and LSCC. In addition, some significant drug targets are selected from identified network biomarkers based on proteins with significant expression change between later stage and normal stage, we proposed six designed genetic and epigenetic multiple-molecular drugs for therapeutic treatment of early stage, middle stage, and advanced stage LADC and LSCC, respectively. The six designed genetic and epigenetic multiple-molecular drugs are (1) hydralazine, ketoconazole, and promethazine to target SLC22A18, TERT, RET, POLR2D, E2F1, PDGFB, EGFL7, S100A10, PDGFRA, TLR4, CCL2, RB1, TGFB1, PER1, SFTPA2, and MYC for treating early stage LADC; (2) betulin, nordihydroguaiaretic acid, and proadifen to target HP, LTA, APH1A, MMP8, HIF1A, PRDM14, NOTCH1, HGF, ITGA4, VIM, MYH9, ETS1, and ZEB1 for treating middle stage LADC; (3) iloprost, methotrexate, and MK-886 to target EGF, CDH2, LOXL2, XRCC4, CNIH4, MTOR, BGN, ITGB2, EPOR, IQGAP1, FN1, RHOB, TSGA10, AR, and STAT3 for treating advanced stage LADC; (4) pimozide, mepacrine, and repaglinide to target COL3A1, DDR1, CHRNA5, RPL30, CIAPIN1, GSK3B, IGF2BP3, SLC6A11, ERCC1, SUSD4, TERT, SHOX2, YBX1, SP1, TP63, FGF2, FGFR1, PDGFB, PDGFRB, FKBP5, DAPK3, and ETS1 for treating early stage LSCC; (5) ribavirin, procainamide, and ketoconazole to target COL1A1, JAG1, IGF1, IGF1R, TRAF4, SUSD4, PARG, TNS1, ACTA2, CCL21, CCR7, MYLK, ITGB1, TSC2, GATA2, JUN, ETS1, and JUNB for treating middle stage LSCC; (6) clomipramine, rolipram, and rolipram to target CDH3, CHIC2, TUBB3, LIN7C, YBX1, FN1, ITGA8, TGFB1, ERBB4, DDX5, EMR2, MSN, CHD3, FOXO3, and GATA3 for treating advanced stage LSCC, respectively.

## SUPPLEMENTARY MATERIALS


