# Supplementary material for: Comparing progression molecular mechanisms between lung adenocarcinoma and lung squamous cell carcinoma based on genetic and epigenetic networks: big data mining and genome-wide systems identification
Source: Oncotarget. 2019 Jun 4;10(38):3760–806. doi: 10.18632/oncotarget.26940 (PMC6557199; doi:10.18632/oncotarget.26940)
Supplement: Supplementary file 1 [file oncotarget-10-3760-s001.pdf]

Comparing progression molecular mechanisms between lung adenocarcinoma and lung squamous cell carcinoma based on genetic and epigenetic networks: big data mining and genome-wide systems identification

SUPPLEMENTARY MATERIALS

1.1 Parameter estimation of the stochastic regression models of candidate GENs via system identification method and system order detection

To identify the parameters in equations (1), (2), (4), (5) in the main article, these equations can be rewritten in the following regression forms:

$$y_j[n] = \begin{bmatrix} y_j[n]y_1[n] & \cdots & y_j[n]y_{J_j}[n] & 1 \end{bmatrix} \times \begin{bmatrix} \alpha_{j1} \\ \vdots \\ \alpha_{jJ_j} \\ b_j \end{bmatrix} + v_j[n] \quad (1)$$

$$x_i[n] = \begin{bmatrix} y_1[n]M_i[n] & \cdots & y_{J_i}[n]M_i[n] & l_1[n]M_i[n] & \cdots & l_{Q_i}[n]M_i[n] & x_i[n]r_1[n]M_i[n] & \cdots & x_i[n]r_{P_i}[n]M_i[n] & M_i[n] \end{bmatrix} \times \begin{bmatrix} \beta_{i1} \\ \vdots \\ \beta_{iJ_i} \\ \tau_{i1} \\ \vdots \\ \tau_{iQ_i} \\ -\delta_{i1} \\ \vdots \\ -\delta_{iP_i} \\ k_i \end{bmatrix} + \varepsilon_i[n] \quad (2)$$

$$r_p[n] = \begin{bmatrix} y_1[n]M_p[n] & \cdots & y_{J_p}[n]M_p[n] & r_p[n]r_1[n]M_p[n] & \cdots & r_p[n]r_{P_p}[n]M_p[n] & M_p[n] \end{bmatrix} \times \begin{bmatrix} \lambda_{p1} \\ \vdots \\ \lambda_{pJ_p} \\ -\psi_{p1} \\ \vdots \\ -\psi_{pP_p} \\ e_p \end{bmatrix} + \omega_p[n] \quad (3)$$

$$l_q[n] = \Big[y_1[n] M_q[n] \quad \cdots \quad y_{J_q}[n] M_q[n] \quad l_q[n] r_1[n] M_q[n] \quad \cdots \quad l_q[n] r_{P_q}[n] M_q[n] \quad M_q[n] \Big] \cdot \begin{bmatrix} \gamma_{q1} \\ \vdots \\ \gamma_{qJ_q} \\ -\zeta_{q1} \\ \vdots \\ -\zeta_{qP_q} \\ f_q \end{bmatrix} + \eta_q[n] \tag{4}$$

which can be simply represented as follows, respectively:

$$y_j[n] = \phi_{j,P}[n] \cdot \theta_{j,P} + v_j[n] \text{, for j=1, ...,J and n=1, ...,N} \tag{5}$$

$$x_i[n] = \phi_{i,G}[n] \cdot \theta_{i,G} + \varepsilon_i[n] \text{, for i=1, ...,I and n=1, ...,N} \tag{6}$$

$$r_p[n] = \phi_{p,M}[n] \cdot \theta_{p,M} + \omega_p[n] \text{, for p=1, ...,P and n=1, ...,N} \tag{7}$$

$$l_q[n] = \phi_{q,L}[n] \cdot \theta_{q,L} + \eta_q[n] \text{, for q=1, ...,Q and n=1, ...,N} \tag{8}$$

where $\phi_{j,P}$[n], $\phi_{i,G}$[n], $\phi_{\mathrm{p,M}}$[n], and $\phi_{q,L}$[n] denote the regression vector for sample *n*, comprising protein/gene/miRNA/lncRNA expression data and DNA methylation profiles. $\theta_{j,P}$, $\theta_{i,G}$, $\theta_{\mathrm{p,M}}$, and $\theta_{q,L}$ are the parameter vectors associated with the protein interactions and gene/miRNA/lncRNA regulations, respectively, containing protein interaction abilities, transcriptional regulatory abilities, post-transcriptional regulatory abilities, and basal levels.

Besides, for all *N* samples, the regression forms in (10), (11), (12), (13) at different samples can be augmented as follows, respectively:

$$\begin{bmatrix} y_j[1] \\ y_j[2] \\ \vdots \\ y_j[N] \end{bmatrix} = \begin{bmatrix} \phi_{j,P}[1] \\ \phi_{j,P}[2] \\ \vdots \\ \phi_{j,P}[N] \end{bmatrix} \theta_{j,P} + \begin{bmatrix} v_j[1] \\ v_j[2] \\ \vdots \\ v_j[N] \end{bmatrix} \tag{9}$$

$$\begin{bmatrix} x_i[1] \\ x_i[2] \\ \vdots \\ x_i[N] \end{bmatrix} = \begin{bmatrix} \phi_{i,G}[1] \\ \phi_{i,G}[2] \\ \vdots \\ \phi_{i,G}[N] \end{bmatrix} \theta_{i,G} + \begin{bmatrix} \varepsilon_i[1] \\ \varepsilon_i[2] \\ \vdots \\ \varepsilon_i[N] \end{bmatrix} \tag{10}$$

$$\begin{bmatrix} r_p[1] \\ r_p[2] \\ \vdots \\ r_p[N] \end{bmatrix} = \begin{bmatrix} \phi_{p,M}[1] \\ \phi_{p,M}[2] \\ \vdots \\ \phi_{p,M}[N] \end{bmatrix} \theta_{p,M} + \begin{bmatrix} \omega_p[1] \\ \omega_p[2] \\ \vdots \\ \omega_p[N] \end{bmatrix} \tag{11}$$

$$\begin{bmatrix} l_q[1] \\ l_q[2] \\ \vdots \\ l_q[N] \end{bmatrix} = \begin{bmatrix} \phi_{q,L}[1] \\ \phi_{q,L}[2] \\ \vdots \\ \phi_{q,L}[N] \end{bmatrix} \theta_{q,L} + \begin{bmatrix} \eta_q[1] \\ \eta_q[2] \\ \vdots \\ \eta_q[N] \end{bmatrix} \tag{12}$$

which can be also simply represented as follows, respectively:

$$Y_j = \Phi_{j,P} \cdot \theta_{j,P} + W_{j,P} \tag{13}$$

$$X_i = \Phi_{i,G} \cdot \theta_{i,G} + W_{i,G} \tag{14}$$

$$R_p = \Phi_{p,M} \cdot \theta_{p,M} + W_{p,M} \tag{15}$$

$$L_q = \Phi_{q,L} \cdot \theta_{q,L} + W_{q,L} \tag{16}$$

Hence we can use the constrained least square estimation to estimate the parameter vectors θ_j,P, θ_i,G, θ_p,M, θ_q,L, containing protein interaction abilities, transcriptional regulatory abilities, post-transcriptional regulatory abilities, and basal levels by the corresponding NGS data and DNA methylation profiles. The constrained least square estimation problem of parameter vectors $\theta_{j,P}$, $\theta_{i,G}$, $\theta_{p,M}$, $\theta_{q,L}$ in equation (18), (19), (20), (21) can be solved by the following form, respectively:

$$\min_{\theta_{j,P}} \frac{1}{2} \left\| \Phi_{j,P} \cdot \theta_{j,P} - Y_j \right\|_2^2 \tag{17}$$

$$\min_{\theta_{i,G}} \frac{1}{2} \left\| \Phi_{i,G} \cdot \theta_{i,G} - X_i \right\|_2^2 \tag{18}$$

$$\text{subject to } \begin{bmatrix} \overbrace{\begin{matrix} 0 & \cdots & 0 \end{matrix}}^{J_i} & \overbrace{\begin{matrix} 0 & \cdots & 0 \end{matrix}}^{Q_i} & \overbrace{\begin{matrix} 1 & \cdots & 0 \end{matrix}}^{P_i} & 0 \\ \begin{matrix} \vdots & \ddots & \vdots \end{matrix} & \begin{matrix} \vdots & \ddots & \vdots \end{matrix} & \begin{matrix} \vdots & \ddots & \vdots \end{matrix} & \vdots \\ \begin{matrix} 0 & \cdots & 0 \end{matrix} & \begin{matrix} 0 & \cdots & 0 \end{matrix} & \begin{matrix} 0 & \cdots & 1 \end{matrix} & 0 \end{bmatrix} \theta_{i,G} \le \begin{bmatrix} 0 \\ \vdots \\ 0 \end{bmatrix}$$

$$\min_{\theta_{p,M}} \frac{1}{2} \left\| \Phi_{p,M} \cdot \theta_{p,M} - R_p \right\|_2^2 \tag{19}$$

$$\text{subject to } \begin{bmatrix} \overbrace{\begin{matrix} 0 & \cdots & 0 \end{matrix}}^{J_p} & \overbrace{\begin{matrix} 1 & \cdots & 0 \end{matrix}}^{P_p} & 0 \\ \begin{matrix} \vdots & \ddots & \vdots \end{matrix} & \begin{matrix} \vdots & \ddots & \vdots \end{matrix} & \vdots \\ \begin{matrix} 0 & \cdots & 0 \end{matrix} & \begin{matrix} 0 & \cdots & 1 \end{matrix} & 0 \end{bmatrix} \theta_{p,M} \le \begin{bmatrix} 0 \\ \vdots \\ 0 \end{bmatrix}$$

$$\min_{\theta_{q,L}} \frac{1}{2} \left\| \Phi_{q,L} \cdot \theta_{q,L} - L_q \right\|_2^2 \tag{20}$$

$$\text{subject to } \begin{bmatrix} \overbrace{\begin{matrix} 0 & \cdots & 0 \end{matrix}}^{J_q} & \overbrace{\begin{matrix} 1 & \cdots & 0 \end{matrix}}^{P_q} & 0 \\ \begin{matrix} \vdots & \ddots & \vdots \end{matrix} & \begin{matrix} \vdots & \ddots & \vdots \end{matrix} & \vdots \\ \begin{matrix} 0 & \cdots & 0 \end{matrix} & \begin{matrix} 0 & \cdots & 1 \end{matrix} & 0 \end{bmatrix} \theta_{q,L} \le \begin{bmatrix} 0 \\ \vdots \\ 0 \end{bmatrix}$$

The inequality constraints in the above constrained least square parameter estimation problem can ensure that the post-transcriptional regulatory abilities of miRNA on genes/miRNAs/lncRNAs are always non-positive. Therefore, we could solve the constrained least square parameter estimation problem to obtain protein interactive parameters, i.e., $\hat{\theta}_{j,P}$ and gene/miRNA/lncRNA regulatory parameters, i.e., $\hat{\theta}_{i,G}$, $\hat{\theta}_{p,M}$, and $\hat{\theta}_{q,L}$ through the system identification method in MATLAB optimization toolbox based on a reflective Newton method for minimizing a quadratic function [1].

Due to large-scale measurement of expressed cellular proteins has not been realized yet and mRNA abundance can explain 73% of the variance in protein abundance [2], it allows us to use NGS of gene expressions to substitute protein expressions. Thus we can use the gene, miRNA, and lncRNA expression data and DNA methylation profiles of each stage (normal stage, early stage, middle stage, and advanced stage) in LADC and LSCC, which are obtained from the Cancer Brower website (https://genome-cancer.ucsc.edu), to construct Y_j, X_i, R_p, L_q, $\Phi_{j,P}$, $\Phi_{i,G}$, $\Phi_{p,M}$, and $\Phi_{q,l}$ in (22), (23), (24) and (25) to identify the protein interactive abilities, transcriptional regulatory abilities, post-transcriptional regulatory abilities and basal levels in $\theta_{j,P}$, $\theta_{i,G}$, $\theta_{p,M}$, and $\theta_{q,L}$.

Since the candidate interactions and regulations were constructed by big data mining from numerous databases and experimental datasets which may contain some plausible information, it is possible that many false positives protein interactive abilities, transcriptional regulatory abilities, and post-transcriptional regulatory abilities are included. To eliminate false positives protein interactive abilities, transcriptional regulatory abilities, and post-transcriptional regulatory abilities, we applied system order detection scheme to prune out these insignificant abilities out of system order as false positives to obtain real GENs.

Akaike Information Criterion (AIC) is a system order detection method based on the identification method in solving (22), (23), (24) and (25) for detecting the real system order. The AICs of *j*-th protein of PPIN, *i*-th gene of GRN, *p*-th miRNA of GRN, and *q*-th lncRNA of GRN are shown in the following form, respectively:

$$AIC(J_j) = \log(\hat{\sigma}^2_{j,P}) + \frac{2(\Delta_{j,P})}{N} \tag{26}$$

$$AIC(J_i, Q_i, P_i) = \log(\hat{\sigma}^2_{i,G}) + \frac{2(\Delta_{i,G})}{N} \tag{27}$$

$$AIC(J_p, P_p) = \log(\hat{\sigma}^2_{p,M}) + \frac{2(\Delta_{p,M})}{N} \tag{28}$$

t

$$AIC(J_q, P_q) = \log(\hat{\sigma}^2_{q,L}) + \frac{2(\Delta_{q,L})}{N} \tag{29}$$

where $\Delta_{j,P}$ denotes the number of parameters of protein *j*, i.e., $\Delta_{j,P} = J_j+1$ in the estimation problem of protein interactive model of the PPIN in equation (1); $\Delta_{i,G}$, $\Delta_{p,M}$, and $\Delta_{q,L}$ represent the number of parameters, i.e., $\Delta_{i,G} = J_i+Q_i+P_i+1$, $\Delta_{p,M} = J_p+P_p+1$, and $\Delta_{q,L} = J_q+P_q+1$ in the estimation problem of gene *i*, miRNA *p*, and lncRNA *q* in the regulatory model of the GRN, respectively; $\hat{\sigma}^2_{j,P}$ is estimated residual error obtained from the system identification method, i.e., $\hat{\sigma}^2_{j,P} = \left(Y_j - \left(\Phi_{j,P}\hat{\theta}_{j,P}\right)\right)^T \left(Y_j - \left(\Phi_{j,P}\hat{\theta}_{j,P}\right)\right)/N$ in the estimation problem of protein interactive model of the PPIN; $\hat{\sigma}^2_{i,G}$, $\hat{\sigma}^2_{p,M}$, and $\hat{\sigma}^2_{q,L}$ is estimated residual error obtained from the system identification method, i.e., $\hat{\sigma}^2_{i,G} = \left(X_i - \left(\Phi_{i,G}\hat{\theta}_{i,G}\right)\right)^T \left(X_i - \left(\Phi_{i,G}\hat{\theta}_{i,G}\right)\right)/N$, $\hat{\sigma}^2_{p,M} = \left(R_p - \left(\Phi_{p,M}\hat{\theta}_{p,M}\right)\right)^T \left(R_p - \left(\Phi_{p,M}\hat{\theta}_{p,M}\right)\right)/N$, and $\hat{\sigma}^2_{q,L} = \left(L_q - \left(\Phi_{q,L}\hat{\theta}_{q,L}\right)\right)^T \left(L_q - \left(\Phi_{q,L}\hat{\theta}_{q,L}\right)\right)/N$ in the estimation problem of gene *i*, miRNA *p*, and lncRNA *q* in the regulatory model of the GRN, respectively; $\hat{\theta}_{j,P}$ denotes the parameters identified in the estimation problem of protein interactive model of protein *j* in the PPIN; $\hat{\theta}_{i,G}$, $\hat{\theta}_{p,M}$, and $\hat{\theta}_{q,L}$ represent the parameters identified in the estimation problem of gene *i*, miRNA *p*, and lncRNA *q* in the regulatory model of the GRN, respectively; Based on the theory of system identification [3–4], the true number of parameters $\Delta^*_{j,P}$, $\Delta^*_{i,G}$, $\Delta^*_{p,M}$, and $\Delta^*_{q,L}$ could minimize $AIC(J_j)$, $AIC(J_i,Q_i,P_i)$, $AIC(J_p,P_p)$, and $AIC(J_q,P_q)$ in (26), (27), (28), and (29) respectively to obtain the true system order of GEN. In other words, the minimum $AIC(J_j)$, $AIC(J_i,Q_i,P_i)$, $AIC(J_p,P_p)$, and $AIC(J_q,P_q)$ could be achieved by $\Delta^*_{j,P}$, $\Delta^*_{i,G}$, $\Delta^*_{p,M}$, and $\Delta^*_{q,L}$ respectively by tradeoff between residual error and parameter association number. Therefore, we could prune the false positives protein interactive abilities, transcriptional regulatory abilities, and post-transcriptional regulatory abilities in candidate GENs to obtain real GENs at each stage of LADC and LSCC through deleting the insignificant interactions and regulations out of true system order one protein by one protein, one gene by one gene, one miRNA by one miRNA, and one lncRNA by one lncRNA.

In this study, based on gene/miRNA/lncRNA expression data and DNA methylation profiles for each stage (normal stage, early stage, middle stage, and advanced stage) of LADC and LSCC, we obtained eight group of samples for the eight lung conditions with different number of samples N (i.e. NLADC,Normal=58, NLADC,eaely=276, NLADC,middle=122, NLADC,advanced=110, NLSCC,Normal=51, NLSCC,early=241, NLSCC,middle=,154 NLSCC,advanced=93). Hence we can identify eight real GENs of lung cells for each stage of LADC and LSCC, respectively (Supplementary Figures 1 and 2).

1.2 Extracting core GENs from the real GENs by using the PNP method

However, these real GENs remain large and complex. It is difficult to reveal the significant information to get an insight to the genetic and epigenetic mechanisms of progression in LADC and LSCC to allow us to compare and investigate progression

mechanisms between LADC and LSCC. Hence, we used PNP method to extract the core GENs from the real GENs in different lung conditions (i.e. normal stage, early stage, middle stage, advanced stage of LADC and LSCC). The system models, describing the PPIs, gene regulations, miRNA regulations, and lncRNA regulations, and epigenetic regulations by DNA methylation of real GENs, can be shown by the followings:

$$y_j[n] = \sum_{g \in J_j} \hat{\alpha}_{jg} y_g[n] y_j[n] + \hat{b}_j + v_j[n], \text{ for } j=1, \ldots, J \text{ and } n=1, \ldots, N \quad (25)$$

$$x_i[n] = \sum_{j \in J_i} \hat{\beta}_{ij} y_j[n] M_i[n] + \sum_{q \in Q_i} \hat{\tau}_{iq} l_q[n] M_i[n] - \sum_{p \in P_i} \hat{\delta}_{ip} x_i[n] r_p[n] M_i[n] + \hat{k}_i M_i[n] + \varepsilon_i[n], \text{ for } i=1, \ldots, I \text{ and } n=1, \ldots, N \quad (26)$$

$$r_p[n] = \sum_{j \in J_p} \hat{\lambda}_{pj} y_j[n] M_p[n] - \sum_{z \in P_p} \hat{\psi}_{pz} r_p[n] r_z[n] M_p[n] + \hat{e}_p M_p[n] + \omega_p[n], \text{ for } p=1, \ldots, P \text{ and } n=1, \ldots, N \quad (27)$$

$$l_q[n] = \sum_{j \in J_q} \hat{\gamma}_{qj} y_j[n] M_q[n] - \sum_{p \in P_q} \hat{\zeta}_{qp} l_q[n] r_p[n] M_q[n] + \hat{f}_q M_q[n] + \eta_q[n], \text{ for } q=1, \ldots, Q \text{ and } n=1, \ldots, N \quad (28)$$

where J_j, J_i, Q_i, P_i, J_p, P_p, J_q, and P_q represent the number of protein j in PPIs, TF regulations on gene i, miRNA regulations on gene i, lncRNA regulations on gene i, TF regulations on miRNA p, miRNA regulations on miRNA p, TF regulations on lncRNA q, and miRNA regulations on lncRNA q of real GENs obtained by AIC, respectively. $\hat{\alpha}_{jg}$ indicates the estimated protein interactive abilities, and $\hat{\beta}_{ij}$, $\hat{\lambda}_{pj}$, $\hat{\gamma}_{qj}$ denote the estimated transcriptional regulatory abilities of TFs on gene/miRNA/lncRNA, respectively; $\hat{\tau}_{iq}$ is the estimated transcriptional regulatory abilities of lncRNAs on gene, and $\hat{\delta}_{ip}$, $\hat{\psi}_{pz}$, $\hat{\zeta}_{qp}$ represent the estimated post-transcriptional regulatory abilities of miRNAs on gene/miRNA/lncRNA, respectively. These estimated protein interactive abilities, estimated transcriptional regulatory abilities, and estimated post-transcriptional regulatory abilities can be estimated by applying system identification method to the (22), (23), (24), (25) via the corresponding NGS data and DNA methylation profiles.

After the protein interactive abilities, transcriptional regulatory abilities, and post-transcriptional regulatory abilities were identified, we integrated all interactive abilities of proteins, transcriptional regulatory abilities of TFs and lncRNAs, and post-transcriptional regulatory abilities of miRNAs of the real GEN implicated in equation (30), (31), (32), (33) as the following network structure matrix H.

$$H = \begin{bmatrix} H_{pp} & 0 & 0 \\ H_{tg} & H_{lg} & H_{mg} \\ H_{tl} & 0 & H_{ml} \\ H_{tm} & 0 & H_{mm} \end{bmatrix} \quad (29)$$

where the sub-network structure matrix $H_{pp} = \begin{bmatrix} \hat{\alpha}_{11} & \cdots & \hat{\alpha}_{1g} & \cdots & \hat{\alpha}_{1J} \\ \vdots & \ddots & \vdots & \ddots & \vdots \\ \hat{\alpha}_{j1} & \cdots & \hat{\alpha}_{jg} & \cdots & \hat{\alpha}_{1J} \\ \vdots & \ddots & \vdots & \ddots & \vdots \\ \hat{\alpha}_{J1} & \cdots & \hat{\alpha}_{Jg} & \cdots & \hat{\alpha}_{JJ} \end{bmatrix}$ denotes the matrix associated with interactive abilities of proteins; The sub-network structure matrix $H_{tg} = \begin{bmatrix} \hat{\beta}_{11} & \cdots & \hat{\beta}_{1j} & \cdots & \hat{\beta}_{1J} \\ \vdots & \ddots & \vdots & \ddots & \vdots \\ \hat{\beta}_{i1} & \cdots & \hat{\beta}_{ij} & \cdots & \hat{\beta}_{iJ} \\ \vdots & \ddots & \vdots & \ddots & \vdots \\ \hat{\beta}_{I1} & \cdots & \hat{\beta}_{Ij} & \cdots & \hat{\beta}_{IJ} \end{bmatrix}$, $H_{tl} = \begin{bmatrix} \hat{\gamma}_{11} & \cdots & \hat{\gamma}_{1j} & \cdots & \hat{\gamma}_{1J} \\ \vdots & \ddots & \vdots & \ddots & \vdots \\ \hat{\gamma}_{q1} & \cdots & \hat{\gamma}_{qj} & \cdots & \hat{\gamma}_{qJ} \\ \vdots & \ddots & \vdots & \ddots & \vdots \\ \hat{\gamma}_{Q1} & \cdots & \hat{\gamma}_{Qj} & \cdots & \hat{\gamma}_{QJ} \end{bmatrix}$,

H_{tm}= $\begin{bmatrix} \hat{\lambda}_{11} & \cdots & \hat{\lambda}_{1j} & \cdots & \hat{\lambda}_{1J} \\ \vdots & \ddots & \vdots & \ddots & \vdots \\ \hat{\lambda}_{p1} & \cdots & \hat{\lambda}_{pj} & \cdots & \hat{\lambda}_{pJ} \\ \vdots & \ddots & \vdots & \ddots & \vdots \\ \hat{\lambda}_{P1} & \cdots & \hat{\lambda}_{Pj} & \cdots & \hat{\lambda}_{PJ} \end{bmatrix}$ represent the matrices associated with transcriptional regulatory abilities of TFs on genes, lncRNAs, and miRNAs, respectively; The sub-network structure matrix H_{mg}= $\begin{bmatrix} \hat{\delta}_{11} & \cdots & \hat{\delta}_{1p} & \cdots & \hat{\delta}_{1P} \\ \vdots & \ddots & \vdots & \ddots & \vdots \\ \hat{\delta}_{i1} & \cdots & \hat{\delta}_{ip} & \cdots & \hat{\delta}_{iP} \\ \vdots & \ddots & \vdots & \ddots & \vdots \\ \hat{\delta}_{I1} & \cdots & \hat{\delta}_{Ip} & \cdots & \hat{\delta}_{IP} \end{bmatrix}$, H_{ml}= $\begin{bmatrix} \hat{\zeta}_{11} & \cdots & \hat{\zeta}_{1p} & \cdots & \hat{\zeta}_{1P} \\ \vdots & \ddots & \vdots & \ddots & \vdots \\ \hat{\zeta}_{q1} & \cdots & \hat{\zeta}_{qp} & \cdots & \hat{\zeta}_{qP} \\ \vdots & \ddots & \vdots & \ddots & \vdots \\ \hat{\zeta}_{Q1} & \cdots & \hat{\zeta}_{Qp} & \cdots & \hat{\zeta}_{QP} \end{bmatrix}$, H_{mm}= $\begin{bmatrix} \hat{\psi}_{11} & \cdots & \hat{\psi}_{1z} & \cdots & \hat{\psi}_{1P} \\ \vdots & \ddots & \vdots & \ddots & \vdots \\ \hat{\psi}_{p1} & \cdots & \hat{\psi}_{pz} & \cdots & \hat{\psi}_{pP} \\ \vdots & \ddots & \vdots & \ddots & \vdots \\ \hat{\psi}_{P1} & \cdots & \hat{\psi}_{Pz} & \cdots & \hat{\psi}_{PP} \end{bmatrix}$ indicate the matrices associated with post-transcriptional regulatory abilities of miRNAs on genes, lncRNAs, and miRNAs, respectively; The sub-network structure matrix H_{lg}= $\begin{bmatrix} \hat{\tau}_{11} & \cdots & \tau_{1q} & \cdots & \tau_{1Q} \\ \vdots & \ddots & \vdots & \ddots & \vdots \\ \hat{\tau}_{i1} & \cdots & \tau_{iq} & \cdots & \tau_{iQ} \\ \vdots & \ddots & \vdots & \ddots & \vdots \\ \hat{\tau}_{I1} & \cdots & \tau_{Iq} & \cdots & \tau_{IQ} \end{bmatrix}$ is the matrix associated with transcriptional regulatoryt abilities of lncRNAs on genes. In the real GENs, if an interaction between any two proteins or a regulation between any two elements of TFs, genes, miRNAs, and lncRNA is disconnected, its corresponding ability will be set to zero.

Based on singular value decomposition (SVD), the network structure projection method, PNP, can be described as follows:

$$H = USV^{\mathrm{T}} \tag{30}$$

where $H \in \mathbb{R}^{(J+I+Q+P)\times(J+Q+P)}$; $U \in \mathbb{R}^{(J+I+Q+P)\times(J+Q+P)}$; $V \in \mathbb{R}^{(J+Q+P)\times(J+Q+P)}$; S is a diagonal matrix (i.e. $S = diag\left(s_1,\cdots,s_m,\cdots,s_{J+Q+P}\right)$), including J+Q+P non-negative singular values of H with descending order $s_1 \geq \cdots \geq s_m \geq \cdots \geq s_{J+Q+P} \geq 0$ and $diag\left(s_1,s_2\right)$ indicates the diagonal matrix of s_1 and s_2 (i.e., $\begin{bmatrix} s_1 & 0 \\ 0 & s_2 \end{bmatrix}$). Then the eigenexpression fraction (E_m) can be defined by the following normalization equation:

$$E_m = \frac{s_m^2}{\sum_{m=1}^{J+Q+P} s_m^2} \tag{31}$$

We selected the top M singular vectors of U and V, such that $\sum_{m=1}^{M} E_m \geq$ 0.85 with the minimal M to construct the principal network structure of 85% from the energy perspective and the projection of H to the top M singular vectors of U and V, which

means all edges of each node (i.e., each protein, gene, miRNA and lncRNA) in real GENs should be projected to the top M singular vectors of U and V, is defined as follows:

$$a_L(k,m) = h_{:,k}^{\mathrm{T}} \times u_{:,m} \text{ and } a_R(t,m) = h_{t,:} \times v_{:,m},$$

for k=1, …,$J+Q+P$, t=1, …,$J+I+Q+P$, and m=1, …,M (32)

where $h_{:,k}$ and $h_{t,:}$ represent the k-th column vector and t-th row vector of H. respectively; $u_{:,m}$ and $v_{:,m}$ denote the m-th column vectors of U and V, which are left-singular vectors and right-singular vectors of U and V, respectively, for m=1, ...,M. We further defined the 2-norm projection value of each node to the top M left-singular vectors and M right singular vectors as follows.

$$D_L(k) = \left[\sum_{m=1}^{M}\left[a_L(k,m)\right]^2\right]^{1/2} \text{ and } D_R(t) = \left[\sum_{m=1}^{M}\left[a_R(t,m)\right]^2\right]^{1/2},$$

for k=1, …,$J+Q+P$ and t=1, …,$J+I+Q+P$ (38)

Hence we can obtain the projection values of all node, including proteins, genes, miRNAs, and lncRNAs in real GENs. If the projection value $D_L(k)$ or $D_R(t)$ is close to zero, it means that the corresponding node is almost independent to the top M left-singular vectors or top M right-singular vectors. In other words, if a node of the real GEN has a higher projection value, it is more important for the principal network structure of GEN. Since the aim of this study is to compare progression genetic and epigenetic mechanisms between LADC and LSCC, we can identify the core proteins and TFs with top projection values, and then with these core proteins and TFs to connect with their miRNAs/lncRNAs to form the core GENs for further carcinogenesis investigation.

REFERENCES

1. Coleman TF, Li Y. A Reflective Newton Method for Minimizing a Quadratic Function Subject to Bounds on Some of the Variables. SIAM Journal on Optimization. 1996; 6:1040–1058. https://doi.org/10.1137/S1052623494240456.
2. Lu P, Vogel C, Wang R, Yao X, Marcotte EM. Absolute protein expression profiling estimates the relative contributions of transcriptional and translational regulation. Nat Biotechnol. 2007; 25:117–124. https://doi.org/10.1038/nbt1270. [PubMed]
3. Johansson R. (1993). System Modeling and Identification: Prentice Hall.
4. Chen LZ, Nguang SK, Chen XD. On-line identification and optimization of feed rate profiles for high productivity fed-batch culture of hybridoma cells using genetic algorithms. ISA Trans. 2002; 41:409–419. https://doi.org/10.1016/S0019-0578(07)60098-6. [PubMed]

Supplementary Table 1: The number of identified nodes and edges of real GENs in each stage of LADC

Node/Edge	Normal stage LADC (58)	Early stage LADC (276)	Middle stage LADC (122)	Advanced stage LADC (110)
R	2,462	2,609	2,586	2,571
P	19,594	19,987	19,915	19,869
P—P	987,105	2,251,921	1,869,944	1,676,563
T	1,883	2,041	1,951	1,910
T→G	68,900	70,070	65,742	63,721
T→M	1,100	1,308	997	1,157
T→L	131	142	126	114
M	357	361	353	352
M→G	33,504	37,689	27,218	26,964
M→M	5	5	4	4
M→L	97	114	69	74
L	109	125	107	95
L→G	141	191	225	183

LADC: Lung adenocarcinoma; R: receptor-associated proteins; P: Proteins; T: TFs; G: genes; M: miRNAs; L: lncRNAs; P—P: interactions between proteins and proteins; T→G: transcriptional regulations from TFs to genes; T→M: transcriptional regulations from TFs to genes of miRNAs; T→L: transcriptional regulations from TFs to genes of lncRNAs; M→G: post-transcriptional regulations on genes; M→M: post-transcriptional regulations from miRNAs to miRNAs; M→L: post-transcriptional regulations from miRNAs to lncRNAs; L→G: epigenetic regulations from lncRNAs to genes; In LADC, there are 276, 122, and 110 tumor samples in early stage, middle stage, and advanced stage, respectively. 58 samples in normal cells adjacent to LADC are considered in normal stage of LADC.

Supplementary Table 2: The number of identified nodes and edges of real GENs in each stage of LSCC

Node/Edge	Normal stage LSCC (51)	Early stage LSCC (241)	Middle stage LADC (154)	Advanced stage LADC (93)
R	2,498	2,465	2,592	2,582
P	19,688	20,062	19,945	19,897
P—P	883,271	2,242,671	2,003,177	1,500,063
T	1,843	2,018	1,956	1,909
T→G	62,930	68,149	60,834	61,693
T→M	1,247	1,314	1,046	1,172
T→L	96	133	137	108
M	360	361	348	344
M→G	35,884	37,291	27,832	24,949
M→M	9	13	7	8
M→L	58	133	91	75
L	84	113	106	97
L→G	124	199	221	176

LSCC: Lung squamous cell; R: receptor-associated proteins; P: Proteins; T: TFs; G: genes; M: miRNAs; L: lncRNAs; P—P: interactions between proteins and proteins; T→G: transcriptional regulations from TFs to genes; T→M: transcriptional regulations from TFs to genes of miRNAs; T→L: transcriptional regulations from TFs to genes of lncRNAs; M→G: post-transcriptional regulations on genes; M→M: post-transcriptional regulations from miRNAs to miRNAs; M→L: post-transcriptional regulations from miRNAs to lncRNAs; L→G: epigenetic regulations from lncRNAs to genes; In LSCC, there are 241, 154, and 93 tumor samples in early stage, middle stage, and advanced stage, respectively. 51 samples in normal cells adjacent to LSCC are also considered in normal stage of LSCC.

Supplementary Table 3: The number of nodes and edges in candidate GENs

Node/Edge	Candidate
R	2,599
P	21,305
P—P	4,825,453
T	2,638
T→G	143,707
T→M	2,078
T→L	302
M	363
M→G	229,620
M→M	50
M→L	700
L	281
L→G	374

R: receptor-associated proteins; P: Proteins; T: TFs; G: genes; M: miRNAs; L: lncRNAs; P—P: interactions between proteins and proteins; T→G: transcriptional regulations from TFs to genes; T→M: transcriptional regulations from TFs to genes of miRNAs; T→L: transcriptional regulations from TFs to genes of lncRNAs; M→G: post-transcriptional regulations on genes; M→M: post-transcriptional regulations from miRNAs to miRNAs; M→L: post-transcriptional regulations from miRNAs to lncRNAs; L→G: epigenetic regulations from lncRNAs to genes.

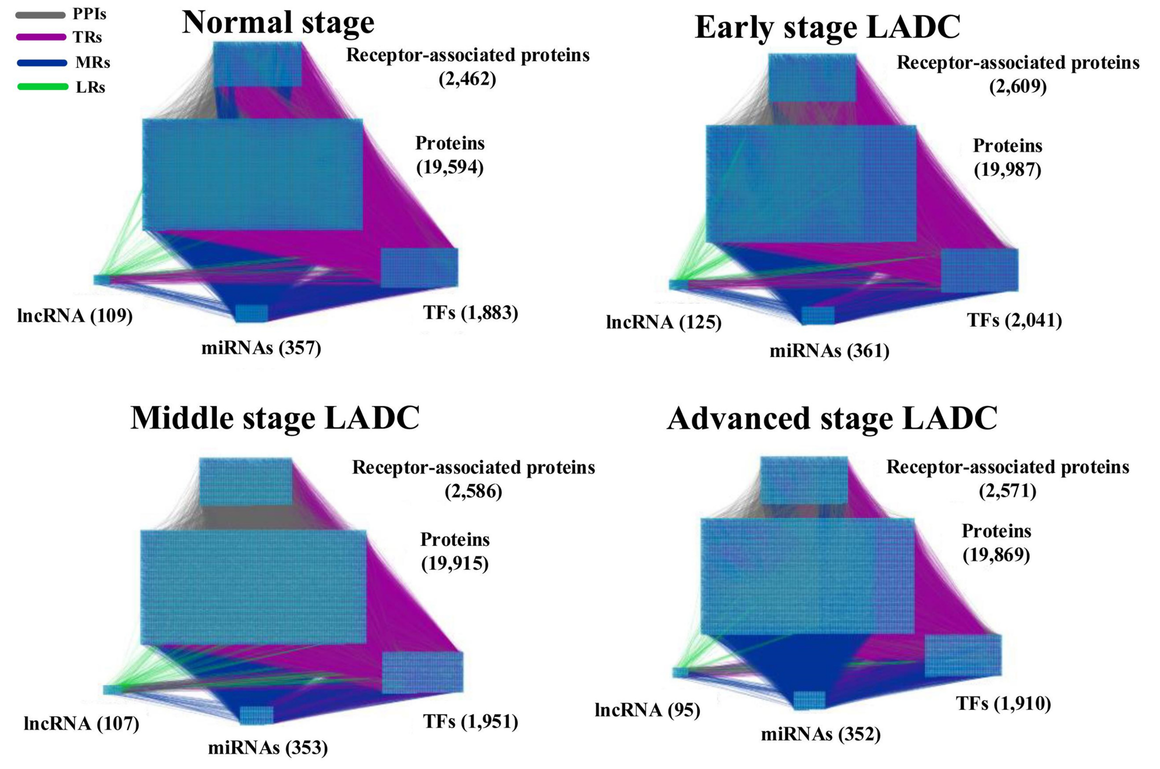

Supplementary Figure 1: The genetic and epigenetic networks (GENs) of normal stage, early stage, middle stage, and advanced stage LADC. These figures show the identified genetic and epigenetic networks (GENs) of normal lung cells, early stage LADC, middle stage LADC, and advanced stage LADC. The grey lines represent protein-protein interactions (PPIs); the purple lines indicate the transcriptional regulations (TRs); the blue lines denote the miRNA post-transcriptional regulations (MLRs); the green lines are lncRNA regulations (LRs).

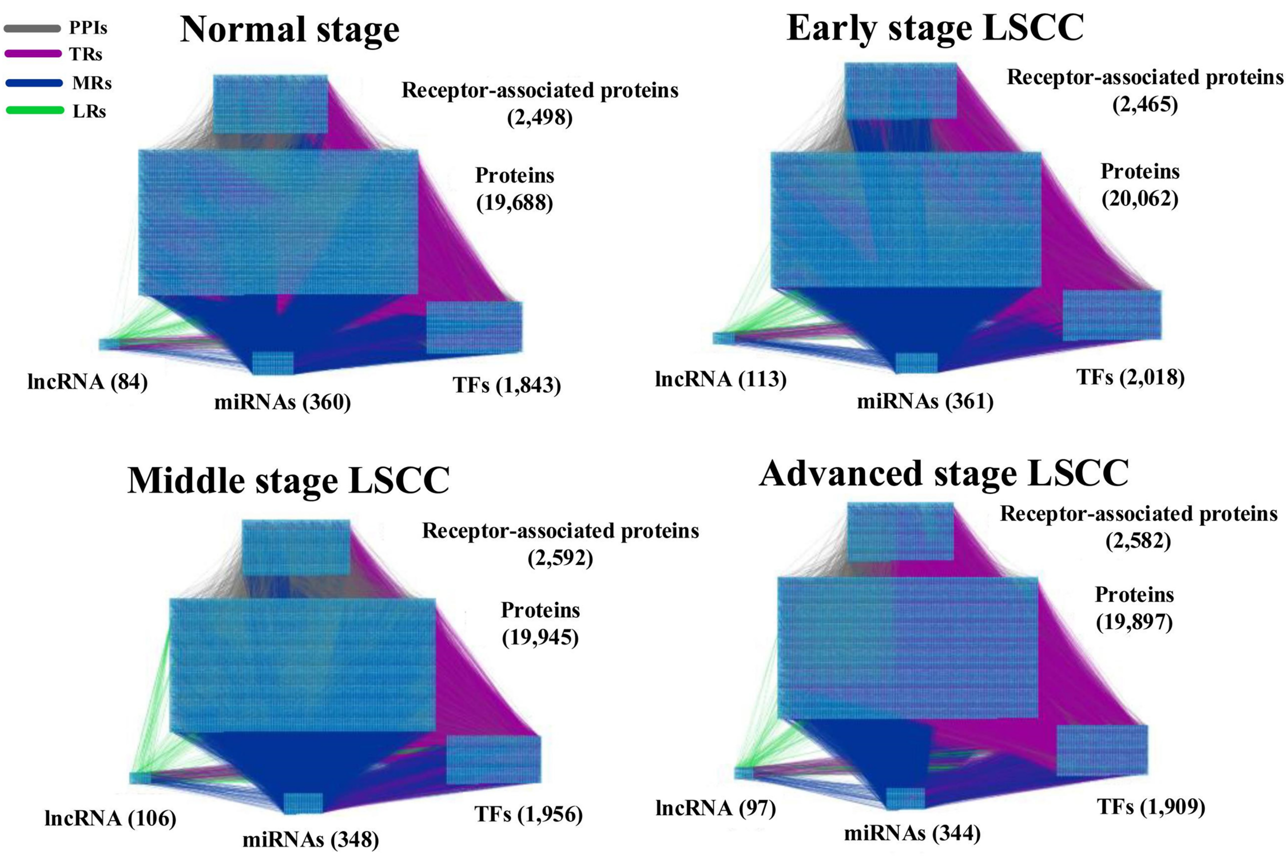

Supplementary Figure 2: The genetic and epigenetic networks (GENs) of normal stage, early stage, middle stage, and advanced stage LSCC. These figures show the identified genetic and epigenetic networks (GENs) of normal lung cells, early stage LADC, middle stage LADC, and advanced stage LADC. The grey lines represent protein-protein interactions (PPIs); the purple lines indicate the transcriptional regulations (TRs); the blue lines denote the miRNA post-transcriptional regulations (MLRs); the green lines are lncRNA regulations (LRs).

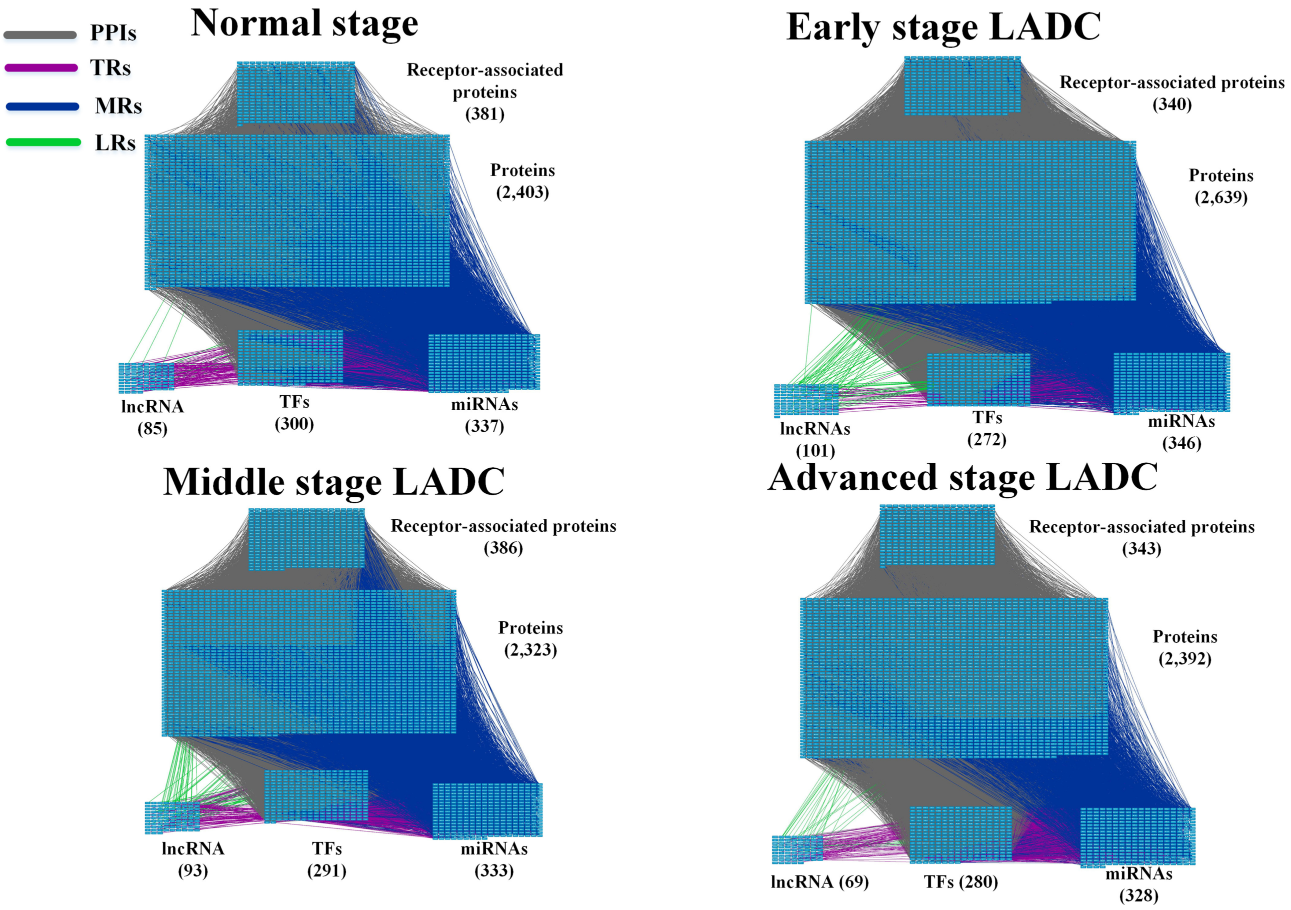

Supplementary Figure 3: The core genetic and epigenetic network (GEN) of normal stage of lung cells near LADC cancer cells. These figures show the identified core genetic and epigenetic networks (GENs) of normal lung cells, early stage LADC, middle stage LADC, and advanced stage LADC. The grey lines represent protein-protein interactions (PPIs); the purple lines indicate the transcriptional regulations (TRs); the blue lines denote the miRNA post-transcriptional regulations (MLRs); the green lines are lncRNA regulations (LRs).

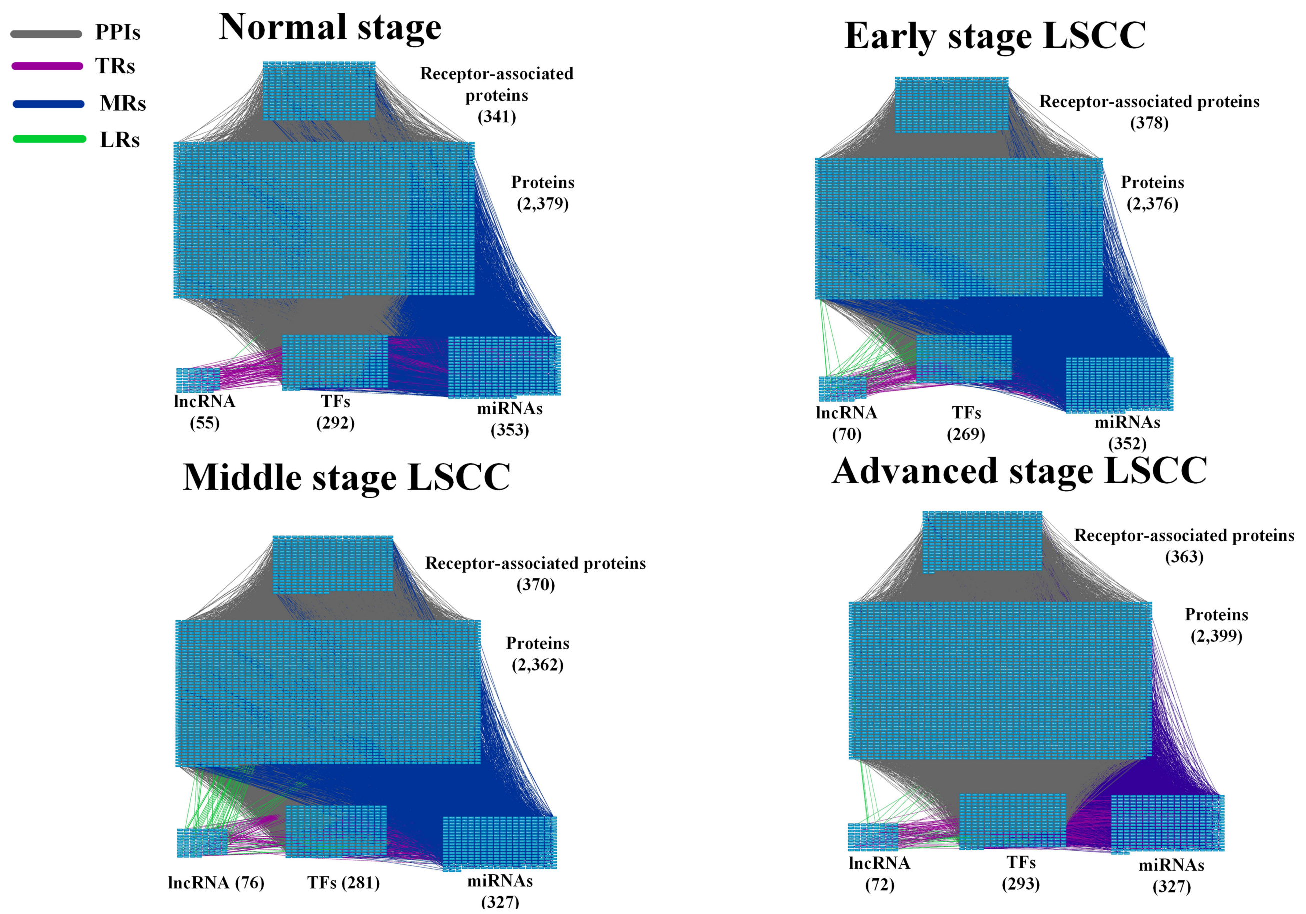

Supplementary Figure 4: The core genetic and epigenetic network (GEN) of normal stage of lung cells near LSCC cancer cells. These figures show the identified core genetic and epigenetic networks (GENs) of normal lung cells, early stage LSCC, middle stage LSCC, and advanced stage LSCC. The grey lines represent protein-protein interactions (PPIs); the purple lines indicate the transcriptional regulations (TRs); the blue lines denote the miRNA post-transcriptional regulations (MLRs); the green lines are lncRNA regulations (LRs).
